# Improving Breast Cancer Outcomes by Enhanced Activities in Early Detection and Diagnosis: An Umbrella Review and Meta-Analyses of Randomised Controlled Trials in High-Income Contexts With Universal Healthcare Coverage

**DOI:** 10.1177/10732748261462921

**Published:** 2026-07-04

**Authors:** Kristoffer Halvorsrud, Helen Fowler, Wasim Hamad, Helen A. Black, Suzanne E. Scott, Rosalind Raine, Elena Pizzo, Oleg Blyuss, Belinda Nedjai, Ranjit Manchanda, Jack Cuzick, Shuping J. Li, Fiona M. Walter, Stephen W. Duffy, Judith Offman

**Affiliations:** 1 Research Department of Primary Care and Population Health, 244880 University College London (UCL), London, UK; 2 Centre for Cancer Screening, Prevention and Early Diagnosis, Wolfson Institute of Population Health, 105714 Queen Mary University of London, London, UK

**Keywords:** breast cancer, screening uptake, help-seeking, umbrella review, meta-analysis

## Abstract

**Introduction:**

Variations in short-term breast cancer survival call for promotion of primary prevention, screening, risk awareness and earlier symptomatic presentation.

**Methods:**

We conducted an umbrella review of published systematic reviews on promotion of screening participation or awareness of and help-seeking behaviours for symptoms with a focus on breast cancer and *transferable* lessons from the other top five male and female adult cancers in the UK (or other high-income countries with universal healthcare). We searched Ovid MEDLINE, EMBASE and Cochrane Database of Systematic Reviews (up until 31.12.25). Two reviewers independently screened 20% of titles and abstracts and all full-texts, as well as carried out all AMSTAR 2 assessments to exclude less informative and ‘critically low’ quality reviews. We extracted information within included reviews on relevant primary studies to conduct random effects meta-analyses.

**Results:**

Forty-three reviews were included, reporting on 168 relevant randomised controlled trials showing significant increases in screening uptake for scheduled versus open appointments (Odds ratio(OR);95% Confidence Intervals(CI)=1.88;1.63,2.17, k=6), advance notification letters versus standard invitation only (OR;95% CI=1.13;1.07,1.20, k=6), letters signed by General Practitioners (OR;95% CI=1.35;1.19,1.53, k=14), educational information (in paper-format) versus no such information (OR;95% CI=1.33;1.05,1.69, k=18), follow-up by calls (OR;95% CI=1.49;1.04,2.14, k=11) or text messages (OR;95% CI=1.28;1.05,1.56, k=7), phone counselling (OR;95% CI=2.63;1.47,4.70, k=4), multi-lingual approaches (OR;95% CI=1.35;1.11,1.63, k=13). Screening uptake was higher in most versus least socially disadvantaged areas for multi-lingual approaches by phone (p=<0.01), follow-up calls (p=<0.01), reminder letters (p=0.03) and ‘implementation intentions’ (goal-directed plans promoting screening) (p=<0.01). Meta-analyses also showed significant improvements for decision aids (paper-format) on knowledge (OR;95% CI=3.88;1.52,9.93, k=7) and informed choice (OR;95% CI=4.97;1.72,14.35, k=4). Statistical heterogeneity was prevalent, potential publication bias and most studies had high or unclear risks of bias.

**Conclusion:**

Some effective interventions were identified; however, information on inequalities across outcomes and interventions targeting awareness and help-seeking behaviours were limited.

## Introduction

Early diagnosis of cancer (and ensuing treatment) is associated with more favourable healthcare outcomes.^1-7^ People living in socially disadvantaged areas and from underrepresented ‘racial’ and ethnic groups are at higher risk of worse breast cancer outcomes, including a higher mortality rate, even though the incidence is lower.^8^ These groups are generally less likely to participate in cancer screening or to present with symptoms outside of routine programmes, which can result in delays in diagnosis^9,10^ However, there are exceptions, for example, in some areas of London screening uptake is higher in more deprived areas.^9^ Barriers to screening attendance or presentation with symptoms in primary care include transport costs (even if universal healthcare is provided), misinformation and mistrust, lack of full knowledge or awareness of screening benefits and symptom manifestations, fear of diagnosis, etc.^4,11,12^ Religion has been identified both as a barrier and a facilitator to attending cancer screening.^13-15^ On one hand, women have described that their religion encouraged a healthy lifestyle,^14^ whereas on the other hand, themes of religious beliefs resulting in fatalism and modesty issues with screening procedures have been identified.^13,15^

Breast cancer screening can either be carried out as an organised programme or opportunistically. For organised screening, individuals within a target population, usually within a defined age group (e.g. 50-70), are actively invited at regular intervals, most commonly every two or three years. Organised screening allows for quality control and evaluation of results.^16^ For some countries, however, opportunistic screening through an unorganised programme or chance encounter may be preferable. In this set-up, individuals in the target group are not systematically invited to screening. The majority of high-income countries with universal healthcare coverage have implemented organised breast screening programmes.^17^ The European Commission Initiative on Breast Cancer (ECIBC) recommends using an organised breast screening programme over opportunistic screening programmes,^18^ and, for example, the UK, Canada, Australia have also implemented organised screening programmes. The United States on the other hand uses opportunistic screening.^17^

In order to improve cancer outcomes as well as provide equity of care, population cancer prevention services should aim for wide-spread reach and encourage participation (while respecting patient autonomy in healthcare decisions^19^). There is therefore a need to identify the most promising interventions to improve cancer prevention and early detection, and to determine whether these interventions may simultaneously reduce inequalities. As the majority of high-income countries have implemented organised breast screening programmes, this review focuses on interventions to be used within this setting.

The initial aim of this review was to identify interventions that could be used to improve breast screening uptake amongst diverse and multiply disadvantaged populations in high-income countries, for whom breast cancer survival is usually lower than average.^20,21^ In particular, the present review sought to identify available information on two types of interventions from published reviews of breast cancer in adults (and other cancers of relevance – see eligibility criteria): (i) promotion of cancer screening participation; or (ii) other interventions that might also reduce cancer risk by promoting awareness and help-seeking behaviours for symptoms. Additionally, where reported, we were interested in information on the extent to which these interventions may reduce inequalities. By including studies on interventions from other common cancers we will increase the usable evidence allowing for subgroup analysis.

## Methods

Our review adopted an umbrella review approach (following Cochrane guidance^22^), focussing on published systematic reviews only. We selected and prioritised information from within the reviews (or their supplementary materials) on individual primary studies based on applicability to our eligibility criteria (see below). Benefitting from and combining available effect estimates across several reviews of relevance to our eligibility criteria, this approach offered a more comprehensive analysis and overview than previous reviews can provide in isolation on either promotions of screening uptake or help-seeking. Furthermore, it also allowed us to draw on transferable lessons across other cancer types than merely breast cancer from high-income countries with universal healthcare such as the UK.

We registered the protocol on PROSPERO [Registration number: CRD42023459690] and followed PRISMA reporting guidelines^23^ as provided in Supplementary file 1.

### Searches and Screening

A systematic search strategy was developed in one electronic database (Ovid MEDLINE) and adapted to other healthcare databases that index most systematic reviews (EMBASE, Cochrane Database of Systematic Reviews), covering literature up until 31.12.25, by KH (see search strategy in Supplementary file 2). The search consisted of a systematic review filter^24^ and a combination of both subject heading and free text terms, of relevance to our eligibility criteria, i.e. for systematic reviews on breast and other relevant cancers as well as interventions for cancer screening uptake and help-seeking.

Records were de-duplicated in EndNote version 20.5^25^ and Rayyan software.^26^ A 20% sample of titles and abstracts were screened in Rayyan software by two independent reviewers [KH and HF]. The two reviewers reached a kappa agreement score of 0.81 and following discussions regarding disagreements in screening decisions, KH screened the remaining titles and abstracts. Relevant records were then screened on full-text by both independent reviewers [KH and HF], with any disagreements in decisions to be discussed and if not resolved, were referred to a third reviewer [SWD].

### Eligibility Criteria

#### Population

We included general adult populations, prioritising breast cancer while transferable lessons from interventions targeting any of the other top five male and female cancers in the UK were also included. For women, these were; lung; bowel; uterine; and skin (melanoma) cancers, while for men: prostate; bowel; lung; skin (melanoma); and head and neck cancers.^27^ Less common cancers were excluded as the challenges and solutions for those are less likely to be relevant to a common cancer such as breast cancer. However, we supplemented the above with a specific, targeted search and inclusion of cervical cancer due to the success of its screening programmes^28^ and potential relevance for breast cancer.

#### Context

Intervention delivered through any formats (i.e. clinical, online, community) were included.

We only included cited primary studies that contained high-income countries (as per the World Bank’s classification^29^) with universal healthcare coverage such as e.g. in Canada, Australia and certain EU/EEA countries.^30-32^ We excluded high-income countries without universal healthcare coverage and low- or middle-income countries.

#### Interventions

We included two main intervention categories: (i) promotion of cancer screening participation; or (ii) other interventions that might also reduce cancer risk by promoting awareness and help-seeking behaviours for symptoms. We excluded literature without an evaluation of intervention(s), such as only describing well-established, proof-of-principle of preventive measures (e.g. association of diet with cancer risk), or interventions that exclusively focused on the treatment/care of patients that have already been diagnosed with cancer. However, we included interventions such as formal risk assessment if embedded into screening programmes, either as a tool to stratify screening regimens or to inform selection for primary prevention advice.

#### Comparator(s)

We specified that reviews should report post-intervention data of relevant primary studies to compare the intervention with at least one comparison condition (which might be a control group, or also refer to any reported or measured changes after vs. before the intervention was implemented in a single intervention group (e.g. pre-post studies)). On this assumption, we excluded studies for which reviews have reported insufficient post-intervention data to compare the intervention with at least one comparison condition.

#### Types of Studies

We included peer-reviewed publications labelled as ‘systematic reviews’ or ‘meta-analyses’ on title or abstract in the first instance. For any protocol, pre-print or conference paper with such labels, they were included for subsequent tracing of any peer-reviewed publications of relevant data.

On full-text screening, as it is recognised that some publications labelled as ‘systematic reviews’ or ‘meta-analyses’ might not qualify as such, we also referred to the relevant Cochrane Handbook criteria (Chapter 1, Section 1.1).^33^ For ‘meta-analyses’ to be included at this stage, they also needed to fulfil these criteria (e.g. comprehensive, systematic search).

We considered systematic reviews of both randomised controlled trials (RCTs) and non-randomised studies due to our focus on health inequalities. If a systematic review excluded meta-analysis, it needed to report a measure or category of effect size of their included studies.

Non-systematic reviews, editorials, opinion pieces and non-English literature were excluded.

#### Outcome Measures

Outcomes could relate to relevant data for a number of measures including: screening uptake; referral rates; time from symptom detection to presentation or diagnosis; stage of disease at diagnosis; knowledge or awareness of cancer symptoms and risks (e.g. from reports or scores on scales); frequency of self-checking for cancer symptoms; confidence or intentions to seek help in the event of noticing a cancer symptom; active help-seeking behaviour as reflected in e.g. number of service contacts or visits; tackling of wider determinants to cancer detection.

### Data Extraction

Data was extracted by KH (and checked by either HF, JO or SWD) from included reviews and their supplementary materials including relevant primary studies they cited. We extracted data on study characteristics (authors; title; year of publication; setting; study design), participant characteristics (sample number; age; gender; socioeconomic indicators; ethnicity; religion; cancer types targeted), interventions, comparators, primary, secondary (etc.) outcomes, risk of bias tools used by review and the corresponding primary study assessed scores on respective domains.

### Quality Assessment

Two independent reviewers [KH and SWD+WH] used AMSTAR 2,^34^ to assess all potentially included reviews subsequent to full-text screening, with any discrepancies resolved through discussion, but if unresolvable were to be adjudicated by a third independent reviewer (see AMSTAR 2 assessments in Supplementary file 3). We selected certain AMSTAR 2 domains to inform which reviews to prioritise that allowed for assessment of applicability and methodological quality of relevant primary studies they cited: i.e. item number 8 (‘Did the review authors describe the included studies in adequate detail?’) and 9 (‘Did the review authors use a satisfactory technique for assessing the risk of bias in individual studies?’).^34^ Then to further determine quality, we followed AMSTAR 2 recommendations to also interpret as ‘critical’ item number 2 (‘Protocol registration’); 4 (‘Literature search’); 7 (‘Justification for excluding studies’); and 13 (‘Consideration of risk of bias when interpreting results’).^34^ Based on this, any reviews of ‘critically low’ quality^34^ were excluded.

### Data Synthesis

A narrative synthesis^35^ proceeded and where sufficient and comparable data, random effects meta-analyses (of primary studies as reported in the reviews) organised by intervention type and outcome in Stata (version 18)^36^ with Knapp-Hartung adjustments of 95% confidence intervals^37^ [by KH].

For pooling of data, we prioritised estimation of odds ratios (ORs) where applicable. However, where relevant data for the same outcome was reported in multiple effect size formats, we applied appropriate formula^38^ for conversion to ORs from other effect size formats. Rather than sole reliance on the prospective variations in effect size calculations across studies, we prioritised self-calculation of raw data where reported in systematic reviews. When only already calculated estimates were reported, we prioritised unadjusted rather than adjusted estimates when both were reported (as the level and type of adjustments might also vary considerably across studies).

To accurately capture the volume of evidence contributing to each respective meta-analysis, we refer to effect measures (denoted by k=) rather than studies as reviews would sometimes report on multiple effect estimates emanating from the same study related to different population groups, for example. Additionally, for some estimates, information to calculate ORs was extracted across multiple included reviews as they reported complementary information for the calculation of these same estimates. However, in all cases we ensured not to double-count any estimates by using an Excel matrix mapping out the overlap of any primary study references included by multiple reviews2^22^ (see Supplementary file 4).

If appropriate and the data allowed the performance of meta-analyses, within- and between-group statistical heterogeneity (measuring the extent to which studies in meta-analysis had varying effects) was also assessed through Cochran’s Q, its degrees of freedom (df) and P-value, as well as the between-study variance (τ^2^). To assess potential sources or reasons for heterogeneity, we conducted subgroup analyses where appropriate on:

• Cancer type targeted;
• Age (divided into ‘under 50 years’, ‘50-59 years’, ‘60-69 years’ and ‘70+ years’ depending upon mean age of participants, or if not reported, the mid-point of the age range4);^39^
• Gender;
• Index of Multiple Deprivation (IMD) score (highest vs. lowest deprivation);
• Ethnic groups considered (people from ‘racial’ and ethnic groups that are underrepresented vs. that are part of the majority group);
• Geographical context (divided into the UK, rest of Europe and the rest of the world).

We also considered religion, but had to preclude this as a subgroup analysis as there was no information on this in relation to any of the reported primary studies.

Potential sensitivity analyses were conducted related to studies’ risks of bias (as reported by authors in the included reviews using guidance thresholds as specified in^40^ for the Risk of Bias (RoB) tool version 2 and in Table 8.7.a^1^ in^41^ for version 1 of this tool), ranking studies as ‘high’, ‘unclear’ (labelled as ‘some concerns’ for RoB-2) or ‘low’ risks of bias. We prioritised risk of bias assessments in higher quality (AMSTAR 2) reviews if reported across multiple reviews).

We further assessed prediction intervals and assessed funnel plots for main meta-analyses containing at least ten studies.^37^

## Results

**Figure 1.**
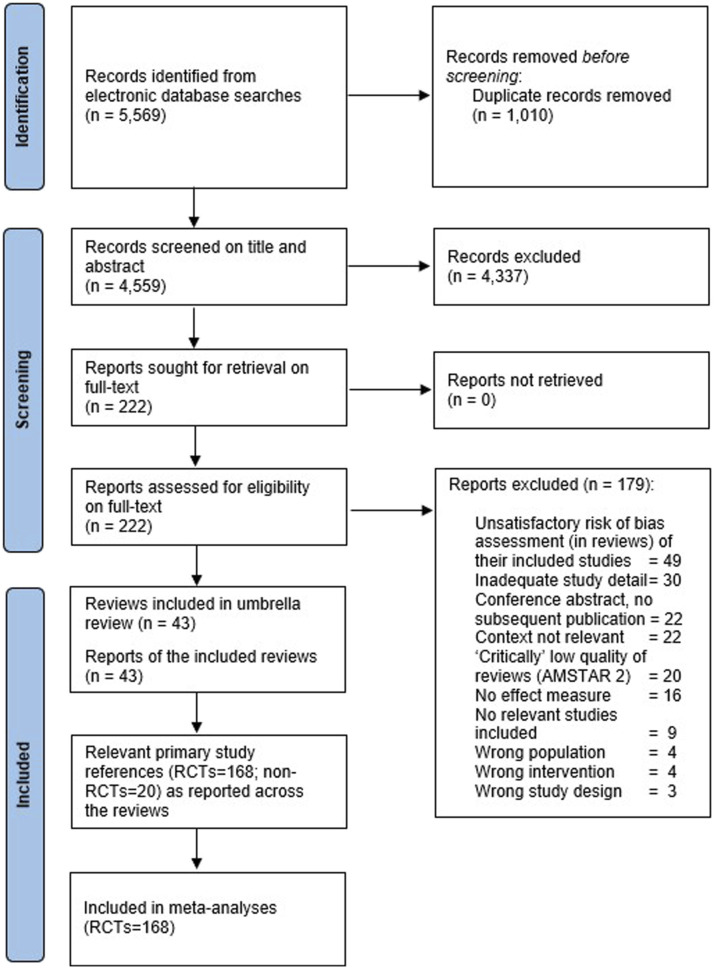
PRISMA flow diagram illustrating initial search and study selection process, including duplicate removal, screening, eligibility assessment, and final inclusion of studies.

Figure 1 shows the PRISMA flow diagram of the searches and screening process for the umbrella review. Based on searches, 4,393 records were identified. Title and abstract screening resulted in 216 potentially relevant records that were screened on full-text. A total of 43 systematic reviews were included^42-84^ (of which 30 reviews^42,44-46,48,51-62,64,66,68,69,73,75,76,78-82,84^ also incorporated separate meta-analyses). Table 1 shows the key characteristics of the included reviews, while Table 2 illustrates how the majority of included reviews were published in the most recent five-year-gaps between 2016-2020 and 2021-2025. Reasons for exclusion for each full-text reference are provided in Supplementary file 5. The majority of relevant effect estimates reported in the 43 included reviews were from RCTs (n=168). Although the review protocol specified that non-RCTs were also to be included, the relatively few relevant non-RCTs (k=20) reported across reviews would not have significantly altered overall reported effects. As such, we only report on effects from meta-analyses of RCTs below (however, in Supplementary file 6 key characteristics of both the RCTs contributing to meta-analyses and the non-RCTs are provided). Of the 168 RCTs in the meta-analyses, 39 reported results from the UK, 64 from other European countries and 65 from non-European countries. Outcomes were reported for different cancer types (to note that in some studies outcomes were reported across multiple cancers): colorectal (k=61); cervical (k=58); breast (k=30); prostate (k=13); skin (k=7); lung (k=2). Considering review authors’ reported risk of bias assessments, only 27 of the 168 RCTs were of low risk of bias (see Supplementary file 6).

**Table 1.** Key Characteristics of Included Reviews

Authors.	Databases searched	Last search (date)	Included studies (relevant to our meta-analyses*)	Relevant country/ies included	Cancer type(s)	Intervention category/ies	Outcome(s)
Abdul Latip et al 2022^74^	EmBASE, Medline, PsychInfo, Scopus, CINAHL	17-Nov-20	42 (1)	New Zealand	Colorectal	Multi-lingual approaches	Screening uptake
Ahadinezhad et al 2024^79^	PubMed, Scopus, Cochrane	2022	38 (10)	UK, England, Scotland, Australia, Canada, Cyprus, Denmark, Germany, Israel, Spain	Colorectal	Decision aids, Educational information, Implementation intention, Letters, Survey	Screening uptake
Alam et al 2023^80^	Pubmed, Scopus, EMBASE, CINAHL, PsycINFO, CENTRAL, ERIC	12-Oct-21	42 (3)	Hong Kong, Norway	Cervical	Multi-lingual approaches	Screening uptake
Ampofo et al 2022^76^	Medline, EMBASE, CENTRAL, PsycINFO, CINAHL, Web of Science, ERIC	Nov-20	13 (2)	UK, Canada	Cervical	Educational information	Knowledge, Risk perception
Baptista et al 2018^55^	PubMed, CINAHL, PsycINFO, CENTRAL	Nov-16	7 (2)	Wales, Australia	Prostate	Educational information	Screening uptake, Decisional conflict, Knowledge
Brouwers et al 2011^47^	MEDLINE, EMBASE, CINAHL, PsycINFO	May-10	66 (10)	England, Australia, Canada, Denmark, Italy, Spain, Taiwan	Breast, Cervical, Colorectal	Counselling, Educational information, Letters, Phone follow-up, Targeting GPs	Screening uptake
Camilloni et al 2013^42^	PubMed, EMBASE, Cochrane, PsycINFO, LILACS, HTA, CRD	Jul-12	69 (28)	UK, Australia, Finaland, France, Germany, Italy, Netherlands, New Zealand, Singapore, Spain, Sweden	Breast, Cervical, Colorectal	Appointment (scheduled vs. open), Choice of methods, Educational information, Home visits, Letters, Multi-lingual approaches, Phone follow-up	Screening uptake
Chambergo-Michilot et al 2020^78^	PubMed, CENTRAL, Scopus	Dec-18	5 (2)	Australia	Skin	Phone follow-up	Knowledge, Self-examination
Chan and So 2021^67^	Medline, PsycINFO, PubMed, Scopus	Nov-19	6 (1)	France	Colorectal	Counselling	Screening uptake
Costa et al 2022^62^	PubMed, Embase	31-Mar-22	33 (21)	UK, Australia, Belgium, Canada, Denmark, Italy, Netherlands, New Zealand, Norway, Finland, France, Sweden	Cervical	Choice of methods	Screening uptake
Dawkins et al 2025^84^	Medline, Embase, CINAHL, Cochrane, PsycINFO, Web of Science, Scopus	26-July-25	40 (1)	UK	Lung	Educational information	Screening uptake, Knowledge, Decisional conflict
Dieng et al 2014^59^	Medline, PsycINFO, Allied and Complementary Medicine, CINAHL, Embase	Jan-13	40 (6)	UK, England, Wales, Netherlands, Sweden	Breast, Skin	Educational information, Risk assessment	Screening uptake, Knowledge, Risk perception, Risk accuracy
Edwards et al 2013^45^	CENTRAL, MEDLINE, EMBASE, CINAHL, PsycINFO	Mar-12	38 (4)	England, Australia, Germany	Cervical, , Colorectal, Skin	Educational information, Decision aids	Screening uptake, Knowledge, Informed choice, Risk perception
Elepaño et al 2021^53^	PubMed, Scopus, ClinicalTrials.gov	Oct-20	4 (2)	England, Spain	Colorectal	Phone follow-up	Screening uptake
Ersser et al 2019^57^	Cochrane Library, EMBASE, MEDLINE, CINAHL, PsycINFO, Web of Science, MEDLINE	Apr-18	9 (2)	Australia	Skin	Educational information, Phone follow-up	Screening uptake, Knowledge, Self-examination
Goodwin et al 2019^48^	PubMed, PsycINFO, Scopus, CINAHL, ProQuest Dissertations and Theses A & I, Google Scholar	10-Mar-18	30 (21)	UK, England, Wales, Scotland, North Ireland, Australia, Netherlands, Israel, Spain, Latvia	Colorectal	Educational information, Letters, Phone follow-up, Simplified methods, Time to complete	Screening uptake
Henrikson et al 2018^58^	CENTRAL, MEDLINE, PubMed	01-Feb-17	21 (4)	England, Australia, France	Skin	Counselling, Educational information, Phone follow-up	Screening uptake, Knowledge, Self-examination
Ilic et al 2015^72^	Medline, EMBASE, CINAHL, PsycINFO, Cochrane Database of Systematic Reviews, DARE, Health Technology Assessment	Mar-14	13 (4)	Wales, Australia	Prostate	Decision aids, Educational information	Screening uptake Knowledge, Decisional conflict, Risk perception
Issaka et al 2019^49^	PubMed, Embase, CINAHL, Web of Science	13-Dec-17	20 (5)	Australia, Italy, Netherlands, France, Spain	Colorectal	Letters, Targeting GPs	Screening uptake
Ivlev et al 2018^56^	MEDLINE, Scopus, CENTRAL, CT.gov, Cochrane report, PsycARTICLES, PsycINFO	06-Apr-18	16 (6)	UK, Wales, Australia, France	Prostate	Decision aids, Educational information	Screening uptake, Screening intention, Knowledge, Decisional conflict, Risk perception
Jepson et al 2001^65^	MEDLINE, BIDS Science Citation Index, BIDS Social Science Index, Econlit, EMBASE, Cancerlit, DHSS data, Dissertation Abstracts, ERIC, HealthStar, ASSIA, Pascal, SIGLE, CINAHL, Sociofile, PsycINFO, SHARE (Kings Fund), Library of Congress database, NHS CRD DARE, Cochrane Database of Systematic Reviews, CENTRAL, the National Research Register, PsycLIT	Aug-2000	6 (1)	Canada	Prostate	Educational information	Screening uptake, Decisional conflict
Larsen et al 2021^71^	PubMed, CINAHL, PsycINFO, Embase, Scopus	19-Mar-21	14 (3)	Australia, Denmark	Colorectal	Decision aids	Screening uptake, Knowledge, Decisional conflict, Informed choice, Attitudes
Lau et al 2022^60^	PubMed, CINAHL, Web of Science, Embase, CENTRAL	Sep-22	14 (3)	Australia, Canada, Denmark	Colorectal	Decision aids, Educational information	Screening uptake Knowledge, Decisional conflict, Attitudes
Li et al 2020^64^	British Nursing Index, CENTRAL, CINAHL Complete, EMBASE, Ovid Emcare, Medline, Scopus, Web of Science Core Collection, Chinese Biomedical Literature Databases, China Journal Net, Wanfang Data	Sep-19	10 (2)	Canada, France	Colorectal	Couselling, Patient navigation, Simplified methods	Screening uptake
Liu et al 2024^81^	MEDLINE, Cochrane Central Registry of Controlled Trials, Embase, PsycINFO, PubMed, Scopus, Web of Science, Cumulative Index to Nursing and Allied Health Literature	29-Dec-23	15 (4)	England, Scotland, Portugal, Spain	Cervical	Choice of methods, Educational information, Phone follow-up	Screening uptake
Martínez-González et al 2018^73^	MEDLINE, EMBASE, CINHAL, Cochrane Library, PsychINFO, Scopus	Mar-15	4 (1)	Australia	Prostate	Educational information	Screening uptake, Knowledge, Decisional conflict, Risk perception
McAlpine et al 2018^68^	MEDLINE, Embase, PsycINFO, CINAHL, CENTRAL	Apr-15	46 (10)	UK, Wales, Australia, Germany	Breast, Colorectal, Prostate	Decision aids	Knowledge, Decisional conflict
Musa et al 2017^46^	Pubmed, Embase, Cochrane Systematic Reviews, CENTRAL	Aug-16	28 (13)	Australia, Belgium, Canada, Finland, France, Germany, Italy, Japan, Sweden, Taiwan	Cervical	Choice of methods, Educational information, Letters, Phone follow-up	Screening uptake
Ramli et al 2021^54^	MEDLINE, Web of Science, Cochrane Library	04-Dec-20	10 (1)	New Zealand	Colorectal	Educational information	Screening uptake
Rat et al 2018^50^	PubMed, Embase, Cochrane Library	01-Sep-15	24 (16)	UK, Australia, Canada, France, Israel, Italy, Netherlands, Spain	Colorectal	Choice of methods, Educational information, Letters, Phone follow-up, Simplified methods, Targeting GPs	Screening uptake
Riganti et al 2024^82^	Cochrane Breast Cancer Group’s Specialised Register, CENTRAL, MEDLINE, Embase, CINAHL, PsycINFO, ClinicalTrials.gov, WHO International Clinical Trials Registry Platform	08-Aug-23	19 (7)	Australia, Italy, Germany, Netherlands, Spain	Breast	Decision aids, Educational information	Knowledge, Informed choice, Decisional conflict, Proportion undecided, Risk perception, Confidence, Anxiety
Riikonen et al 2019^66^	MEDLINE, Embase, CINAHL, PsychINFO, CENTRAL	19-Jun-18	19 (3)	UK, Canada	Prostate	Couselling, Educational information	Screening uptake, Knowledge, Decisional conflict
Rodriguez-Gomez et al 2020^63^	Medline, CINAHL, Embase	Dec-17	20 (2)	Australia, Canada	Breast, Cervical	Choice of methods, Family physician, Mass media campaign	Screening uptake
Ruco et al 2021^75^	MEDLINE, Embase, PsycINFO, Scopus, CINAHL, CENTRAL, Communication & Mass Media Complete	17-Jul-20	39 (7)	UK, England, Israel, Portugal, Spain	Breast, Cervical, Colorectal	Phone follow-up	Screening uptake
Saab et al 2021^77^	MEDLINE, CINAHL, ERIC, APA PsycARTICLES, APA PsycInfo and Psychology, Behavioral Sciences Collection	15-Jan-20	16 (2)	UK, Australia	Lung	Educational information	Knowledge, Decisional conflict, Follow-up consultations, Time to first consultation
Schliemann et al 2019^43^	Medline, EMBASE, CINAHL, Web of Science, Cochrane Library, Google Scholar	Sep-17	22 (6)	Israel, Japan, Singapore, South Korea, Taiwan	Breast, Cervical, Colorectal	Educational information, Home visits, Letters, Phone follow-up	Screening uptake, Stage 0 or 1 cancer diagnosis
Stacey et al 2024^69^	CENTRAL, MEDLINE, Embase, PsycINFO, EBSCO	Mar-22	209 (11)	UK, Wales, Australia, Germany, Italy, Spain	Breast, Colorectal, Prostate	Decision aids	Knowledge, Informed choice, Decisional conflict, Proportion undecided, Risk perception
Staley et al 2021^44^	CENTRAL, MEDLINE, Embase	08-Jun-20	70 (26)	UK, Australia, Canada, Denmark, France, Germany, Italy, Norway, Spain, Sweden	Cervical	Appointment (scheduled vs. open), Counselling, Educational information, Home visits, Letters	Screening uptake
Teo et al 2018^52^	PubMed, Embase, CINAHL, PsycINFO, Web of Science	19-Oct-15	58 (16)	UK, Wales, Australia, France, Greece, Ireland, South Korea	Prostate, Skin	Counselling, Decision aids, Educational information, Implementation intention	Screening uptake, Screening intention
Tsipa et al 2021^51^	Embase, PsycINFO, MEDLINE	15-May-16	102 (17)	UK, England, Scotland, Australia, Canada, France, Israel, Netherlands, Poland, Spain	Colorectal	Choice of methods, Couselling, Decision aids, Educational information, Implementation intention, Letters, Multi-lingual approaches, Phone follow-up, Simplified methods, Survey	Screening uptake
van Agt et al 2017^70^	Medline, EMBASE, Cochrane databases	Apr-12	15 (5)	Australia, Denmark, Germany	Breast, Cervical, Colorectal	Choice of methods, Decision aids, Educational information	Screening uptake, Knowledge, Informed choice, Decisional conflict, Proportion undecided, Risk perception, Attitudes, Anxiety
Verbunt et al 2024^83^	Medline, EMBASE, CINAHL	23-Nov-23	49 (5)	Australia, France, Norway, Spain	Cervical, Colorectal	Multi-lingual appraoches, Targeting GPs	Screening uptake
Verdoodt et al 2015^61^	Medline (PubMed), EMBASE, CENTRAL	N/A	16 (10)	UK, Finland, France, Italy, Netherlands, Sweden	Cervical	Choice of methods	Screening uptake

^*^To note that some studies were reported across multiple reviews.

**Table 2.** Reviews by Publication Years

Year range of publications	N of systematic reviews	References (by year of publication)
2001-2005	1	2001: Jepson et al^65^
2006-2010	0	N/A
2011-2015	6	2011: Brouwers et al;^47^
		2013: Camilloni et al;^42^ Edwards et al;^45^
		2014: Dieng et al;^59^
		2015: Ilic et al;^72^ Verdoodt et al^61^
2016-2020	17	2017: Musa et al;^46^ van Agt et al;^70^
		2018: Baptista et al;^55^ Henrikson et al;^58^ Ivlev et al;^56^Martínez-González et al;^73^ McAlpine et al;^68^ Rat et al;^50^ Teo et al;^52^
		2019: Ersser et al;^57^ Goodwin et al;^48^ Issaka et al;^49^ Riikonen et al;^66^ Schliemann et al;^43^
		2020:Chambergo-Michilot et al;^78^ Li et al;^64^ Rodriguez-Gomez et al^63^
2021-2025	19	2021: Chan and So6;^67^ Elepaño et al;^53^ Larsen et al;^71^ Ramli et al;^54^ Ruco et al;^75^ Saab et al;^77^ Staley et al;^44^ Tsipa et al;^51^
		2022: Abdul Latip et al;^74^ Ampofo et al;^76^ Costa et al;^62^ Lau et al;^60^
		2023: Alam et al;^80^
		2024: Ahadinezhad et al;^79^ Liu et al;^81^ Riganti et al;^82^ Stacey et al;^69^ Verbunt et al;^83^
		2025: Dawkins et al^84^

**Figure 2.**
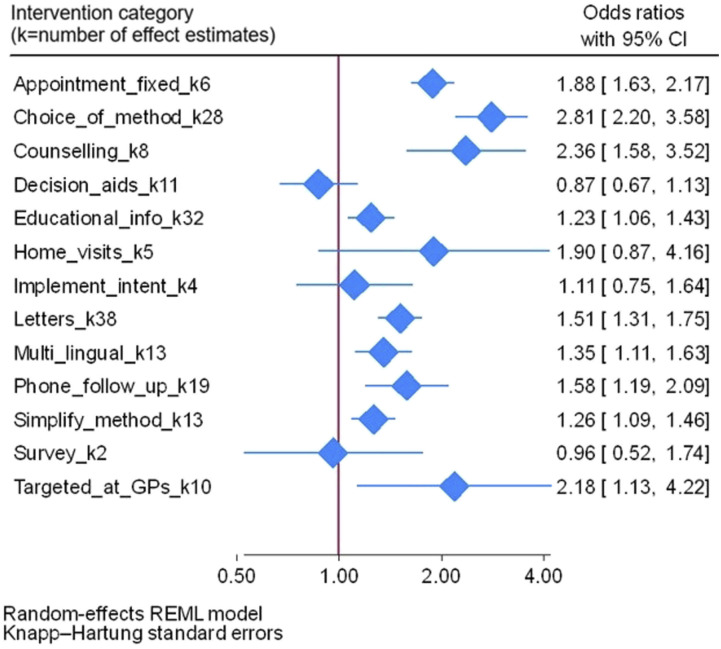
**Random effects meta-analyses of primary studies for screening uptake. **Forest plot where each row represents a specific intervention type, with the number of effect estimates included indicated by k. Diamonds represent pooled effect estimates, with horizontal lines indicating 95% CIsAll estimates were derived using random-effects with restricted maximum likelihood as the estimation platform with Knapp–Hartung standard errors.

**Figure 3.**
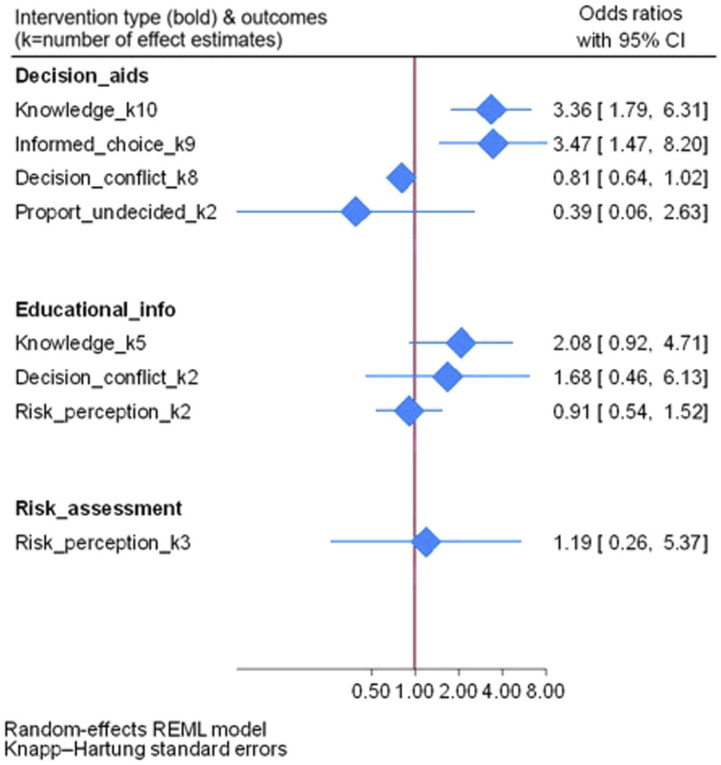
**Random effects meta-analyses of primary studies for other outcomes.** Forest plot where each row represents a specific intervention type, with the number of effect estimates included indicated by k. Diamonds represent pooled effect estimates, with horizontal lines indicating 95% CIs. All estimates were derived using random-effects with restricted maximum likelihood as the estimation platform with Knapp–Hartung standard errors.

We present meta-analyses of identified interventions alphabetically and the outcomes for which they are relevant: primarily on screening uptake with the largest volume of evidence, but for each intervention also comment on other outcomes (including of relevance to earlier symptomatic presentation and diagnosis) and highlight any divergences in results or gaps in the evidence base. We have omitted discussion of standard invitation letters in other contexts, as these are established practice in organised breast screening programmes, like the UK’s breast cancer screening programme, and recommended by ECIBC guideline recommendations.^18,85^ Figures 2 and 3 present summary forest plots of the main meta-analyses for screening uptake and other outcomes, respectively. Due to our focus on inequalities, we also present a table with all the available data and corresponding analyses specifically by ethnicity and area deprivation across the intervention categories and outcomes (see Table 3). We further refer readers to Supplementary file 7 (for screening uptake) and Supplementary file 8 (for other outcomes) presenting the full statistics of any meta-analyses, heterogeneity, prediction intervals (where relevant) as well as all subgroup and sensitivity analyses. Supplementary file 9 contains the funnel plots for small study effects.

**Table 3.** Impact of Interventions by Ethnicity and Area Deprivation

​	**Improved for underrepresented ‘racial’ and ethnic vs. the majority group.**	**Improved for underrepresented ‘racial’ and ethnic groups(but no comparisons)**	**No significant** * **differences/changes**	**Improved for the majority vs. underrepresented ‘racial’ and ethnic groups**	**Worsened for underrepresented ‘racial’ and ethnic groups (but no comparisons)**
**Ethnicity**	*Screening uptake:* • Choice of methods (k=7); • Simplified methods (k=2).	*Screening uptake:* • Counselling by phone (k=1); • Multi-lingual approaches by phone (k=4).	*Screening uptake:* • Decision aids (printed format) (k=2); • Educational information (printed unspecified literacy) (k=6); • Home visits (k=4); • Invitation letters (k=2); • Personalised letters (k=1); • Multi-lingual approaches (face-to-face k=6 + written material k=2); • Phone follow-up calls (k=4).	N/A.	N/A.
​	**Improved in most deprived vs. least deprived area**	**Improved in most deprived area (but no comparisons)**	**No significant** * **differences/changes**	**Improved in least deprived vs. most deprived area**	**Worsened in most deprived area (but no comparisons)**
**Area deprivation**	*Screening uptake:* • Implementation intention (k=2); • Reminder letters (k=2); • Multi-lingual approaches by phone (k=3); • Phone follow-up calls (k=2).	*Screening uptake:* • Multi-lingual approaches (face-to-face) (k=1). *Knowledge:* • Decision aids (combined printed and online) (k=1); • Educational information (combined printed and video) (k=1). *Informed choice:* • Decision aids (combined printed and online) (k=1).	*Screening uptake:* • Appointment (scheduled) (k=1); • Choice of methods (k=7); • Educational information (printed unspecified literacy k=4 + low literacy k=2 + online k=2); • Advance notification letters (k=3); • Letters signed by GPs (k=3); • Personalised letters (k=2); • Phone follow-up text messages (k=4); • Simplified methods (k=4); • Alerts targeted at GPs (k=4). *Decisional conflict:* • Educational information (combined printed and online) (k=1).	*Screening uptake:* • Survey (k=2).	*Screening uptake:* • Decision aids (combined printed and online) (k=1). *Attitudes towards screening:* • Decision aids (combined printed and online) (k=1).

^*^if defining significance at p-value<0.05; k=number of effect estimates (for full break-down of how these are distributed across subgroups and corresponding results of subgroup analyses, readers are referred to Supplementary files 7 and 8).

### Appointments

#### Screening Uptake

Two systematic reviews^42,44^ reported six effect estimates on cancer screening appointments with a scheduled date and time, compared with no such fixed date and time (‘open’), on screening uptake. Our meta-analysis showed a significantly higher increase in screening uptake for scheduled appointments (OR; 95% CI=1.88; 1.63, 2.17), with no considerable statistical heterogeneity (as particularly suggested by the Tau^2^ statistics of <0.01) (see Supplementary file 7). This may indicate that the studies in this meta-analysis presented relatively similar results.

#### Other Outcomes

Due to lack of evidence, no meta-analyses could be conducted for appointments on any other outcomes.

### Choice of Methods

#### Screening Uptake

Estimates on the access to or the option of more than one method to screen for cancers, emanate primarily from other cancers (cervical, colorectal) where self-tests are prominent. Seven systematic reviews^42,44,46,61-64^ provided 28 such estimates (originating from 25 studies) revealing a significant increase in uptake (OR; 95% CI=2.81; 2.20, 3.58). There was higher uptake in people from ‘racial’ and ethnic groups that are underrepresented vs. that are part of the majority group (p=0.01).

#### Other Outcomes

Due to lack of evidence, no meta-analyses could be conducted for choice of methods on any other outcomes.

### Counselling With Healthcare Professionals

#### Screening Uptake

In this paper counselling is defined as the option for undecided screeners to discuss the screening process and its benefits and any challenges with a healthcare professional. It includes studies where counselling was delivered in different formats (face-to-face in the clinic, by phone). Altogether, regardless of the format, nine systematic reviews^43,44,47,51,52,64-67^ reported on eight estimates. This showed a significant increase in screening (OR; 95% CI=2.36; 1.58, 3.52). By separating different formats of counselling (at the clinic or by phone), the significant effect was retained in phone counselling (OR; 95% CI=2.63; 1.47, 4.70) based on four estimates reported in five reviews.^43,47,52,64,67^ Highest uptake was indicated in those aged 60-69 years compared to younger cohorts (p=<0.01).

#### Other Outcomes

Whether patients would increasingly examine themselves for potential cancer symptoms was also addressed. A significant increase in skin self-examination was detected for the provision of counselling at the clinic, although only based on one estimate (from one review^58^) (Supplementary file 8).

### Decision Aids

#### Screening Uptake

Altogether, regardless of format, 13 systematic reviews^45,51,52,55,56,60,66,68-73^ reported eleven estimates for the effect of decision aids (printed, online, or both combined), showing non-significant results (overall OR; 95% CI=0.87; 0.67, 1.13) and statistical heterogeneity. The funnel plot showed a diverse spread of studies (asymmetry) and suggested that publication bias *might *be a factor (Supplementary file 9).

#### Other Outcomes

Regardless of decision aid format, ten estimates from eleven reviews^45,52,56,66,68-73,82^ suggested improved knowledge of cancer risks and screening benefits (OR; 95% CI=3.36; 1.79, 6.31). Albeit statistical heterogeneity with varied individual effects were indicated, as well as funnel plot outliers (see Supplementary file 9). Based on seven estimates from the eleven reviews,^45,52,56,66,68-73,82^ significance was retained (OR; 95% CI=3.88; 1.52, 9.93), but heterogeneity of individual estimates not reduced for decision aids in paper-format. Higher knowledge scores were detected in breast and colorectal cancer programmes (p=<0.01 relative to comparators) (Supplementary file 8).

For whether patients felt they had made an informed choice when deciding whether to screen, positive effects were seen for decision aids of any format (OR; 95% CI=3.47; 1.47, 8.20, k=9 from six reviews^45,68-71,82^) and for the four measures specifically for printed decision aids (OR; 95% CI=4.97; 1.72, 14.35). Analyses had relatively high heterogeneity of individual results.

Of further note, regardless of decision aid format, eight estimates across thirteen reviews^35,38,39,43,49,51-56,62,75^ also considered scores on a decisional conflict scale (level of conflict patients may express on whether to undergo screening), showing a non-significant result (OR; 95% CI=0.81; 0.64, 1.02) (Supplementary file 8).

### Educational Information

#### Screening Uptake

Irrespective of delivery format, 21 reviews^42-60,81,84^ reported on 32 estimates for the effect of providing educational information, such as the procedure and benefits of screening, with an overall positive effect (OR; 95% CI=1.23; 1.06, 1.43) – albeit heterogeneity and some funnel plot asymmetry indicating that publication biasmight be present. For educational information in printed or paper-format aimed at a general literacy level, eleven reviews^42-52^ reported on 18 estimates retaining the positive effect (OR; 95% CI=1.33; 1.05, 1.69), but still some funnel plot asymmetry (albeit less so; see Supplementary file 9). To note is the higher uptake in those under 50 years (p=<0.01). Two estimates reported across two reviews^48,51^ also considered printed educational material specifically designed for a low literacy audience; however, with a non-significant result (OR; 95% CI=1.03; 0.95, 1.11). Non-significant results were observed for educational formats delivered through: video (OR; 95% CI=1.06; 0.76, 1.49) including seven estimates considered across twelve reviews^42,47,50-52,54-58,81,84^; and the Internet (OR; 95% CI=0.99; 0.18, 5.61) with two estimates considered across five reviews.^51,52,55,56,60^

#### Other Outcomes

Regardless of format, five estimates across six reviews^45,58,59,76,77,82^ also considered the impact of educational information on knowledge with a non-significant result (OR; 95% CI=2.08; 0.92, 4.71). However, significantly improved knowledge was suggested for three separate formats (albeit only one study contributing to each): online; a computer programme; paper-format combined with video (delivered in an area characterised by high deprivation in the UK (see Supplementary file 8)).

### Home Visits to Promote Participation

#### Screening Uptake

Three systematic reviews^42-44^ reported on five estimates where patients due for screening were visited by healthcare professionals to promote their participation in cancer screening programmes. This produced a non-significant result (OR; 95% CI=1.90; 0.87, 4.16). Subgroup analyses showed that age might be a heterogeneity factor (higher uptake in younger cohorts under 50 years (p=0.01)) and/or cancer type (higher in cervical than breast cancer screening programmes (p=0.01)).

#### Other Outcomes

Due to lack of evidence, no meta-analyses could be conducted for home visits on any other outcomes.

### Implementation Intentions

#### Screening Uptake

Four systematic reviews^48,50-52^ reported four estimates for the effect of including leaflets in screening material for patients to complete self-regulatory, goal-setting plans of ‘implementation intentions’ promoting screening. This showed no significant effect on screening uptake (OR; 95% CI=1.11; 0.75, 1.64). Subgroup analyses showed higher uptake in the most deprived compared to the least deprived area (p=<0.01).

#### Other Outcomes

Due to lack of evidence, no meta-analyses could be conducted for ‘implementation intentions’ on any other outcomes.

### Letters

#### Screening Uptake

Thirteen reviews^42-44,46-51,61-63,79^ provided altogether 38 estimates for the effect of letters demonstrating an overall increase in screening uptake (OR; 95% CI=1.51; 1.31, 1.75). There was heterogeneity surrounding the average effect, while the funnel plot contained a few ‘outliers’ suggesting potential presence of publication bias (see Supplementary file 9). In meta-analyses by letter type, the overall significance was retained for advance notification letters (prior to formal invitation or follow-up) distributed to patients due for screening (OR; 95% CI=1.13; 1.07, 1.20 – k=6 estimates in six reviews^42,47-51^). As was also the case for invitation letters signed by patients’ General Practitioners (GPs) (OR; 95% CI=1.35; 1.19, 1.53 – k=14 estimates across six reviews^42,44,47,48,50,51^). For GP-signed letters, the funnel plot had fewer ‘outliers’ vs. letters overall, but potential heterogeneity factors included: age; cancer type; context; as well as risk of bias. In all remaining meta-analyses, results were non-significant: invitation letters tailored to personal circumstances (OR; 95% CI=2.27; 0.81, 8.49 – k=4 in five reviews^42-44,46,47^); reminder letters following non-response to an invitation (OR; 95% CI=1.64 (0.77, 3.52) – k=5 across eight reviews^42,44,46,47,50,51,61,62^); as well as invitation letters with an emotive message targeting social norms and individuals’ responsibility of participating in screening (OR; 95% CI=1.00; 0.95, 1.05 – k=2 from one review^79^). In relation to socioeconomic status, reminder letters resulted in a higher uptake in the most deprived compared to the least deprived areas (p=0.03).

#### Other Outcomes

Due to lack of evidence, no meta-analyses could be conducted for letters on any other outcomes.

### Multi-Lingual Approaches

#### Screening Uptake

Multi-lingual approaches acknowledge that not all patients speak the majority language (e.g. English). As such, they attempt to reach patients by contacting them in languages they use, either through translating written material, over the phone, or speaking to patients face-to-face. Overall, regardless of format, these returned a significant positive effect (OR; 95% CI=1.35; 1.11, 1.63) with suspected heterogeneity and publication bias based on thirteen estimates from seven reviews.^42,44,46,51,74,80,83^ For multi-lingual delivery over the phone, uptake was higher in the most compared to the least deprived areas (p=<0.01).

#### Other Outcomes

Due to lack of evidence, no meta-analyses could be conducted for multi-lingual approaches on any other outcomes.

### Patient Navigation

#### Screening Uptake

For better navigation through patients’ screening pathways, there was only one estimate reported in one systematic review^64^ with a significant effect on uptake (OR; 95% CI=2.36; 1.99, 2.81).

#### Other Outcomes

Navigation of patients’ paths to lung cancer screening was assessed in one estimate reported in one review,^77^ suggesting shorter time (although non-significant) to their first consultation in the intervention compared with the control group (Supplementary file 8).

Whether patient navigation might also improve the chances of a patient being seen in follow-up consultations to assess and monitor lung cancer risks and symptoms, was considered in one estimate from one review^77^ and indicated a significant positive effect (Supplementary file 8).

### Phone Follow-Up

#### Screening Uptake

Phone follow-up of people overdue for screening consisted of overall 19 estimates from eleven reviews,^42-44,46-48,50,51,53,74,75^ with a significant impact on uptake (OR; 95% CI=1.58; 1.19, 2.09) but indication of heterogeneity and some funnel plot asymmetry. Seven reviews^42,44,46,47,50,51,74^ reported eleven estimates of follow-up specifically through phone calls, where a positive significant effect was retained (OR; 95% CI=1.49; 1.04, 2.14). Uptake was higher in the most compared to the least deprived areas (p=<0.01). For text message follow-up, five reviews^43,48,51,53,75^ reported seven estimates that also retained a positive effect (OR; 95% CI=1.28; 1.05, 1.56).

#### Other Outcomes

There was not enough evidence to conduct meta-analyses on other outcomes; however, in the case of text-message follow-up one estimate from one review^78^ showed a non-significant improvement of knowledge based on text-message follow-up of patients due for screening, while similarly for another estimate (k=1 from three reviews^57,58,78^) skin self-examination was not significantly impacted (Supplementary file 8).

### Simplified Methods

#### Screening Uptake

The distribution of simplified self-tests for other cancers (e.g. colorectal) may provide transferable lessons on the effect of simplifying screening procedures vs. standard procedures in general. Two systematic reviews^48,50^ reported on 13 such estimates with a significant increase in screening uptake (OR; 95% CI=1.26; 1.09, 1.46). There was higher uptake in people from ‘racial’ and ethnic groups that are underrepresented vs. that are part of the majority group (p=<0.05).

#### Other Outcomes

Due to lack of evidence, no meta-analyses could be conducted for simplified methods on any other outcomes.

### Survey

#### Screening Uptake

Two systematic reviews^48,51^ reported two estimates for distributing surveys to encourage patients to undertake screening, with no significant improvements (OR; 95% CI=0.96; 0.52, 1.74).

#### Other Outcomes

No meta-analyses could be conducted on other outcomes; however, the distribution of screening surveys failed to make a significant difference on screening intention (thus may not have reflected screening uptake as such) in one estimate from one review^52^ (Supplementary file 8).

### Targeting GPs

#### Screening Uptake

Finally, interventions that targeted GPs to increase uptake amongst their patient populations, regardless of method of incentivisation, indicated a significant increase in their patients’ screening uptake (OR; 95% CI=2.18; 1.13, 4.22), yet ostensible heterogeneity and publication bias (see Supplementary files 7 and 9), based on ten estimates from six reviews.^44,47,49-51,83^ Six of the estimates, reported in four reviews,^47,49-51^ considered provision of alerts to GPs when patients were overdue for screening, although losing significance (OR; 95% CI=2.04; 0.68, 6.13). Heterogeneity of individual estimates was possibly explained by study quality (p=<0.01).

#### Other Outcomes

Due to lack of evidence, no meta-analyses could be conducted for interventions targeting GPs on any other outcomes.

## Discussion

This is the first umbrella review, to our knowledge, which generated new meta-analyses across several cancers to identify effective interventions for screening participation and early detection of breast cancer and with an explicit focus on health inequalities. Even though this review focused on breast cancer, integrating transferable evidence from other common cancers increased the number of studies we could include in our meta-analyses. Our review, furthermore, differs from a standard umbrella review because we did not merely rely on already pooled average estimates, or re-analyse all individual studies from each included review. Instead, we selected the studies within the reviews that were relevant to high-income countries with universal healthcare coverage and organised screening programmes. In so doing, we included 43 systematic reviews and our meta-analyses of the relevant RCTs reported within these reviews showed significant increases in screening uptake for scheduled vs. open appointments, different types or contents of letters (advance notification, letters signed by GPs), educational information, phone follow-up calls or by text messages, counselling by phone, multi-lingual approaches overall. Available measures indicated higher uptake in the most deprived vs. the least deprived areas for multi-lingual approaches by phone, phone follow-up calls in general, reminder letters, as well as ‘implementation intentions’ (self-regulatory, goal-setting intervention). Other outcomes than screening uptake were less reported on, although we generated meta-analyses of relevant reported RCTs of interventions with significant improvements for knowledge scores (e.g. decision aids and educational information in different formats) and informed choice (decision aids) with potential influence on the prospect of earlier symptomatic presentation for at-risk patients. Overall, there was considerable statistical heterogeneity shown for most meta-analyses, potential publication bias and the majority of individual estimates were either of high or unclear risk of bias.

Many of the identified interventions are already implemented in a number of routine screening programmes. For example, the European Commission guidelines on breast screening recommends inviting women by letter with a decision aid explaining benefits and harms.^18^ Women in England are invited by the National Health Service’s Breast Screening Programme (NHSBSP) by letter offering them an appointment date and time together with an information leaflet, which is also available in several languages online.^85^ Having an explicit focus on inequalities throughout this umbrella review we have identified some additional promising interventions that could be used to reduce health inequalities in screening. However, there is a need to investigate further which of these interventions might work best in which circumstances and for whom. It is also recommended that wider prevention strategies (e.g. self-examination of symptoms) are integrated into routine screening programmes and related activities and supported as part of the infrastructure. A combination of interventions, therefore, might produce better results in practice.^48-51,63,74,77^ This then would beg the more complex question of which combinations can optimise effectiveness whilst also reducing inequalities. Involvement of affected and disadvantaged communities themselves seems here to be key, helping to both design and suggest amendments to current policies and practices that best represent their needs and priorities. Furthermore, future programmes need to take account of the extent to which they remain sustainable and cost-effective. Albeit, it is simultaneously hoped that many of the interventions suggested here (e.g. various letter types, phone contact) may be reproduced with relatively limited resources and can be built into pre-existing structures and practices without also requiring considerable investments in training of staff.^86^

Multi-lingual approaches are of particular relevance to populations of diverse backgrounds, especially people from underrepresented ethnic groups living in socially disadvantaged areas. Our analyses showed that especially multi-lingual delivery over the phone resulted in higher uptake in the most deprived areas. Language and culturally sensitive telephone reminders prior to screening appointments have been used successfully in the past when adapted to local circumstances in North East London, a socially disadvantaged area with a diverse population.^87^ Additionally, interventions that targeted GPs to increase uptake could also be implemented. This could include, for example, encouraging them to send an advance notification reminder letter signed by patients’ GPs and/or provide further educational materials on breast screening. Reports (e.g.^88^) also suggest that more advanced forms of invitation letters with GP signatures are successfully expanding across several National Health Service (NHS) practices.

### Comparison With Previous Literature

Comparing our findings with previous literature, 13 of our included reviews^43,47,49,50,63,65,67,70-72,74,77,83^ did not provide a meta-analysis themselves. As such, our inclusion criteria that reviews had to provide effect estimates, or data sufficient to calculate these, enabled us to additionally incorporate estimates from these reviews to contribute to our meta-analyses.

Thus, for screening, our meta-analyses of effective interventions support findings of reviews included in this study. However individually the reviews are comparatively limited in scope to certain cancer types or do not draw on a wider pool of available evidence by combining estimates from different reviews. For example, for advance notification letters we therefore extend the number of estimates from Goodwin’s review^48^ from four to six estimates by also incorporating estimates from Rat’s^51^ review. While for letters signed by GPs we combine and expand on meta-analyses or estimates previously considered for separate cancers (e.g. breast,^42^ cervical,^42^ colorectal^42,48,50^), as is also the case for scheduled versus open appointments (e.g. breast,^42^ cervical,^42,46^ colorectal^42^), as well as for the value of phone contact in various capacities including text messages as previously limited to colorectal cancer^53^ and counselling to breast and cervical^67^ and colorectal^48,50^ cancers, respectively. Furthermore, previous reviews of cervical cancer show the overall effectiveness of educational information in any format^44,46^– although not the particular effectiveness of educational information according to format (e.g. printed/paper-format for screening uptake) across a range of different cancer types (breast, colorectal, prostate and skin in addition to cervical), as has been discerned in the present umbrella review and meta-analyses. Finally, we have provided unique meta-analyses that, to our knowledge, have not previously been conducted, on multi-lingual approaches of specific relevance to non-native language-speaking and disadvantaged communities.

We acknowledge that there is relatively limited evidence identified on different interventions that promote awareness and help-seeking behaviours compared to interventions targeting screening uptake. However, this is also reflected in previous reviews. Supporting our wider synthesis of several relevant cancers, there is some evidence in the specific case of lung cancer^77,89^ and cervical cancer^76^ of the use of educational information to promote knowledge or awareness, in addition to the benefit for patients of decision aids in relation to breast,^68,70^ cervical,^70^ lung,^77^ prostate^66,68,69,72,73^ and colorectal^54,68-71^ cancers. Although our reviewed evidence consisted of single estimates not amenable to generate meta-analyses and therefore in need of further verification, additionally we observed the respective values of navigation for facilitating patients’ involvement in consultations as well as counselling to promote self-examination of symptoms.

### Recommendations for Research

Within the scope of the present umbrella review with meta-analyses, we would like to offer in particular four recommendations we believe can help improve future research.

Firstly, although some limited information was available for subgroup analyses including by area deprivation and ethnicity, these demographics were inconsistently reported across interventions. For socioeconomic inequalities, there were a few estimates available that might be transferable to breast cancer detection (multi-lingual approaches by phone, phone follow-up calls in general, reminder letters, as well as ‘implementation intentions’), while for ethnicity (Table 2) and the subgroup analyses referred to in Supplementary file 7 indicated that available estimates were mainly reported across interventions that might be less relevant to breast cancer such as standard invitation letters, choice of screening methods, and simplification of procedures. Furthermore, there was no information available on religion. Therefore, we recommend that more research consider and report on disadvantaged groups – a recommendation supported by the limited information on such groups identified by other reviews^46,47,50,60,69,70^ including a review concentrating on interventions to promote colorectal cancer screening amongst people from ‘racial’ and ethnic groups that are underrepresented with only one non-US study identified.^74^ Further supplementing the recommendation on providing data to enable inequality assessments, overall, this calls for more studies across and better reporting of key demographic characteristics.

Secondly, the relative volume of information on interventions to promote screening participation compared to on awareness and help-seeking behaviours, calls for more research into this latter category of interventions. However, we acknowledge that these interventions may be concerned with questions around issues of accessing GPs and mistrust of being taken seriously when presenting with symptoms that might not invariably be answered by an effectiveness review. Therefore, to complement our present review further exploration of the review evidence related to acceptability and barriers to symptomatic presentation might be needed.

Thirdly, and by extension, we recommended further research into relevant interventions in single payor contexts such as the UK. As an example, in our review only one estimate in one systematic review met inclusion criteria for patient navigation. This is one area (of potential others) where non-UK and in particular US studies may dominate. A 2022 review of patient navigation on population-based breast screening,^90^ for instance, included 15 studies but only one of these was non-US-based.

Finally, the relative volume of RCTs compared to non-RCTs may be due to the fact that systematic reviews have prioritised identification of this study design as associated with ‘best quality evidence’.^91^ Therefore, there may be a larger evidence base of non-RCTs to build into separate meta-analyses that may support, nuance or refute findings from the present review. Regardless of this, the majority of reported RCTs themselves had generally been assessed to be of either high or unclear risk of bias – so even for this ‘gold standard’ study design better quality evaluations would help build a stronger evidence base. We conducted sensitivity analyses of risk of bias. However, the reported RCTs predominantly had high or unclear risk of bias. As such, our sensitivity analyses could not be based on comparisons with lower risk of bias RCTs (but rather between high vs. unclear risk of bias). As reported across the reviews, the type of biases varied according to individual trials and interventions and the risk of bias assessment tools employed by the reviews (precluding direct comparisons of individual items reported). Nevertheless, biases often related to either: a lack of attention to or reporting of the randomisation sequence or allocation concealment; suspected deviations from intended interventions (not following the assigned study protocols); incomplete outcome data; selective reporting of outcomes; or a combination of these criteria. Future RCTs in the field therefore need to ensure that they fulfil as many of the required criteria as possible – not only when conducting the trials, but also that methods are explicitly and fully reported.

For systematic reviews, we ensured that only reviews that described included studies in adequate detail and used a satisfactory technique for assessing risk of bias in individual studies were included (see AMSTAR 2 items^34^). Therefore our conclusions are not based on information reported from lower quality (‘critically low’) reviews. These were excluded based on AMSTAR 2 assessments (i.e. with more than one critical flaw related to either protocol registration, literature search, justification for excluding studies and considering risk of bias when interpreting results). However, as the full-text exclusion reasons showed in Figure 1, there were as many as 49 reviews that did not use a satisfactory technique for assessing primary study bias, 30 that inadequately reported study details and 20 ‘critically low’ reviews. Although all AMSTAR 2 domains should be considered, future reviews in the field need, in particular, to account for the AMSTAR 2 domains that warranted exclusion from our review.

### Strengths and Limitations

This review has systematically integrated transferable evidence from other common cancers, in addition to breast cancer, to increase study numbers. It has also provided a broader overview of potentially promising interventions by building upon and extracting relevant information from individual reviews with more specific scopes than our umbrella review. However, a caveat needs to be highlighted that are applicable to umbrella reviews in general. Readers ought to be careful not to compare the relative effectiveness of multiple interventions as reported in this review in order to conclude based on the highest average pooled effect sizes which intervention is ‘best’. This is because the effect estimates stem from systematic reviews with varying scopes (including methodological and clinical differences in the respective interventions and corresponding primary study evaluations they report upon), which may induce ‘intransitivity’ and may not be confidently assessed solely through available information from the individual reviews alone (see Cochrane guidance^22^). By presenting interventions alphabetically, it is hoped that the impact of any comparisons of relative effectiveness that readers may make has been lessened compared to e.g. presenting the results in the order of the highest effects.

We have considered an extensive time period to identify relevant reviews and, as evidenced in Table 2, also captured a comprehensive overview of relatively up-to-date reviews in this field. However, another limitation is that further evidence might be available in unpublished literature which we did not consider, as well as in primary study sources that have not been captured by the reviews we included. The reviews, including their study search and selection, were of different quality (although ‘critically low’ reviews from our AMSTAR 2 assessments were excluded). Depending on the year of publication and when their searches were conducted, they may additionally not have captured more recent evidence. As such, we may not have retrieved the full range of more novel or technological interventions (e.g. as potentially reflected in the relatively few included interventions delivering educational information via the Internet). This time-delay factor is arguably a common limitation for umbrella reviews though.^22^ We also acknowledge the relatively strict criteria for comparable contexts by limiting to high-income countries with universal healthcare coverage. This means that our conclusions are based on these contexts and cannot be extrapolated to other settings, e.g. the US.

Finally, a word of caution for the subgroup/sensitivity analyses. This is especially as multiple outcomes were considered across several interventions. This resulted in a larger number of subgroup/sensitivity analyses and, correspondingly, p-values being considered. As such, it cannot be ruled out that for some of these results reported, they might be false ‘significant’ results.

## Conclusions

This umbrella review with meta-analyses has identified some effective interventions (i.e. scheduled vs. open appointments, different types of letters (advance notification, letters signed by GPs), educational information, phone follow-up calls or by text messages, counselling by phone, multi-lingual approaches). However, the relative availability of evidence on screening uptake compared to promotion of awareness and help-seeking behaviours suggests that particularly research into the latter category of interventions should be a priority. Further assessments should also be made across all outcomes of the extent to which interventions may reduce inequalities. This relates especially to ethnicity, religion and socioeconomic status as vital to explaining and reducing inequalities in breast cancer outcomes (but also other demographic characteristics).

## Supplemental Material

Supplemental Material - Improving Breast Cancer Outcomes by Enhanced Activities in Early Detection and Diagnosis: An Umbrella Review and Meta-Analyses of Randomised Controlled Trials in HNotigh-Income Contexts With Universal Healthcare Coverage Supplemental Material for Improving Breast Cancer Outcomes by Enhanced Activities in Early Detection and Diagnosis: An Umbrella Review and Meta-Analyses of Randomised Controlled Trials in High-Income Contexts With Universal Healthcare Coverage by Kristoffer Halvorsrud, Helen Fowler, Wasim Hamad, Helen A. Black, Suzanne E. Scott, Rosalind Raine, Elena Pizzo, Oleg Blyuss, Belinda Nedjai, Ranjit Manchanda, Jack Cuzick, Shuping J. Li, Fiona M. Walter, Stephen W. Duffy, and Judith Offman in Cancer Control.

## Data Availability

Data analysed during this study are included in this published article [and its Supplementary files].*
