# Supplementary material for: Improving Breast Cancer Outcomes by Enhanced Activities in Early Detection and Diagnosis: An Umbrella Review and Meta-Analyses of Randomised Controlled Trials in High-Income Contexts With Universal Healthcare Coverage
Source: Cancer Control. 2026 Jul 4;33:10732748261462921. doi: 10.1177/10732748261462921 (PMC13333060; doi:10.1177/10732748261462921)

PRISMA 2020 Checklist

Section and Topic	Item #	Checklist item	Location/page where item is reported
**TITLE**			
Title	1	Identify the report as a systematic review.	1
**ABSTRACT**			
Abstract	2	See the PRISMA 2020 for Abstracts checklist.	2-3
**INTRODUCTION**			
Rationale	3	Describe the rationale for the review in the context of existing knowledge.	3-5
Objectives	4	Provide an explicit statement of the objective(s) or question(s) the review addresses.	3-5
**METHODS**			
Eligibility criteria	5	Specify the inclusion and exclusion criteria for the review and how studies were grouped for the syntheses.	6-8
Information sources	6	Specify all databases, registers, websites, organisations, reference lists and other sources searched or consulted to identify studies. Specify the date when each source was last searched or consulted.	6
Search strategy	7	Present the full search strategies for all databases, registers and websites, including any filters and limits used.	Supplementary file 2
Selection process	8	Specify the methods used to decide whether a study met the inclusion criteria of the review, including how many reviewers screened each record and each report retrieved, whether they worked independently, and if applicable, details of automation tools used in the process.	6
Data collection process	9	Specify the methods used to collect data from reports, including how many reviewers collected data from each report, whether they worked independently, any processes for obtaining or confirming data from study investigators, and if applicable, details of automation tools used in the process.	9
Data items	10a	List and define all outcomes for which data were sought. Specify whether all results that were compatible with each outcome domain in each study were sought (e.g. for all measures, time points, analyses), and if not, the methods used to decide which results to collect.	8-11
	10b	List and define all other variables for which data were sought (e.g. participant and intervention characteristics, funding sources). Describe any assumptions made about any missing or unclear information.	9-11
Study risk of bias assessment	11	Specify the methods used to assess risk of bias in the included studies, including details of the tool(s) used, how many reviewers assessed each study and whether they worked independently, and if applicable, details of automation tools used in the process.	9
Effect measures	12	Specify for each outcome the effect measure(s) (e.g. risk ratio, mean difference) used in the synthesis or presentation of results.	10
Synthesis methods	13a	Describe the processes used to decide which studies were eligible for each synthesis (e.g. tabulating the study intervention characteristics and comparing against the planned groups for each synthesis (item #5)).	10-11
	13b	Describe any methods required to prepare the data for presentation or synthesis, such as handling of missing summary statistics, or data conversions.	10
	13c	Describe any methods used to tabulate or visually display results of individual studies and syntheses.	9-11
	13d	Describe any methods used to synthesize results and provide a rationale for the choice(s). If meta-analysis was performed, describe the model(s), method(s) to identify the presence and extent of statistical heterogeneity, and software package(s) used.	10-11
	13e	Describe any methods used to explore possible causes of heterogeneity among study results (e.g. subgroup analysis, meta-regression).	10-11
	13f	Describe any sensitivity analyses conducted to assess robustness of the synthesized results.	11
Reporting bias assessment	14	Describe any methods used to assess risk of bias due to missing results in a synthesis (arising from reporting biases).	9
Certainty	15	Describe any methods used to assess certainty (or confidence) in the body of evidence for an outcome.	10-11

PRISMA 2020 Checklist

Section and Topic	Item #	Checklist item	Location/page where item is reported
assessment			
**RESULTS**			
Study selection	16a	Describe the results of the search and selection process, from the number of records identified in the search to the number of studies included in the review, ideally using a flow diagram.	11-12, Figure 1
	16b	Cite studies that might appear to meet the inclusion criteria, but which were excluded, and explain why they were excluded.	Supplementary file 5
Study characteristics	17	Cite each included study and present its characteristics.	11-12, Table 1, Supplementary file 6
Risk of bias in studies	18	Present assessments of risk of bias for each included study.	Supplementary files 3 and 6
Results of individual studies	19	For all outcomes, present, for each study: (a) summary statistics for each group (where appropriate) and (b) an effect estimate and its precision (e.g. confidence/credible interval), ideally using structured tables or plots.	Table 1, Supplementary file 6
Results of syntheses	20a	For each synthesis, briefly summarise the characteristics and risk of bias among contributing studies.	Results section, Supplementary files 7 and 8
	20b	Present results of all statistical syntheses conducted. If meta-analysis was done, present for each the summary estimate and its precision (e.g. confidence/credible interval) and measures of statistical heterogeneity. If comparing groups, describe the direction of the effect.	Results section, Figures 2 and 3, Table 3, Supplementary files 7 and 8
	20c	Present results of all investigations of possible causes of heterogeneity among study results.	Table 3, Supplementary files 7 and 8
	20d	Present results of all sensitivity analyses conducted to assess the robustness of the synthesized results.	Supplementary files 7 and 8
Reporting biases	21	Present assessments of risk of bias due to missing results (arising from reporting biases) for each synthesis assessed.	Supplementary files 7 and 8
Certainty of evidence	22	Present assessments of certainty (or confidence) in the body of evidence for each outcome assessed.	Results section, Supplementary files 7, 8 and 9
**DISCUSSION**			
Discussion	23a	Provide a general interpretation of the results in the context of other evidence.	22,23,25,26
	23b	Discuss any limitations of the evidence included in the review.	28-30
	23c	Discuss any limitations of the review processes used.	28-30

Section and Topic	Item #	Checklist item	Location/page where item is reported
	23d	Discuss implications of the results for practice, policy, and future research.	22-25, 26-28
**OTHER INFORMATION**			
Registration and protocol	24a	Provide registration information for the review, including register name and registration number, or state that the review was not registered.	Title page
	24b	Indicate where the review protocol can be accessed, or state that a protocol was not prepared.	Title page
	24c	Describe and explain any amendments to information provided at registration or in the protocol.	N/A
Support	25	Describe sources of financial or non-financial support for the review, and the role of the funders or sponsors in the review.	Title page
Competing interests	26	Declare any competing interests of review authors.	Title page
Availability of data, code and other materials	27	Report which of the following are publicly available and where they can be found: template data collection forms; data extracted from included studies; data used for all analyses; analytic code; any other materials used in the review.	Title page

From: Page MJ, McKenzie JE, Bossuyt PM, Boutron I, Hoffmann TC, Mulrow CD, et al. The PRISMA 2020 statement: an updated guideline for reporting systematic reviews. BMJ 2021;372:n71. doi: 10.1136/bmj.n71. This work is licensed under CC BY 4.0. To view a copy of this license, visit https://creativecommons.org/licenses/by/4.0/

Ovid MEDLINE

1. exp "Breast Neoplasms"/ or exp "Melanoma"/ or exp "Skin Neoplasms"/ or exp "Prostatic Neoplasms"/ or exp "Lung Neoplasms"/ or exp "Colorectal Neoplasms"/ or exp "Uterine Neoplasms"/ or exp "Head and Neck Neoplasms"/
2. ((cancer* or neoplasm* or tumor* or tumour* or cyst* or carcinoma* or malignan*) adj3 (breast* or chest* or skin or melanom* or prostat* or lung* or bowel* or colorectal or uterus or uterine or womb or head or neck or "upper aerodigestive tract" or cervical or cervix)).ti,ab.
3. 1 or 2
4. "Mass Screening"/ or "Anonymous Testing"/ or "Mass Chest X-Ray"/ or "Multiphasic Screening"/ or exp Mammography/ or "Reminder Systems"/
5. ((screen* or mammogra* or "xero mammogra*" or xeromammogra* or "xero radiogra*" or xeroradiogra* or "x ray*" or xray* or imaging or radiogra* or "ultra sound*" or ultrasound* or "ultra sonogra*" or ultrasonogra* or sonogra* or echocardiogra* or scan*) adj3 (intervention* or program* or campaign* or support* or guid* or advice or advis* or inform* or triag* or direct* or signpost* or reminder* or alert* or messag* or letter* or sms or mms or telephone or phone or call* or mail* or email* or incentive* or "re imburs*" or reimburs* or voucher* or token* or reward* or invitation* or appointment*)).ti,ab.
6. 4 or 5
7. "Healthcare Disparities"/ or "Patient Navigation"/ or "Early Detection of Cancer"/ or "Patient Participation"/ or "Patient Compliance"/ or exp "Patient Acceptance of Health Care"/ or exp "Health Services Accessibility"/
8. (participation or involvement or complian* or attend* or accept* or access* or use* or uptake or utiliz* or utilis* or barrier* or hindrance* or obstacle* or challenge* or facilitat* or inequal* or unequal or disparat*).ti,ab.
9. 7 or 8

10. "Health Communication"/ or "Persuasive Communication"/ or "Health Education"/ or exp "Consumer Health Information"/ or "Patient Education as Topic"/ or "Teach-Back Communication"/ or "Health Fairs"/ or exp "Health Promotion"/ or "Delivery of Health Care"/ or exp "After-Hours Care"/ or "Culturally Competent Care"/ or "Self-Testing"/
11. ((health or healthcare or "health care" or "health fair" or system or service* or community or population or public or media or educat* or literacy or communication* or information* or behaviour* or behavior*) adj3 (promot* or campaign* or initiative* or strateg* or intervention* or program*)).ti,ab.
12. (cultur* adj3 (sensitiv* or adapt* or appropriat* or competen*)).ti,ab.
13. ("help line*" or helpline* or "hot line*" or hotline* or "out of hour*" or "extended hour*" or "after hour*" or "walk in" or "drop in" or "self sampl*" or selfsampl* or "self test*" or selftest* or "self collect*" or selfcollect* or "home sampl*" or homesampl* or "home test*" or hometest* or "home collect*" or homecollect*).ti,ab.
14. 10 or 11 or 12 or 13
15. "Healthcare Disparities"/ or "Patient Navigation"/ or "Early Detection of Cancer"/ or "Information Seeking Behavior"/ or "Self Care"/ or "Patient Participation"/ or "Patient Compliance"/ or exp "Patient Acceptance of Health Care"/ or exp "Health Services Accessibility"/ or "Health Knowledge, Attitudes, Practice"/ or "Attitude to Health"/ or "Health Behavior"/ or "Health Risk Behaviors"/ or "Risk Reduction Behavior"/ or exp "Self-Examination"/
16. ((service* or care or healthcare or patient*) adj3 (pathway* or navigation)).ti,ab.
17. (helpseek* or behaviour* or behavior* or belief* or expectation* or aware* or knowledge* or attitude* or understand*).ti,ab.
18. ((help or information*) adj3 seek*).ti,ab.
19. ((seek* or sought or diagnos* or present* or detect* or attend* or consult* or refer* or patient*) adj3 (delay* or late or later or early or earlier or postpone* or wait* or deny or denial or time or interval* or inequal* or unequal or disparat*)).ti,ab.
20. (symptom* adj3 (present* or diagnos* or apprais* or detect* or recogni* or duration or onset*)).ti,ab.
21. 15 or 16 or 17 or 18 or 19 or 20
22. 3 and 6 and 9
23. 3 and 14 and 21
24. 22 or 23
25. MEDLINE.tw.
26. systematic review.tw.
27. meta analysis.pt.
28. 25 or 26 or 27
29. 24 and 28
30. limit 29 to (english language and humans)
31. remove duplicates from 30
32. limit 31 to dt=19460101-20251231

Ovid EMBASE

1. exp "Breast Tumor"/ or exp "Melanoma"/ or exp "Skin Tumor"/ or exp "Prostate Tumor"/ or exp "Lung Tumor"/ or exp "Colorectal Tumor"/ or exp "Uterus Tumor"/ or exp "Head and Neck Tumor"/
2. ((cancer* or neoplasm* or tumor* or tumour* or cyst* or carcinoma* or malignan*) adj3 (breast* or chest* or skin or melanom* or prostat* or lung* or bowel* or colorectal or uterus or uterine or womb or head or neck or "upper aerodigestive tract" or cervical or cervix)).ti,ab.
3. 1 or 2
4. "Mass Screening"/ or "Cancer Screening"/ or "Anonymous Testing"/ or exp "Thorax Radiography"/ or "Multiphasic Screening"/ or exp "Breast Examination"/ or "Reminder System"/
5. ((screen* or mammogra* or "xero mammogra*" or xeromammogra* or "xero radiogra*" or xeroradiogra* or "x ray*" or xray* or imaging or radiogra* or "ultra sound*" or ultrasound* or "ultra sonogra*" or ultrasonogra* or sonogra* or echocardiogra* or scan*) adj3 (intervention* or program* or campaign* or support* or guid* or advice or advis* or inform* or triag* or direct* or signpost* or reminder* or alert* or messag* or letter* or sms or mms or telephone or phone or call* or mail* or email* or incentive* or "re imburs*" or reimburs* or voucher* or token* or reward* or invitation* or appointment*)).ti,ab.
6. 4 or 5
7. "Health Care Disparity"/ or "Patient Care"/ or "Early Cancer Diagnosis"/ or "Patient Participation"/ or "Patient Compliance"/ or "Patient Engagement"/ or "Patient Attendance"/ or "Health Care Access"/
8. (participation or involvement or complian* or attend* or accept* or access* or use* or uptake or utiliz* or utilis* or barrier* or hindrance* or obstacle* or challenge* or facilitat* or inequal* or unequal or disparat*).ti,ab.
9. 7 or 8
10. "Medical Information"/ or "Persuasive Communication"/ or "Health Education"/ or exp "Health Literacy"/ or "Patient Education"/ or "Consumer Health Information"/ or "Interpersonal Communication"/ or exp "Health Promotion"/ or "Health Care Delivery"/ or "Out-of-hours Care"/ or "Transcultural Care"/ or "Self-Testing"/
11. ((health or healthcare or "health care" or "health fair" or system or service* or community or population or public or media or educat* or literacy or communication* or information* or behaviour* or behavior*) adj3 (promot* or campaign* or initiative* or strateg* or intervention* or program*)).ti,ab.

12. (cultur* adj3 (sensitiv* or adapt* or appropriat* or competen*)).ti,ab.
13. ("help line*" or helpline* or "hot line*" or hotline* or "out of hour*" or "extended hour*" or "after hour*" or "walk in" or "drop in" or "self sampl*" or selfsampl* or "self test*" or selftest* or "self collect*" or selfcollect* or "home sampl*" or homesampl* or "home test*" or hometest* or "home collect*" or homecollect*).ti,ab.
14. 10 or 11 or 12 or 13
15. "Health Care Disparity"/ or "Patient Care"/ or "Early Cancer Diagnosis"/ or "Information Seeking"/ or "Self Care"/ or "Self Care Agency"/ or "Self Help"/ or "Patient Participation"/ or "Patient Compliance"/ or "Patient Attitude"/ or "Health Care Access"/ or "Attitude to Health"/ or "Knowledge"/ or "Health Behavior"/ or "High Risk Behavior"/ or "Risk Reduction"/ or exp "Self Examination"/
16. ((service* or care or healthcare or patient*) adj3 (pathway* or navigation)).ti,ab.
17. (helpseek* or behaviour* or behavior* or belief* or expectation* or aware* or knowledge* or attitude* or understand*).ti,ab.

18. ((help or information*) adj3 seek*).ti,ab.
19. ((seek* or sought or diagnos* or present* or detect* or attend* or consult* or refer* or patient*) adj3 (delay* or late or later or early or earlier or postpone* or wait* or deny or denial or time or interval* or inequal* or unequal or disparat*)).ti,ab.
20. (symptom* adj3 (present* or diagnos* or apprais* or detect* or recogni* or duration or onset*)).ti,ab.
21. 15 or 16 or 17 or 18 or 19 or 20
22. 3 and 6 and 9
23. 3 and 14 and 21
24. 22 or 23
25. systematic review.tw.
26. meta-analysis.tw.
27. 25 or 26
28. 24 and 27
29. limit 28 to (english language and humans)
30. remove duplicates from 29
31. limit 30 to dc=19800101-20251231

Cochrane Database of Systematic Reviews

#1 MeSH descriptor: [Breast Neoplasms] explode all trees
#2 MeSH descriptor: [Melanoma] explode all trees
#3 MeSH descriptor: [Skin Neoplasms] explode all trees
#4 MeSH descriptor: [Prostatic Neoplasms] explode all trees
#5 MeSH descriptor: [Lung Neoplasms] explode all trees
#6 MeSH descriptor: [Colorectal Neoplasms] explode all trees
#7 MeSH descriptor: [Uterine Neoplasms] explode all trees
#8 MeSH descriptor: [Head and Neck Neoplasms] explode all trees
#9 ((cancer* OR neoplasm* OR tumor* OR tumour* OR cyst* OR carcinoma* OR malignan*) near/3 (breast* OR chest* OR skin OR melanom* OR prostat* OR lung* OR bowel* OR colorectal OR uterus OR uterine OR womb OR head OR neck OR "upper aerodigestive tract" or cervical or cervix)):ti,ab,kw (Word variations have been searched)
#10 #1 or #2 or #3 or #4 or #5 or #6 or #7 or #8 or #9
#11 MeSH descriptor: [Mass Screening] this term only
#12 MeSH descriptor: [Anonymous Testing] this term only
#13 MeSH descriptor: [Mass Chest X-Ray] this term only
#14 MeSH descriptor: [Multiphasic Screening] this term only
#15 MeSH descriptor: [Mammography] explode all trees
#16 MeSH descriptor: [Reminder Systems] this term only

#17 ((screen* OR mammogra* OR "xero mammogra*" OR xeromammogra* OR "xero radiogra*" OR xeroradiogra* OR "x ray*" OR xray* OR imaging OR radiogra* OR "ultra sound*" OR ultrasound* OR "ultra sonogra*" OR ultrasonogra* OR sonogra* OR echocardiogra* OR scan*) near/3 (intervention* OR program* OR campaign* OR support* OR guid* OR advice OR advis* OR inform* OR triag* OR direct* OR signpost* OR reminder* OR alert* OR messag* OR letter* OR sms OR mms OR telephone OR phone OR call* OR mail* OR email* OR incentive* OR "re imburs*" OR reimburs* OR voucher* OR token* OR reward* OR invitation* OR appointment*)):ti,ab,kw (Word variations have been searched)
#18 #11 or #12 or #13 or #14 or #15 or #16 or #17
#19 MeSH descriptor: [Healthcare Disparities] this term only
#20 MeSH descriptor: [Patient Navigation] this term only
#21 MeSH descriptor: [Early Detection of Cancer] this term only
#22 MeSH descriptor: [Patient Participation] this term only
#23 MeSH descriptor: [Patient Compliance] this term only
#24 MeSH descriptor: [Patient Acceptance of Health Care] explode all trees
#25 MeSH descriptor: [Health Services Accessibility] explode all trees
#26 (participation OR involvement OR complian* OR attend* OR accept* OR access* OR use* OR uptake OR utiliz* OR utilis* OR barrier* OR hindrance* OR obstacle* OR challenge* OR facilitat* OR inequal* OR unequal OR disparat*):ti,ab,kw (Word variations have been searched)
#27 #19 or #20 or #21 or #22 or #23 or #24 or #25 or #26
#28 MeSH descriptor: [Health Communication] this term only
#29 MeSH descriptor: [Persuasive Communication] this term only
#30 MeSH descriptor: [Health Education] this term only
#31 MeSH descriptor: [Consumer Health Information] explode all trees
#32 MeSH descriptor: [Patient Education as Topic] this term only
#33 MeSH descriptor: [Teach-Back Communication] this term only
#34 MeSH descriptor: [Health Fairs] this term only
#35 MeSH descriptor: [Health Promotion] explode all trees
#36 MeSH descriptor: [Delivery of Health Care] this term only
#37 MeSH descriptor: [After-Hours Care] explode all trees
#38 MeSH descriptor: [Culturally Competent Care] this term only
#39 MeSH descriptor: [Self-Testing] this term only
#40 ((health OR healthcare OR "health care" OR "health fair" OR system OR service* OR community OR population OR public OR media OR educat* OR literacy OR communication* OR information* OR behaviour* OR behavior*) near/3 (promot* OR campaign* OR initiative* OR strateg* OR intervention* OR program*)):ti,ab,kw (Word variations have been searched)
#41 ((cultur*) near/3 (sensitiv* OR adapt* OR appropriat* OR competen*)):ti,ab,kw (Word variations have been searched)
#42 ("help line*" OR helpline* OR "hot line*" OR hotline* OR "out of hour*" OR "extended hour*" OR "after hour*" OR "walk in" OR "drop in" OR "self sampl*" OR selfsampl* OR "self test*" OR selftest* OR "self collect*" OR selfcollect* OR "home sampl*" OR homesampl* OR "home test*" OR hometest* OR "home collect*" OR homecollect*):ti,ab,kw (Word variations have been searched)
#43 #28 or #29 or #30 or #31 or #32 or #33 or #34 or #35 or #36 or #37 or #38 or #39 or #40 or #41 or #42
#44 MeSH descriptor: [Healthcare Disparities] this term only

#45 MeSH descriptor: [Patient Navigation] this term only
#46 MeSH descriptor: [Early Detection of Cancer] this term only
#47 MeSH descriptor: [Information Seeking Behavior] this term only
#48 MeSH descriptor: [Self Care] this term only
#49 MeSH descriptor: [Patient Participation] this term only
#50 MeSH descriptor: [Patient Compliance] this term only
#51 MeSH descriptor: [Patient Acceptance of Health Care] explode all trees
#52 MeSH descriptor: [Health Services Accessibility] explode all trees
#53 MeSH descriptor: [Health Knowledge, Attitudes, Practice] this term only
#54 MeSH descriptor: [Attitude to Health] this term only
#55 MeSH descriptor: [Health Behavior] this term only
#56 MeSH descriptor: [Health Risk Behaviors] this term only
#57 MeSH descriptor: [Self-Examination] explode all trees
#58 MeSH descriptor: [Risk Reduction Behavior] this term only
#59 ((service* or care or healthcare or patient*) near/3 (pathway* or navigation)):ti,ab,kw (Word variations have been searched)
#60 (helpseek* or behaviour* or behavior* or belief* or expectation* or aware* or knowledge* or attitude* or understand*):ti,ab,kw (Word variations have been searched)
#61 ((help OR information*) near/3 (seek*)):ti,ab,kw (Word variations have been searched)
#62 ((seek* OR sought OR diagnos* OR present* OR detect* OR attend* OR consult* OR refer* OR patient*) near/3 (delay* OR late OR later OR early OR earlier OR postpone* OR wait* OR deny OR denial OR time OR interval* OR inequal* OR unequal OR disparat*)):ti,ab,kw (Word variations have been searched)
#63 ((symptom*) near/3 (present* OR diagnos* OR apprais* OR detect* OR recogni* OR duration OR onset*)):ti,ab,kw (Word variations have been searched)
#64 #44 or #45 or #46 or #47 or #48 or #49 or #50 or #51 or #52 or #53 or #54 or #55 or #56 or #57 or #58 or #59 or #60 or #61 or #62 or #63
#65 #10 and #18 and #27
#66 #10 and #43 and #64
#67 #65 or #66

Reference	Did the research questions and inclusion criteria for the review include the components of PICO?	Did the report of the review contain an explicit statement that the review methods were	Did the review authors explain their selection of the study designs for	Did the review authors use a comprehensive literature search strategy?	Did the review authors perform study selection in duplicate?	Did the review authors perform data extraction in duplicate?	Did the review authors provide a list of excluded studies and justify the exclusions?	Did the review authors report on the sources of funding for the studies	If meta-analysis was performed did the review authors use appropriate methods for statistical combination of results?	If meta-analysis was performed, did the	Did the review authors account for RoB in individual studies when	Did the review authors provide a satisfactor	If they performed quantitative synthesi	Did the review authors report any

PRISMA 2020 Checklist

	established prior to the conduct of the review and did the report justify any significant deviations from the protocol?	inclusion in the review?		included in the review?	review authors assess the potential impact of RoB in individual studies on the results of the meta-analysis or other evidence synthesis?	interpreting/discussing the results of the review?	y explanation for, and discussion of, any heterogeneity observed in the results of the review?	s did the review authors carry out an adequate investigation of publication bias (small study bias) and discuss its likely impact on the results of the review?	potential sources of conflict of interest, including any funding they received for conducting the review?

PRISMA 2020 Checklist

Abdul Latip et al 2022	No	Partial Yes	No	Partial Yes	Yes	No	No	No	No meta-analysis conducted	No meta-analysis conducted	Yes	No	No meta-analysis conducted	Yes
Ahadinezhad et al 2024	Yes	No	No	Partial Yes	No	Yes	Partial Yes	No	Yes	Yes	Yes	Yes	Yes	Yes
Alam et al 2023	Yes	Yes	Yes	Partial Yes	Yes	Yes	No	No	Yes	Yes	Yes	Yes	Yes	Yes
Ampofo et al 2022	Yes	Yes	Yes	Partial Yes	Yes	No	No	No	Yes	No	Yes	Yes	Yes	Yes
Baptista et al 2018	Yes	No	No	Partial Yes	Yes	Yes	Yes	No	Yes	Yes	Yes	No	No	Yes
Brouwers et al 2011	Yes	Partial Yes	Yes	Partial Yes	No	No	No	Yes	No meta-analysis conducted	No meta-analysis conducted	Yes	No	No meta-analysis conducted	Yes
Camilloni et al 2013	Yes	No	No	Partial Yes	No	No	Yes	Yes	Yes	No	Yes	No	No	Yes
Chambergo-Michilot et al 2020	Yes	Partial Yes	No	Partial Yes	Yes	Yes	Yes	Yes	Yes	No	Yes	Yes	No	No
Chan and So 2021	Yes	Yes	No	Partial Yes	Yes	No	No	No	No meta-analysis conducted	No meta-analysis conducted	Yes	No	No meta-analysis conducted	Yes
Costa et al 2022	Yes	Partial Yes	No	Partial Yes	Yes	No	No	No	Yes	No	Yes	Yes	Yes	Yes
Dawkins et al 2025	Yes	Yes	Yes	Partial Yes	Yes	No	No	No	Yes	Yes	Yes	Yes	Yes	Yes

Dieng et al 2014	Yes	Partial Yes	No	Partial Yes	No	No	No	No	Yes	No	Yes	No	No	No
Edwards et al 2013	Yes	Yes	Yes	Partial Yes	Yes	Yes	Yes	Yes	Yes	Yes	Yes	Yes	Yes	Yes
Elepaño et al 2021	Yes	Partial Yes	No	Partial Yes	Yes	Yes	No	No	Yes	No	Yes	Yes	No	Yes
Ersser et al 2019	Yes	Partial Yes	No	Partial Yes	Yes	Yes	No	No	No	No	Yes	Yes	No	Yes
Goodwin et al 2019	Yes	Yes	No	Partial Yes	Yes	No	No	No	No	Yes	Yes	Yes	Yes	Yes
Henrikson et al 2018	Yes	No	No	Partial Yes	Yes	Yes	Yes	No	Yes	No	Yes	No	No	Yes
Ilic et al 2015	Yes	No	No	Partial Yes	Yes	Yes	Yes	No	No meta-analysis conducted	No meta-analysis conducted	Yes	Yes	No meta-analysis conducted	Yes
Issaka et al 2019	Yes	Partial Yes	No	Partial Yes	Yes	Yes	No	No	No meta-analysis conducted	No meta-analysis conducted	Yes	No	No meta-analysis conducted	Yes
Ivlev et al 2018	Yes	Yes	No	Partial Yes	Yes	Yes	Yes	No	Yes	Yes	Yes	No	Yes	Yes
Jepson et al 2001	Yes	Partial Yes	No	Yes	No	No	No	No	No meta-analysis conducted	No meta-analysis conducted	Yes	No	No meta-analysis conducted	Yes
Larsen et al 2021	Yes	Yes	No	Partial Yes	Yes	Yes	Yes	No	No	No	Yes	Yes	Yes	Yes
Lau et al	Yes	Partial Yes	No	Partial Yes	Yes	No	No	No	Yes	No	Yes	Yes	Yes	Yes

2022														
Li et al 2020	Yes	Partial Yes	No	Partial Yes	Yes	Yes	No	No	Yes	No	Yes	Yes	No	Yes
Liu et al 2024	Yes	Yes	Yes	Partial Yes	Yes	Yes	No	No	Yes	Yes	Yes	Yes	Yes	Yes
Martínez-González et al 2018	Yes	Partial Yes	No	Partial Yes	Yes	No	No	Yes	Yes	No	Yes	Yes	No	Yes
McAlpine et al 2018	No	Partial Yes	No	Partial Yes	Yes	Yes	Yes	No	Yes	No	Yes	Yes	No	Yes
Musa et al 2017	Yes	Yes	No	Partial Yes	Yes	No	Partial Yes	Yes	Yes	No	Yes	No	Yes	Yes
Ramli et al 2021	No	Yes	Yes	Partial Yes	Yes	No	No	Yes	Yes	Yes	Yes	Yes	Yes	Yes
Rat et al 2018	Yes	Partial Yes	Yes	Partial Yes	Yes	No	No	No	No meta-analysis conducted	No meta-analysis conducted	Yes	No	No meta-analysis conducted	Yes
Riganti et al 2024	Yes	Yes	Yes	Partial Yes	Yes	Yes	Yes	Yes	Yes	Yes	Yes	Yes	Yes	Yes
Riikonen et al 2019	Yes	Partial Yes	Yes	Partial Yes	Yes	Yes	Yes	No	Yes	No	Yes	No	No	Yes
Rodriguez-Gomez et al 2020	Yes	Partial Yes	Yes	Partial Yes	Yes	Yes	No	No	No meta-analysis conducted	No meta-analysis conducted	Yes	Yes	No meta-analysis conducted	Yes
Ruco et al 2021	Yes	Yes	Yes	Partial Yes	Yes	Yes	No	Yes	Yes	Yes	Yes	Yes	Yes	Yes
Saab et al 2021	Yes	Partial Yes	Yes	Partial Yes	Yes	Yes	No	No	No meta-analysis conducted	No meta-analysis cond	Yes	Yes	No meta-analysis cond	No

PRISMA 2020 Checklist

										ucted			ucted	
Schliemann et al 2019	Yes	Partial Yes	No	Partial Yes	Yes	Yes	No	Yes	No meta-analysis conducted	No meta-analysis conducted	Yes	No	No meta-analysis conducted	Yes
Stacey et al 2024	Yes	Yes	No	Partial Yes	No	Yes	Yes	No	Yes	Yes	Yes	No	Yes	Yes
Staley et al 2021	Yes	Yes	Yes	Yes	Yes	Yes	Yes	No	Yes	Yes	Yes	Yes	Yes	Yes
Teo et al 2018	Yes	Partial Yes	Yes	Partial Yes	Yes	Yes	No	No	Yes	Yes	Yes	No	No	Yes
Tsipa et al 2021	Yes	Partial Yes	Yes	Partial Yes	Yes	Yes	No	No	Yes	Yes	Yes	Yes	Yes	Yes
van Agt et al 2017	No	Partial Yes	No	Partial Yes	Yes	Yes	Yes	No	No meta-analysis conducted	No meta-analysis conducted	Yes	No	No meta-analysis conducted	Yes
Verbunt et al 2024	Yes	Partial Yes	No	Partial Yes	Yes	Yes	No	No	No meta-analysis conducted	No meta-analysis conducted	Yes	Yes	No meta-analysis conducted	Yes
Verdoodt et al 2015	Yes	Partial Yes	Yes	Partial Yes	Yes	Yes	Yes	No	Yes	No	Yes	No	No	Yes

PRISMA 2020 Checklist

References (reviews horizontal, primary studies vertical)	Abdul Latip et al 2022	Ahadinezhad et al 2024	Alam et al 2023	Ampofo et al 2022	Baptista et al 2018	Brouwers et al 2011	Camilloni et al 2013	Chamberrgo-Michilot et al 2020	Chan and So 2021	Costa et al 2022	Dawkins et al 2025	Dieng et al 2014	Edwards et al 2013	Elepaño et al 2021	Ersser et al 2019	Goodwin et al 2019	Henrikson et al 2018	Ilic et al 2015	Issaka et al 2019	Ivlev et al 2018	Jepson et al 2001	Larsen et al 2021	Lau et al 2022	Li et al 2020	Liu et al 2024	Martínez-González et al 2018	McAlpine et al 2018	Musa et al 2017	Ramli et al 2021	Rat et al 2018	Riganti et al 2024	Riikonen et al 2019	Rodriguez-Gomez et al 2020	Ruco et al 2021	Saab et al 2021	Schliemann et al 2019	Stacey et al 2024	Staley et al 2021	Teo et al 2018	Tsipa et al 2021	van Agt et al 2017	Verbunt et al 2024	Verdoodt et al 2015
Acera 2017																																						X					
Albada 2012												X																															
Arcas et al 2014																																		X									
Aubin-Auger et al 2014																														X												X	
Bais 2007										X																																	X
Barthe et al 2015																																								X			
Bartholomew et al 2019																													X														
Baxter and Barat				X																																							

PRISMA 2020 Checklist

a 2011																																											
Beti Thompson 2017																									X																		
Birkenfeld et al 2011																														X													
Boguradzka et al 2014																																								X			
Bowman 1995							X																															X					
Brain 2000												X																															
Braithwaite 2005												X																															
Brewer 2021										X																																	
Broberg 2013																																						X					
Broc et al 2015									X																																		
Buehler et al 1997																												X										X					
Cadman										X																																	X

PRISMA 2020 Checklist

2015																																											
Campbell 1997													X																														
Clouston et al 2014		X																					X																	X			
Clover et al 1996																																	X										
Cole et al 2003																X														X													
Cole et al 2007						X	X									X			X											X										X			
Darlin 2013										X																																	X
Davidson et al 1999																					X											X											
Decker et al 2013																												X										X					
Del Mar 1998																																						X					
Denters et al 2013																X																											
Deutekom et al 2010																X																											

PRISMA 2020 Checklist

Dodd 2019																																										X	
Dubey et al 2006						X																																					
Eaker 2004							X																					X										X					
Elfström 2019										X																																	
Emery et al 2019																																			X								
Enerly 2016										X																		X															
Evans 2010					X													X		X							X					X					X		X				
Federici et al 2005																														X													
Federici et al 2006						X																																					
Firmino-Machado et al 2019																									X									X									
Fry 2003												X																															
Fujiwara et al																												X															

PRISMA 2020 Checklist

2015																																											
Gabel et al 2020		X																				X	X																				
Gattellari 2003																		X		X						X	X										X		X				
Gattellari 2005																		X		X							X										X		X				
Gimeno-Garcia et al 2009						X	X																							X										X			
Giorgi 2000							X																																				
Giorgi Rossi 2015										X																		X															X
Glazebrook et al 2006												X	X				X																										
Gold 2011								X																																			
Guiriguet et al 2016																			X																					X		X	
Gummersbach 2015																															X												
Hago																																		X		X				X			

PRISMA 2020 Checklist

el et al 2016																																											
Haguenoer 2015										X																		X										X					X
Hegenscheid 2011							X																																				
Heranney 2011							X																					X										X					
Heverin 2011																																							X				
Hewitson 2011							X									X														X										X			
Hirst et al 2017														X		X																		X									
Hoare 1994							X																																				
Hol et al 2010																														X													
Hong 2014																																				X			X				
Hou et al 2005						X																														X							
Huf et al 2020																									X									X									
Hughes et																X														X													

PRISMA 2020 Checklist

al 2005																																											
Hunt 1998																																						X					
Ilic 2008					X															X																			X				
Ingrand et al 2016																								X																			
Ishikawa et al 2012																																				X							
Jalili 2019										X																																	
Janda 2014																																							X				
Janda et al 2011															X		X																						X				
Jensen et al 2009						X																																X					
Kellen 2018										X																																	
Kerrison et al 2015																																		X									
King et al 1994																X																											
Kitchener 2017										X															X																		
Kregt																														X													

PRISMA 2020 Checklist

ing 2020																																											
Lancaster 1992																																						X					
Levi et al 2011																														X													
Libby et al 2011																X																											
Lilliecreutz 2020										X																																	
Lo et al 2014		X														X																											
Lonnberg 2016																																						X					
Mac Donald 2021										X																																	
MACS Group 2006																														X													
Mant 1992							X																							X													
Mathieu 2007																											X				X						X				X		
Mathieu 2010																											X				X						X				X		
McAvoy							X																															X					

PRISMA 2020 Checklist

1991																																											
McCaffery 2010																											X																
McDowell 1989																																						X					
Moen 2020			X																																							X	
Morrell et al 2005						X	X																					X										X					
Moss et al 2017																X																											
Mullins 2009																																						X					
Nadarzynski et al 2012				X																																							
Neter et al 2014		X														X														X										X			
Ng et al 1998																																				X							
Nichols 1986							X																																				
O'Carroll et al 2015		X														X																								X			
O'Co							X																																				

PRISMA 2020 Checklist

nnor 1998																																											
Page et al 2006						X	X																																				
Perestelo-Perez et al 2019																																					X						
Pérez-Lacasta 2019																															X												
Piana 2011							X			X																																	X
Pierce 1989																																						X					
Pignone 2013																																							X				
Pritchard 1995							X																															X					
Qureshi 2021			X																																								
Racey 2016										X																		X					X					X					
Radde et al 2016																												X										X					
Raine et al 2016																																								X			

PRISMA 2020 Checklist

Rat et al 2014																	X																										
Rat et al 2017																			X																							X	
Reder 2017																															X												
Richardson 1994							X																																				
Ritvo et al 2015																								X																			
Roberto 2020																															X						X						
Robinson et al 1994																X																											
Robson 1989																																						X					
Roshanai 2009												X																															
Ruparel et al 2019											X																								X								
Sancho-Garnier 2013										X																																	X
Sandiford	X																																										

PRISMA 2020 Checklist

et al 2019																																											
Santare et al 2015																X																											
Segnan 1998							X																															X					
Segura 2001							X																																				
Selva et al 2019														X																													
Senore et al 2015																			X																								
Seow 1998							X																													X							
Shanklemen et al 2014																																								X			
Sharp 1996							X																																				
Smith et al 2010																					X					X										X				X		
Stamatiou 2008																																							X				
Steadman 2004																																							X				
Steck		X											X													X										X				X		

PRISMA 2020 Checklist

elberg 2011																																											
Stein et al 2005						X	X																															X					
Stephens et al 2007						X																																					
Stoffel et al 2019		X																																									
Stoffel et al 2021		X																																									
Sultana 2016										X																		X															
Szarewski 2011							X			X																																	X
Thomas 2014																																							X				
Tinmouth et al 2015																														X													
Tran 2015																				X																			X				
Tranberg 2018										X																																	
Trevena et al		X											X									X					X										X				X		

PRISMA 2020 Checklist

2008																																											
van Roon et al 2011															X			X											X										X			
van Rossum et al 2008															X																											
Verne et al 1993															X																											
Vidal et al 2014																																	X									
Vinker et al 2002																													X										X			
Virtanen 2011							X			X																	X															X
Ward 1991																																					X					
Wardle et al 2003		X																																					X			
Wardle et al 2016															X																								X			
Watson 2006																			X							X					X					X		X				
Watson et al															X																											

PRISMA 2020 Checklist

2013																																											
Watts 2014																		X																					X				
Wikström 2011							X			X																																	X
Williams 1989							X																																				
Wilson 1987							X																															X					
Wilson et al 2015																							X																	X			
Wong 2021			X																																								
Youl 2005																																							X				
Youl 2015								X							X		X																										
Zajac et al 2010																X																											

STUDY CHARACTERISTICS		SAMPLE								INTERVENTION			OUTCOMES					STUDY QUALITY	
Citation	Study design	N (baseline total)	Setting/country	Screening/risk status (initial)	Age (year range or mean)	Gender	Ethnicity	Socioeconomic (IMD)	Cancer type (s)	Duration (evaluation)	Intervention arm(s)	Comparator(s)/control (s)	Outcome measure(s)	Intervention (Arm 1) vs. Comparator	Intervention (Arm 2) vs. Comparator	Intervention (Arm 3) vs. Comparator	Intervention (Arm 4) vs. Comparator	Tool used (by review)	Reviews' assessments

														OR Effect size	OR Effect size	OR Effect size	OR Effect size		
Acera et al 2017	RCT (cluster)	N/A, women were recruited from 4 different participating centres. Each participating centre was assigned one study arm	Primary healthcare services in Barcelona (Spain)	Overdue	30-70	Women	N/A	N/A	Cervical	N/A	1. Personalised letter; 2. Personalised letter with telephone reminder; 3. Information leaflet (printed)	Routine protocol	Screening uptake	Events/total= 1178/2098 vs. 214/1039	Events/total= 3831/6088 vs. 214/1040	Events/total= 2016/3601 vs. 1178/2099	N/A	Cochrane Risk of Bias tool	Unclear risk of bias on all items apart from low risk of incomplete outcome data
Albada 2012	RCT	197	Attendance at a genetic counselling clinic (Netherlands)	N/A	N/A	Women	N/A	N/A	Breast	≤ 3 months	Pre-visit educational website (E-info gene)	Usual care (brief standard pre-visit leaflet)	Risk perception	**SMD (95% CIs)= -0.09 (-0.37, 0.19)**	N/A	N/A	N/A	Cochrane Risk of Bias tool	Low selection bias, high risk due to lack of blinding, low risk due to incomplete outcome data, low risk of selective reporting, unclear for

																			other potential biases
Arcas et al 2014	RCT	703	Spain	N/A	50-69	Women	N/A	N/A	Breast	2 months	Invitation letter and text message reminder 2 days before the mammography appointment	N/A	Screening uptake	Events/total= 182/233 vs. 340/470	N/A	N/A	N/A	Cochrane Risk of Bias tool	Overall rated as high risk of bias
Aubin-Auger et al 2014	RCT (cluster)	45 (GPs)	France	N/A	N/A	N/A	N/A	N/A	Colorectal	7 months	Implementation of a training course focused on communication skills among GPs	N/A	Screening uptake	Unadjusted OR (95% CIs)= 1.22 (1.07, 1.41)	N/A	N/A	N/A	Cochrane Risk of Bias tool	Low risk of bias on all items
Bais 2007	RCT	2,624	Netherlands	Did not respond to the invitation for conventional screening and the first reminder (> 6 months)	30-50	Women	N/A	N/A	Cervical	6 months	Direct mailing of cervicovaginal brush self-sampling kit (a telephone helpline and/or website with information was available throughout the study)	Invitation for conventional cytology with an explanatory letter (a telephone helpline and/or website with information was available throughout the study)	Screening uptake	**Per protocol participation difference (%) between intervention & control (with 95% CIs)= 13.7**	N/A	N/A	N/A	Cochrane Risk of Bias tool	Moderate risk of allocation concealment bias, otherwise low risk of bias on all items

														**(8.7, 18.6)**					
Barthe et al 2015	RCT	3,422	GP practices in Paris (France)	Eligible for screening	50-74	N/A	N/A	N/A	Colorectal	6 months	Standard letter signed by GP inviting patients to visit the GP's office to obtain guaiac-based faecal occult blood test	Standard invitation letter and standard reminder	Screening uptake	Unadjusted OR (95% CIs)= 1.04 (0.83, 1.31)	N/A	N/A	N/A	Cochrane Risk of Bias tool	Overall unclear risk of bias, with unclear risks for random sequence generation, blinding of participants and personnel and blinding of outcome assessment biases, otherwise low risks
Bartholomew et al 2019	RCT	5,271	Auckland (New Zealand)	Non-adherent to initial screening invitation	N/A	N/A	Maori and Pacific residents	N/A	Colorectal	3 months	DVD (6-minute describtion of the importance of screening, ease of test, nature of return,	Usual reminder letter only	Screening uptake	Events/total= 372/2341 vs. 628/2883	N/A	N/A	N/A	Cochrane Risk of Bias tool	Low risk of bias on all items

											participants' positive experiences of diagnostic follow-up test) and a reminder letter								
Baxter and Barata 2011	RCT	193	Canada	N/A	18.43 (mean)	Women	N/A	N/A	Cervical	6 months	Detailed in-depth information (written/verbal) about sexual transmission of HPV	Written information on HPV but did not describe HPV as an STI	Knowledge (about cervical cancer, HPV infection and HPV vaccination)	SMD (95% CIs)= 1.07 (0.75 , 1.39)	N/A	N/A	N/A	Cochrane Risk of Bias tool (version 2)	Overall rated as some concerns: some concerns with randomisation and deviations from intended interventions, otherwise low risk of bias
Beti Thompson 2017	RCT	293	Spain	N/A	43.9 (mean)	Women	N/A	N/A	Cervical	7 months	Web video containing information about cervical cancer screening, encouragement to undergo screening and information about low	Usual care	Screening uptake	Risk ratio (95% CIs)= 1.38 (0.84 , 1.54)	N/A	N/A	N/A	Cochrane Risk of Bias tool	Low risk of bias on all items

											cost clinics where women could go for the screening								
Birkenfeld et al 2011	RCT	16,132	Israel	N/A	N/A	N/A	N/A	A higher socioeconomic status was associated with a higher uptake of screening test	Colorectal	N/A	Faecal immunological test	Faecal occult blood test	Screening uptake	Unadjusted OR (95% CIs)= 0.92 (0.85, 0.99)	N/A	N/A	N/A	Self-modified	Low selection bias, otherwise moderate performance, detection, attrition and reporting biases
Boguradzka et al 2014	RCT	600	A group physician practice of four primary care physicians in the urban areas of Warsaw (Poland)	Eligible for screening	50-65	N/A	N/A	N/A	Colorectal	6 months	Primary care physicians directly recommended screening at the end of health visit. Discussion included information about the benefits of colorectal cancer screening and early treatment plus recommendation to participate in colonoscopy	Primary care physicians prompted patients to obtain a colorectal cancer information leaflet after a scheduled health visit	Screening uptake	Adjusted OR (95% CIs)= 5.33 (3.55, 8.00)	N/A	N/A	N/A	Cochrane Risk of Bias tool	Overall high risk of bias, with high risk for blinding of outcome assessment bias and no intention-to-treat analysis, otherwise low

											screening plus information about the procedure plus help with screening arrangements								risks
Bowman 1995	RCT	342	General practice (Australia)	Overdue	18-70	Women	N/A	N/A	Cervical	6 months	GP signature on reminder letter	Standard letter without signature	Screening uptake	Events/total= 52/178 vs. 26/164	N/A	N/A	N/A	Cochrane Risk of Bias tool	Unclear risk of selection, reporting and other biases, low risk (blinding), high risk (incomplete outcome data)
Brain 2000	RCT	545	2 family cancer clinics in Wales (UK)	N/A	N/A	Women	N/A	N/A	Breast	>3 months	Multidisciplinary genetic assessment	Surgical assessment	Risk perception	SMD (95% CIs)= ≤ 3 months= -0.08 (-0.25, 0.09) >3 months= -0.07 (-0.24, 0.10)	N/A	N/A	N/A	Cochrane Risk of Bias tool	Low risk on items, but unclear for incomplete outcome data and selective reporting

Braithwaite 2005	RCT	72	UK	N/A	N/A	Women (with a family history of breast cancer)	N/A	N/A	Breast	≤ 3 months	GRACE (genetic risk assessment in the clinical environment) tool	Standard genetic risk counselling	Risk perception	SMD (95% CIs)= 0.54 (0.06, 1.01)	N/A	N/A	N/A	Cochrane Risk of Bias tool	Low risk (blinding, other potential biases), but unclear for allocation concealment, incomplete outcome data and selective reporting
Brewer 2021	RCT	3,553	GP clinics (New Zealand)	Never- and under-screened (no screening recorded for at least the last 5 years prior to enrolment)	30-69	Women	Maori, Pacific and Asian ethnicities	N/A	Cervical	N/A	1. Direct mailing of self-sampling kit, preceded by a notification; 2. Direct offer of self-sampling kit at their usual general practice, preceded by a notification	Invitation for conventional cytology (at a clinic, at an independent service providers, or with a study nurse), preceded by a notification	Screening uptake	**Per protocol participation difference (%) between intervention & control (with 95% CIs)=**	**Per protocol participation difference (%) between intervention & control (with 95% CIs)=**	N/A	N/A	Cochrane Risk of Bias tool	Low risk of bias on all items, apart from moderate risk for selective reporting

														**0.11 (0.09, 0.14)**	**0.04 (0.02, 0.05)**				
Broberg 2013	RCT	7,207	71 antenatal health clinics in Western Sweden	Overdue	N/A	Women	N/A	N/A	Cervical	12 months	Telephone call to offer appointment for a smear test (up to 10 attempts made)	Routine care	Screening uptake	Events/total= 718/3207 vs. 422/4000	N/A	N/A	N/A	Cochrane Risk of Bias tool	Low risk of random sequence generation bias, unclear of allocation concealment, blinding, reporting and other biases, high risk of incomplete outcome data
Broc et al 2015	RCT	22,025	France	N/A	50-74	Men and women	N/A	N/A	Colorectal	4 months	Flexible motivational interviews, where counselor gave no information or advice about colorectal cancer screening.	Received reminder mail and fecal occult blood test according to specifications in standard guidelines of national colorectal	Screening uptake	Percentage= 29.5% of 2268 9.2% of 19757	N/A	N/A	N/A	Cochrane Risk of Bias tool	Low risk of reporting, incomplete outcome data and other biases, unclear

											Based on the motivational interviewing, the counselor: let the participants explore their reasons for being (or not being) screened; tailored the session to the subjects' pace. The mean duration was 7.5 minutes, and the interviews were normally completed in 1 call	cancer screening programme							for random sequence generation, allocation concealment and blinding
Buehler et al 1997	RCT	441	2 family medicine clinics (1 urban, 1 rural) affiliated with the Memorial University of Newfoundland (Canada)	Due (had not had Pap test in the past 3 years)	18-69	Women	N/A	N/A	Cervical	N/A	Sent an invitation asking them to seek a Pap test followed by a reminder letter 4 weeks later	No letters sent	Screening uptake	**Events/total= 19/178 vs. 13/208**	N/A	N/A	N/A	Cochrane Risk of Bias tool	Low risk of random sequence generation and incomplete outcome data biases, unclear for all other items

Cadman 2015	RCT	6,000	UK	Did not respond to >2 invitations for screening (> 46 weeks)	25-65	Women	N/A	N/A	Cervical	N/A	Direct mailing of self-sampling kit	Invitation for conventional cytology	Screening uptake	Per protocol participation difference (%) between intervention & control (with 95% CIs)= 2.80 (1.40, 4.10)	N/A	N/A	N/A	Cochrane Risk of Bias tool	Low risk of bias on all items (blinding N/A)
Campbell 1997	RCT	411	New South Wales (Australia)	Had not had a cervical (Pap) smear in previous 30 months	N/A	Women	N/A	N/A	Cervical	N/A	Computer generated printed personalised feedback, listing 'risk factor' of not having a smear within past 2 years	General risk information	Screening uptake	Events/total= 52/148 vs. 33/124	N/A	N/A	N/A	Cochrane Risk of Bias tool	Low risk of biases for incomplete outcome data, baseline comparability and measure against contamination, unclear

																			for allocation concealment, blinding, reporting and funding biases, high for random sequence generation
Clouston et al 2014	RCT	N/A, 39 medical clinic clusters with family physicians and their patients eligible for colorectal screening	Winnipeg, Manitoba (Canada)	N/A	50-70	N/A	N/A	N/A	Colorectal	4 months	Family physicians gave their patients a patient decision aid in the form of a refrigerator magnet, which directed patients to accessing colorectal cancer information and a screening nurse-managed support line and website	Usual care	Screening uptake	**Events/total= 805/2026 663/1837**	N/A	N/A	N/A	Cochrane Risk of Bias tool	Low risk of all biases, apart from unclear for other biases

Clover et al 1996	RCT	2,329	Australia (larger and smaller towns, results for the former extracted due to relevance)	N/A	40-69	Women	N/A	N/A	Breast	N/A	1. Mass media promotion; 2. Family physician involvement	Community participation	Screening uptake	Percentage (with 95% CIs)= 34% vs. 51% (10-24); Z = -4.96; p < .001	Percentage (with 95% CIs)= 68% vs. 51% (10-24); Z = -4.53; p < .01	N/A	N/A	Quality Assessment Tool for Quantitative Studies	Global methodological quality = 'Strong'
Cole et al 2003	RCT	1,818	Electoral roll (Australia)	N/A	50-69	N/A	N/A	N/A	Colorectal	N/A	1. Reducing the required stool samples from three to two, combined with a brush application; 2. Instructions do not include dietary restrictions	1. Kits that requires participant to collect three samples using spatula; 2. Instructions do include dietary restrictions	Screening uptake	RR (95% CIs)= 1.47 (1.28, 1.68)	RR (95% CIs)= 1.50 (1.27, 1.76)	N/A	N/A	Cochrane Risk of Bias tool	Overall low, with low on all items
Cole et al 2007	RCT	1,200	Electoral roll (Australia)	N/A	50-74	N/A	N/A	N/A	Colorectal	N/A	1. Photographs and advocacy statements from lay persons within standard invitation letter; 2. Positively framed information about	1+2. Standard invitation letter containing colorectal cancer prevention and value and ease of screening; 3. Standard invitation pack only	Screening uptake	**RR (95% CIs)= 0.91 (0.79, 1.05)**	**RR (95% CIs)= 1.02 (0.89, 1.17)**	**RR (95% CIs)= 1.22 (1.08, 1.39)**	N/A	Cochrane Risk of Bias tool	Overall unclear, with all items unclear apart from low on blinding of outcome assess

											colorectal cancer risks within standard invitation letter; 3. Advanced notification letter two weeks prior to receiving standard invitation pack								ment and selective reporting
Darlin 2013	RCT	1,500	Sweden	Had not had smears for >9 years	32-65	Women	N/A	N/A	Cervical	N/A	Direct mailing of self-sampling kit. After one month, a reminder including another self-sampling kit was sent to non-responders	Invitation for high-risk HPV testing at an outpatient clinic. The invitation included several alternative appointments. A reminder was sent to non-responders	Screening uptake	**Per protocol participation difference (%) between intervention & control (with 95% CIs)= 10.50 (7.70, 13.30)**	N/A	N/A	N/A	Cochrane Risk of Bias tool	Moderate for all items apart from low for incomplete outcome data

Davidson et al 1999	RCT	100	Family Medical Teaching Centre (Canada)	N/A	50-79	Men	N/A	N/A	Prostate	N/A	Verbal and written information about pros and cons of prostate screening and discussion with doctor at a periodic health examination	Given same information as intervention, but only after second interview	Decisional conflict/ Screening uptake	Mean Difference (95% CIs)= Decisional conflict= -10.44 (-16.10, -4.77)<br><br>Risk ratio (95% CIs)= Actual screening decision= 1.33 (0.88 - 2.00)	N/A	N/A	N/A	Cochrane Risk of Bias tool	Overall high risk of bias, with blinding and allocation concealment at high risk but low for sequence generation. Low risk of incomplete outcome data for decisional conflict and screening decision
Decker et al 2013	RCT (cluster)	31,452	Manitoba (Canada)	Never had screening	30-69	Women	N/A	N/A	Cervical	6 months	Invitation letter and brochure mailed to participants. The invitation letter was personally addressed in	Not mailed an invitation letter but given an index date of screening that matched the	Screening uptake	Events/total= 1010/17068 vs. 441/1438	N/A	N/A	N/A	Cochrane Risk of Bias tool	Low risk of selection biases and incomplete outcom

											English and French and stated that the woman had not had a Pap test in at least 5 years, described the benefits of screening, and provided Pap test locations. Screening availability in all the locations were confirmed to ensure access to screening	invitation date		4					e data, unclear for all other items
Del Mar 1998	RCT	689	On electoral roll in South Brisbane (Australia)	Due and overdue	18-67	Women	Vietnamese	N/A	Cervical	1 year	Personal letter (in Vietnamese) informing them about screening and its benefits	Did not receive a letter	Screening uptake	Events/total= 36/359 vs. 39/330	N/A	N/A	N/A	Cochrane Risk of Bias tool	Low risk of blinding and incomplete outcome data, unclear for all other items
Denters et al 2013	RCT	10,265	National screening program (Netherlands)	N/A	50-75	N/A	N/A	N/A	Colorectal	N/A	Kit that includes feces collection paper for the toilet bowl	Kit that does not include feces collection paper	Screening uptake	Events/total= 2673/5129 vs. 2694/5136	N/A	N/A	N/A	Cochrane Risk of Bias tool	Overall unclear, with low selection biases and incomp

																			lete outcome data, but unclear for all other items
Deutekom et al 2010	RCT	20,623	Municipal records (Netherlands)	N/A	50-75	N/A	N/A	N/A	Colorectal	N/A	Test involves one sample collected with brush (fecal immunochemical test)	Test involves three samples collected with cardboard stick (guaiac-based fecal occult blood test)	Screening uptake	Events/total= 6159/10322 vs. 4839/10301	N/A	N/A	N/A	Cochrane Risk of Bias tool	Overall low, with low on all items apart from unclear for blinding of outcome assessment
Dodd 2019	RCT	N/A	Australia	N/A	N/A	N/A	N/A	N/A	Colorectal	1.5 months	Training course targeted at GPs	Usual care	Screening uptake	Unadjusted OR (95% CIs)= 10.24 (2.90, 36.60)	N/A	N/A	N/A	Downs and Black Checklist	Rated as overall 'good' quality
Dubey et al 2006	RCT	4 clinics (38 physicians) involving 13 preventive health services	St Michael's Hospital/ University of Toronto (Canada)	N/A	N/A	N/A	N/A	N/A	Breast, cervical and colorectal	5 months	Prompt/reminder form with patient gender-specific Preventive Care Checklist Forms©	Usual care	Screening uptake	Relative risk ratio (95% CIs)= 1.41 (0.76	Relative risk ratio (95% CIs)= 0.92 (0.83	Relative risk ratio (95% CIs)= 6.69 (1.90	N/A	Cochrane Risk of Bias tool (adaptio	Publication status, funding source, randomisation method

attached by clerical staff to charts for:<br>1. Mammography;<br>2. Pap test;<br>3. Fecal occult blood test	, 2.61)	, 1.01)	, 24.10)	n)	reported, follow-up period and intention-to-treat analysis reported, abstractors blinded in pre-intervention period and achieved target sample size, 85-90% statistical power, some potential concerns over differences in baseline chcracteristics

Eaker 2004	RCT	12,240	Uppsala county (Sweden)	Due (no Pap smear within past 3 years)	25-59	Women	N/A	N/A	Cervical	5 months	Modified letter (with educational brochure)	Standard letter (without educational brochure)	Screening uptake	Events/total= 1638/6065 vs. 1566/6092	N/A	N/A	N/A	Cochrane Risk of Bias tool	Low risk of allocation concealment and incomplete outcome data biases, unclear for all other items
Elfström 2019	RCT	6,000	Health services (Sweden)	Did not have a screening test on record for at least 10 years, or who had been sent at least 10 annual renewed invitations and who were not blocked from invitations due to hysterectomy or screening program opt-out	33-60	Women	N/A	N/A	Cervical	3 months	1. Direct mailing of self-sampling kit along with an invitation letter and instructions; 2. Opportunity to order a selfsampling kit through an online application	No intervention beyond standard invitation	Screening uptake	**Per protocol participation difference (fractions) between intervention & control (with 95% CIs)= 0.16 (0.14, 0.18)**	**Per protocol participation difference (fractions) between intervention & control (with 95% CIs)= 0.06 (0.05, 0.08)**	N/A	N/A	Cochrane Risk of Bias tool	Low risk of all biases

Emery et al 2019	RCT	551	11 general practices in Perth, Western Australia, and 6 in Melbourne, Victoria (Australia)	N/A	N/A	N/A	N/A	N/A	Lung	12 months	Spirometry was performed then participants were guided through a self-help manual to "increase the salience and personal relevance of symptoms, improve knowledge of symptoms by introducing chest disease prototypes, reinforce the benefits of early intervention in LC and other chest disease, and sanction early consultation." (p.2) Action plans were then developed and linked to symptom checklist. Coping plans were discussed to address barriers to consultation.	Spirometry, brief general discussion on lung health	Knowledge, consultation rates and times	Mean difference (p) Knowledge= 1 month= -0.2 (0.3954) 12 months= -0.1 (0.6083) Relative rate (95% CIs) Respiratory consultations= 1.40 (1.08-1.82) Chi-square (p) Time to first cons	N/A	N/A	N/A	Mixed Methods Appraisal Tool	Yes on all items indicating low risk of bias/high quality

											Monthly prompts (SMS, emails, postcards, phone calls, and fridge magnets) were tailored to individual preferences.			ultation= 1.5923 (0.207) Hazard ratio (95% CIs) Time to present for consultations= 0.827 (0.64, 1.07)					
Enerly 2016	RCT	3,393	Norwegian Cervical Cancer Screening Programme (Norway)	Non-attenders due to receive a second reminder for screening	25-69	Women	N/A	N/A	Cervical	N/A	Sent an information letter, inviting them to participate in the Self-Sampling (SESAM) study. Self-sampling devices were sent to the participants with user instructions, informed consent form, a prepaid return envelope, and	Only sent a second reminder letter according to the Norwegian Cervical Cancer Screening Programme guidelines	Screening uptake	Events/total= 267/800 vs. 601/2593	N/A	N/A	N/A	Cochrane Risk of Bias tool	Low risk on all items, apart from unclear for other bias

											a questionnaire to collect information on attitudes and ease of self-sampling, and to identify reasons for nonattendance according to recommendations								
Evans 2010	RCT	514	Wales	N/A	N/A	Men	N/A	N/A	Prostate	N/A	Online programme on options' outcomes, clinical problem, outcome probabilities, explicit values clarification, others' opinion, guidance (interactive computer programme; summary)	Usual care	Knowledge, decisional conflict, screening uptake	Mean (n) Knowledge= 4.9 (89) vs. 2.17 (103) Mean (SD) Decisional conflict= 38.1 (24.2) (n=89) vs. 49.6 (24.2) (n=103) Even	N/A	N/A	N/A	Cochrane Risk of Bias tool	Low risk on all items, apart from unclear for random sequence generation and blinding of participants and personnel

														ts/total Screening uptake= 4/127 vs. 11/123					
Federici et al 2005	RCT (cluster)	7,332	Italy	N/A	N/A	N/A	N/A	N/A	Colorectal	N/A	Faecal immunological test	Guaiac-based faecal occult blood test	Screening uptake	Unadjusted OR (95% CIs)= 1.29 (1.17, 1.43)	N/A	N/A	N/A	Self-modified	Low reporting bias, otherwise moderate selection, performance, detection and attrition biases
Federici et al 2006	RCT (cluster)	7332 patients (distributed amongst 130 GP for intervention, 13 hospital for comparison)	Italy	N/A	N/A	N/A	N/A	N/A	Colorectal	45 days	Economic incentive €1,000 ($CAN1.57, 2008 10 31) for GP's participation, €10 for each patient they screened (Guaiac and immunochemical faecal occult blood test)	Usual care (Guaiac and immunochemical faecal occult blood test without financial incentive)	Screening uptake	Relative risk ratio (95% CIs)= 3.40 (3.13, 3.70)	N/A	N/A	N/A	Cochrane Risk of Bias tool (adaption)	Publication status and follow-up period reported, target sample size achieved and 90% statistic

																			al power, funding source, randomisation method, baseline characteristics for GPs, blinding and intention-to-treat analysis not reported
Firmino-Machado et al 2019	RCT	1,220	Portugal	N/A	25-49	Women	N/A	N/A	Cervical	45 days	Automated or customised text messages and phone calls, followed by text message reminders of the appointment	Usual care	Screening uptake	Events/total= 103/202 vs. 70/205	N/A	N/A	N/A	Cochrane Risk of Bias tool (version 2)	Overall rated as unclear risk of bias, but only unclear for randomisation bias and low on all other items

Fry 2003	RCT	373	UK	Referred to the regional clinical genetics department for breast cancer genetic risk counselling	N/A	Women	N/A	N/A	Breast	6 months	Novel community-based service	Standard regional service	Risk perception	Percentage perceived their risk to be moderate or high= 4 weeks= 92% (n=129) vs. 92% (n=147) 6 months= 91% (n=123) vs. 92% (n=140)	N/A	N/A	N/A	Cochrane Risk of Bias tool	Randomisation sequence and allocation both rated as adequate and other potential risks of bias were discussed, but blinding 'not adequately described', incomplete outcome data 'unclearly described' and there was risk of bias due to selective reporting

Fujiwara et al 2015	RCT	1,912	Urban area (Japan)	Had not participated in screening for more than a year ('non-adherent')	20-39	Women	N/A	N/A	Cervical	N/A	1. Received a printed reminder with information on the possible benefits of screening;<br>2. Received a printed reminder with information on the possible benefits and risk of screening	Received a printed reminder with simple information	Screening uptake	Percentage= 11.4% of 622 vs. 4.9% of 650	Percentage= 10.3% of 640 vs. 4.9% of 650	N/A	N/A	Cochrane Risk of Bias tool	Low risk of all biases, apart from unclear for other biases
Gabel et al 2020	RCT	1,723	National screening programme (Denmark)	N/A	53-74	N/A	N/A	N/A	Colorectal	N/A	Web-based decision aid	Nothing apart from national information pamphlet	Screening uptake, knowledge, attitudes, decisional conflict	Unadjusted OR (95% CIs) Screening uptake= 1.40 (1.16, 1.71) Absolute difference (95% CIs) Knowledge= 0.09 (-0.05,	N/A	N/A	N/A	Critical appraisal instruments from Joanna Briggs Institute for experimental and quasi-experimental studi	Yes on all items apart from blinding

														0.24) Absolute difference (95% CIs) Attitudes = 0.45 (0.00, 0.91) Unadjusted OR (95% CIs) Decisional conflict= -0.11 (-0.20, -0.01)				es	
Gattellari 2003	RCT	248	Australia	N/A	N/A	Men	N/A	N/A	Prostate	N/A	Pamphlet on options' outcomes, clinical problem, outcome probability, explicit values clarification	Usual care using brief information on screening test and chances of false-positive results	Screening uptake, knowledge, accurate risk perceptions, decisional conflict	Events/total Screening uptake= 27/106 vs. 25/108 Mean	N/A	N/A	N/A	Cochrane Risk of Bias tool	Low risk of allocation concealment, detection and other bias, unclear for all other items

PRISMA 2020 Checklist

(SD) Knowledge= 50 (18.4) (n=106) vs. 45 (15.9) (n=108) Events/total Accurate risk perceptions= 57/106 vs. 11/108 Mean (SD) Decisional conflict= 42.5 (20) (n=106) vs. 42.5

														(33.3) (n=108)					
Gattellari 2005	RCT	421	Australia	N/A	N/A	Men	N/A	N/A	Prostate	N/A	Pamphlet on options' outcomes, clinical problem, outcome probability, explicit values clarification	Video on clinical problem, outcome probability, others' opinion, or usual care using brief information on screening test and chances of false-positive results	Screening uptake, knowledge, decisional conflict	Events/total Screening uptake= 37/131 vs. 42/136 Mean (SD) Knowledge= 57.2 (21.3) (n=131) vs. 42.2 (16.7) (n=136) Mean (SD) Decis	N/A	N/A	N/A	Cochrane Risk of Bias tool	Low risk of bias on all items apart from unclear for selective reporting

														ional conflict= 30.8 (19.3) (n=131) vs. 29.2 (15) (n=136)					
Gimeno-Garcia et al 2009	RCT	158	Spain	N/A	N/A	N/A	N/A	N/A	Colorectal	12 months	Video-based educational intervention + standard letter	Standard letter only	Screening uptake	Unadjusted OR (95% CIs)= 1.91 (0.95, 3.89)	N/A	N/A	N/A	Cochrane Risk of Bias tool	Overall high risk of bias, with unclear selection bias and inadequate/selective reporting, unclear risk of other biases
Giorgi 2000	RCT	8,634	Italy	Non-responders	N/A	Women	N/A	N/A	Breast	N/A	Letter from GP	Standard letter	Screening uptake	Events/total= 1897/2552 vs. 3875/6082	N/A	N/A	N/A	Cochrane Risk of Bias tool + CASP criteria	Unclear sequence generation, high risk of concealment and blindin

																			g of assessor biases, otherwise low risks
Giorgi Rossi 2015	RCT	14,041	Organised screening programmes in six local health authorities (Northern Italy)	Had not responded to an earlier screening invitation letter	30-64	Women	N/A	N/A	Cervical	N/A	1. Received self-sampler by mail directly at home. This was preceded by an explanatory letter sent one week earlier;<br>2. Offered opportunity to pick the self-sampling device up at an area pharmacy	Received a standard invitation letter to perform either a Pap test or an HPV test at the clinic according to that centre's routine screening	Screening uptake	Percentage= 21.6 % of 4516 vs. 11.9 % of 5012	Percentage= 12.0 % of 4513 vs. 11.9 % of 5012	N/A	N/A	Cochrane Risk of Bias tool	Low risk on all items, apart from unclear for other bias
Glazebrook et al 2006	RCT	589	Participants at high risk of developing melanoma recruited from Family Practices within Nottinghamshire (UK)	N/A	N/A	N/A	N/A	N/A	Skin	6 months	Multimedia programme called "Skinsafe" with eight sections designed to be completed in 10 to 15 minutes. Included animation, photographs and simple text to inform users about the dangers	Usual care	Screening uptake, knowledge (regarding screening test/condition concerned)	Adjusted OR (95% CIs) Screening uptake= 1.67 (1.04, 2.69) SMD (95% CIs) Kno	N/A	N/A	N/A	Cochrane Risk of Bias tool	Low risk of random sequence generation and other biases, high for selective reporting, unclear on all remaini

											from excessive sun exposure; how to protect skin from the sun; characteristics of skin at risk; early signs of melanoma; how to reduce risk from melanoma; how to check skin for suspicious lesions. The last section was designed to provide individualised feedback on the persons relative risk for skin cancer. Health Belief Model was used to base the intervention			wledge= 0.40 (0.23, 0.56)					ng items
Gold 2011	RCT (parallel)	358	Community dwellers (Australia)	N/A	N/A	39.9% women	N/A	N/A	Skin	4 months	Fortnightly humorous and short text message reminders for improving sun protection habits that used informal language and were linked to	Usual care	Behaviour/knowledge	Risk ratio (95% CIs) Consideration of the long-term	N/A	N/A	N/A	Cochrane Risk of Bias tool (version 2)	Overall high risk, with high risk of bias due to deviations from

											particular annual events where possible			consequences of prolonged UV exposure= 1.01 (0.84 , 1.20)					intended interventions, otherwise some concerns for all other items
Guiriguet et al 2016	RCT (cluster)	41,042	National (Spain)	No prior screening	58.7 (mean)	53.6% women	N/A	N/A	Colorectal	1 year	Received an electronic alert reminding patients to discuss screening	Did not receive a reminder	Screening uptake	Events/total= 9536/21619 vs. 8196/19423	N/A	N/A	N/A	Cochrane Risk of Bias tool	Overall low risk, with low risk of bias on all items
Gummersbach 2015	RCT	792	Family practices in the federal state of North-Rhine–Westphalia (Germany)	Had not yet received their first invitation to be screened but are just about to receive it	48.67 (intervention mean); 48.76 (control mean)	Women	93.26% native German language (intervention); 89.71% native German language (control)	N/A	Breast	N/A	Edge flyer format leaflet with evidence-based information about the potential benefits and harms of screening. The information included: benefit, mortality reduction, sensitivity, specificity,	Edge flyer format leaflet that promotes breast cancer screening with limited information about the benefit and sensitivity of mammography screening, the rate of a pathological result of	Knowledge, decisional conflict	Knowledge SMD (95% CIs)= 0.13 (-0.09 , 0.34) Decisional conflict SMD (95% CIs)= 0.32 (0.11	N/A	N/A	N/A	Cochrane Risk of Bias tool (version 2)	Overall low risk, with low risk of bias on all items

											number needed to screen, overdiagnosis, false-positive results rate, increase of operation and radiation of women who do not benefit from mammography screening, the rate of a pathological result of screening, interval cancer, and recommended to self-check for breast cancer	screening, interval cancer, the potential side effects of X-ray		, 0.53)					
Hagoel et al 2016	RCT	48,091	National database (Israel)	No colonoscopy in past 3 years, no fecal occult blood test in previous 1 year	50-74	N/A	N/A	N/A	Colorectal	6 months	Interrogative reminders, with or without reference to social context	Noninterrogative reminders, with or without social context, or no reminders at all	Screening uptake	Adjusted OR (95% CIs)= 1.11 (1.05 , 1.19)	N/A	N/A	N/A	Cochrane Risk of Bias tool (version 2)	Overall low risk, with low risk of bias on all items
Haguenoer 2015	RCT	5,998	Regional screening programme (France)	Overdue	30-65	Women	N/A	N/A	Cervical	9 months	1. Recall invitation/reminder for Pap smear (intervention) vs. No intervention (control);	See intervention arms for comparisons	Screening uptake	Events/total= 233/ 2000 vs. 198/ 1999	Events/total= 450/ 1999 vs. 233/ 2000	N/A	N/A	Cochrane Risk of Bias tool	Low risk of selection and incomplete outcome data

PRISMA 2020 Checklist

										2. Direct mailing of HPV self-sampling kit (intervention) vs. Recall invitation for Pap smear (control)								biases, unclear for all other items
Hegenscheid 2011	RCT 10,954	Germany	Non-responders	50-69	Women	N/A	N/A	Breast	N/A	Phone call reminder in addition to invitation letter	Invitation letter only	Screening uptake	Events/total= 728/ 2455 vs. 770/ 2952	N/A	N/A	N/A	Cochrane Risk of Bias tool + CASP criteria	High risk of concealment bias, some concerns regarding blinding of assessor, otherwise low risks on all items
Heranney 2011	RCT 10,662	Organised screening (France)	Overdue	N/A	Women	N/A	N/A	Cervical	8 months	Telephone reminder that Pap smears were necessary. Ten attempts were made to contact	Letter invitation for a Pap smear	Screening uptake	Events/total= 335/ 5310 vs. 309/ 5352	N/A	N/A	N/A	Cochrane Risk of Bias tool	Low risk of incomplete outcome data, but otherwise unclear risks on all items

Heverin 2011	RCT	78	Community, university (Ireland)	N/A	18-32	Men	N/A	N/A	Prostate	2 months	Testicular self-examination demonstration video and once or twice implementation intention	Testicular self-examination demonstration video only	Screening uptake	Events/total= 43/53 vs. 21/25	N/A	N/A	N/A	Cochrane Risk of Bias tool	High risks of sequence generation, allocation concealment and blinding of participants biases, otherwise low risks
Hewitson 2011	RCT	1,288	UK	N/A	N/A	N/A	N/A	N/A	Colorectal	20 weeks	1. GP-signed invitation letter to promote fecal occult blood test;<br>2. Enhanced procedural information leaflet to promote fecal occult blood test	A fecal occult blood test kit was only sent a week after first mailed letter	Screening uptake	Unadjusted OR (95% CIs)= 1.26 (1.01, 1.58)	Unadjusted OR (95% CIs)= 1.26 (1.01, 1.58)	N/A	N/A	Cochrane Risk of Bias tool	Overall low risk, with low risk of bias on all items
Hirst et al 2017	RCT	8,269	London (UK)	N/A	60-74	Men and women	N/A	N/A	Colorectal	4 months	Automated text message reminder. Frequency of text message reminder: 1	Usual care: mailed fecal occult blood test kit with printed reminders	Screening uptake	Events/total= 1674/4134 vs. 1648/4135	N/A	N/A	N/A	Cochrane Risk of Bias tool	Overall low risk, with low risk of bias on all items apart from

																			unclear for selective reporting
Hoare 1994	RCT	498	UK	N/A	N/A	Women	N/A	N/A	Breast	N/A	Trained link-workers contacted all women a few weeks before invitations were sent. If no information was obtained, a second visit was made. Link-workers conducted interviews in an appropriate language, using a semi-structured questionnaire. A short explanation about breast screening was provided and women were encouraged to take up a forthcoming invitation	Received no visits	Screening uptake	Events/total= 122/247 vs. 117/251	N/A	N/A	N/A	Cochrane Risk of Bias tool + CASP criteria	High risk or concerns relating to all items, apart from baseline comparability and selective outcome reporting
Hol et al 2010	RCT	15,011	Netherlands	N/A	N/A	N/A	N/A	N/A	Colorectal	1 year	Use of fecal immunochemical test and patient mailed	Usual care	Screening uptake	Unadjusted OR (95% CIs)=	N/A	N/A	N/A	Self-modified	Low risks of selection, attritio

											reminders			1.63 (1.50, 1.77)					n and reporting biases, moderate for performance and detection bias
Hong 2014	RCT	923	Community (South Korea)	N/A	50-59	Men	N/A	N/A	Colorectal	3 months	1. Paper-based educational information sent by mail; 2. Educational information and promotion to undergo screening through telephone counselling	Usual care	Screening uptake	Events/total= 38/230 vs. 30/223	Events/total= 56/243 vs. 30/224	N/A	N/A	Cochrane Risk of Bias tool	Low risks of blinding of assessor, incomplete outcome data and selective outcome reporting biases, otherwise unclear risks
Hou et al 2005	RCT	424	Taiwan	Had not had a Pap test in the previous 12 months	30 years and older	Women	Chinese	N/A	Cervical	3 months	Three-month program utilising direct mail communication as well as a phone-counseling component,	Received a monthly newsletter with health information in general from the hospital	Screening uptake	Percentage= 50% of 212 vs. 32% of	N/A	N/A	N/A	Cochrane Risk of Bias tool	Low risks of blinding and selective reporting biases,

PRISMA 2020 Checklist

											received educational brochures with theory and evidence-based messages			212					high of selection and incomplete outcome data biases, unclear of other bias
Huf et al 2020	RCT	14,587	NorthWest London (UK)	N/A	24-64	Women	N/A	N/A	Cervical	18 weeks	1. SMS without manipulation; 2. Primary care physician endorsed SMS	No SMS	Screening uptake	Adjusted OR (95% CIs)= 1.18 (1.02, 1.37)	Adjusted OR (95% CIs)= 1.19 (1.03, 1.38)	N/A	N/A	Cochrane Risk of Bias tool (version 2)	Overall low risk of bias, with low risks on all items
Hughes et al 2005	RCT (cluster)	3,358	GP records (rural Australia)	N/A	50-74	N/A	N/A	N/A	Colorectal	N/A	Test involves two samples collected with brush and no diet restrictions (fecal immunochemical test)	Test involves three samples collected with spatula and includes diet restrictions (guaiac-based faecal occult blood test)	Screening uptake	Unadjusted OR (95% CIs)= 1.93 (1.61, 2.31)	N/A	N/A	N/A	Cochrane Risk of Bias tool	Overall high risk of bias, with high risks for selection, incomplete outcome data and other biases, while unclear for blinding and

																			selective reporting
Hunt 1998	RCT	372	Community - identified from files at a women's clinic staffed by Aboriginal health workers in Danila Bilba (Australia)	Overdue	N/A	Women	Aboriginal	N/A	Cervical	3 months	1. Personal approach being approached by Aboriginal health workers and invited for screening;<br>2. Letter designed by Aboriginal workers stating individuals overdue for smear and inviting them to attend	Usual care with reminder tags for clinic staff attached to medical records	Screening uptake	Events/total= 4/60 vs. 0/61	Events/total= 2/63 vs. 0/61	N/A	N/A	Cochrane Risk of Bias tool	Low risk for random sequence generation, blinding and incomplete outcome data biases, unclear for allocation concealment, selective reporting and other biases
Ilic 2008	RCT	161	Community and online (Australia)	Had not previously been tested	>45 years	Men	N/A	N/A	Prostate	N/A	1. Mailed video containing standard educational information about prostate cancer;<br>2. Website containing standard	Mailed 28-pages pamphlet containing standard educational information about prostate cancer (content/messages	Screening uptake	Events/total= 32/53 vs. 34/49	Events/total= 42/54 vs. 34/49	N/A	N/A	Cochrane Risk of Bias tool	Low risks of bias on all items, apart from unclear for blinding of particip

											educational information about prostate cancer	identical as for intervention groups)							ants
Ingrand et al 2016	RCT (cluster)	304	France	Siblings of patients diagnosed with colorectal cancer or adenomatous polyps	N/A	N/A	N/A	N/A	Colorectal	12 months	Specialised screening nurse (familiar with applicability and constraints of colonoscopy) performed counselling via telephone interviews and materials by mails to inform participants of the increased risk of colorectal cancer and benefits of colonoscopy. The information was tailored according to each participant’s psychosocial characteristics	Usual care	Screening uptake	Events/total= 90/160 vs. 51/144	N/A	N/A	N/A	Cochrane Risk of Bias tool (version 2)	Overall low risk of bias, with low risks on all items
Ishikawa et al 2012	RCT	3,236	Listed in local health department database (Japan)	No screening in past 2 years	51-59 (except 55 years)	Women	N/A	N/A	Breast	5 months	Individual assessment, assessment-based tailored letter to prompt study participants	Non-tailored reminder	Screening uptake	OR (95% CIs)= 4.02 (2.67, 6.06)	N/A	N/A	N/A	Critical appraisal instruments	Total score 9, with 'no' for similarity betwee

PRISMA 2020 Checklist

										to participate in mammography screening (divided into three segments: high intention; low intention and high breast cancer worry; low intention and low breast cancer worry), participants then had to return postcard to receive tickets for free screening, which they could use at local clinics							from Joanna Briggs Institute for RCTs	n groups at baselin and whether identical treatment of groups, unclear for concealed allocation and blinding of participants, otherwise 'yes' on all items
Jalili 2019 RCT	1,052	Canada	Unscreened women: no Pap test in registry and who had been registered for 5+ years. Non-responders: women who had been sent an invitation letter to be screened, but who	30-65	Women	N/A	N/A	Cervical	6 months	Direct mailing of self-sampling kit + reminder letter after 8 weeks for non-responders	No intervention beyond standard invitation	Screening uptake	Per protocol participation difference (fractions) between intervention &	N/A	N/A	N/A	Cochrane Risk of Bias tool	Low risks of bias on all items, apart from moderate for random sequence generation (not docum

				remained unscreened										control (with 95% CIs)= 0.07 (0.04, 0.10)					ented in study)
Janda 2014	RCT	930	Community (Australia)	No previous history of melanoma	> 50 years	Men	N/A	N/A	Skin	7 months	Video-based skin awareness educational materials with a message from national sports personality and melanoma survivors + written educational materials	Written educational materials only	Screening uptake	Events/total= 246/436 vs. 229/434	N/A	N/A	N/A	Cochrane Risk of Bias tool	Low risks on all items
Janda et al 2011	RCT	929	Community (Australia)	N/A	>50 years	Men	N/A	N/A	Skin	N/A	Researcher guide and colour brochure containing educational information + body chart diagram + video/DVD + two postcard reminders	Researcher guide and colour brochure only	Screening uptake	Events/total= 153/420 vs. 126/411	N/A	N/A	N/A	Cochrane Risk of Bias tool	Overall rated as high methodological quality, with low risks of bias on all items apart from unclear for allocation

																			concealment and blinding of participants
Jensen et al 2009	RCT (cluster)	14,979	Local screening programme for cancer (Denmark)	Overdue	23-59	Women	N/A	N/A	Cervical	9 months	Personalised targeted invitation letter from GP. GP also received visit from facilitator to discuss ways to increase uptake of screening	Standard invitation letter only	Screening uptake	Events/total= 1448/7527 vs. 947/7452	N/A	N/A	N/A	Cochrane Risk of Bias tool	Unclear risks of bias on all items
Kellen 2018	RCT	35,895	Belgium	Without screening record since 8 years	30-64	Women	N/A	N/A	Cervical	1 year	1. Reminder mailing with self-sampling kit (mail-to-all);<br>2. Reminder mailing with self-sampling to be ordered (opt-in)	1. Reminder mailing inviting women to have a cytology specimen taken by a clinician (=routine intervention);<br>2. No invitation	Screening uptake	Per protocol participation difference (fractions) between intervention & control (with 95% CIs)= no letter comp	Per protocol participation difference (fractions) between intervention & control (with 95% CIs)= no letter comp	N/A	N/A	Cochrane Risk of Bias tool	Low risks of bias on all items, apart from high for allocation concealment and moderate for selective reporting

														arison= 0.11 (0.10, 0.12); recall letter comparison= 0.08 (0.07, 0.09)	arison= 0.03 (0.02, 0.03); recall letter comparison= 0.00 (-0.01, 0.01)				
Kerrison et al 2015	RCT	2,240	UK	Due to be invited for first routine screen	47-53	Women	N/A	N/A	Breast	N/A	Text message reminder 48 hours before the appointment and an additional text message if they did not attend the initial appointment	Usual care	Screening uptake (proportion attending the appointment within 60 days of the initial appointment)	Events/total= 759/1122 vs. 703/1118	N/A	N/A	N/A	Cochrane Risk of Bias tool (version 2)	Overall low risk of bias, with low risks on all items

King et al 1994	RCT (cluster)	1,281	GP records (Australia)	N/A	45-75	N/A	N/A	N/A	Colorectal	N/A	Low literacy booklet with graphics and risk information sent with invitation pack	Standard invitation pack only	Screening uptake	Risk ratio (95% CIs)= 1.01 (0.92, 1.12)	N/A	N/A	N/A	Cochrane Risk of Bias tool	Overall unclear risk of bias, with unclear risk for selection, blinding of participants and personell and selective reporting biases, otherwise low
Kitchener 2017	RCT (cluster)	6,213	UK	Due for their first invitation and who in phase 1 of the 'STRATEGIC trial' did not respond to invitation letters (with or without pre-leaflet or with/without online booking) to screening after 6 months	20 (Grampian); 25 (Manchester)	Women	N/A	N/A	Cervical	18 months	1. Direct mailing of unrequested self-sampling kits (mail-to-all);<br>2. Direct mailing of requested self-sampling kits (opt-in)	No intervention beyond standard invitation	Screening uptake	**Per protocol participation difference (fractions) between intervention & control (with 95%**	**Per protocol participation difference (fractions) between intervention & control (with 95%**	N/A	N/A	Cochrane Risk of Bias tool	Moderate risks of selection biases, otherwise low

														**CIs)= -0.09 (-0.11, -0.07)**	**CIs)= -0.15 (-0.16, -0.13)**				
Kregting 2020	RCT	1,312	South West screening region of the Netherlands	N/A	60.1 (intervention mean); 59.9 (control mean)	Women	N/A	N/A	Breast	N/A	Official breast cancer screening information leaflet from the Dutch National Institute for Public Health and the Environment. The leaflet was developed based on the opinion of experts and contained information about: a) the screening invitation; b) the screening process; c) possible screening outcomes; and d)	No intervention	Knowledge, informed choice	**Knowledge SMD (95% CIs)= 0.21 (0.09, 0.34) Informed choice Risk ratio (95% CIs)= 1.07 (0.99, 1.15)**	N/A	N/A	N/A	Cochrane Risk of Bias tool (version 2)	Concerns related to the randomisation process and selection of the reported results

											benefits and harms (overdiagnosis, overtreatment, false-negatives and interval cancers)								
Lancaster 1992	RCT	2,131	General practices in North Manchester (UK)	Due	50-64	Women	N/A	N/A	Cervical	N/A	Cervical screening invitation sent with breast screening invitation	Breast screening invitation only sent	Screening uptake	Events/total= 151/908 vs. 89/886	N/A	N/A	N/A	Cochrane Risk of Bias tool	Low risk of incomplete outcome data, unclear risks on all other items
Levi et al 2011	RCT	12,537	Israel	N/A	N/A	N/A	N/A	N/A	Colorectal	N/A	Faecal immunological test	Guaiac-based faecal occult blood test	Screening uptake	Unadjusted OR (95% CIs)= 0.86 (0.80, 0.94)	N/A	N/A	N/A	Self-modified	Low reporting bias, otherwise moderate selection, performance, detection and attrition biases

PRISMA 2020 Checklist

Libby et al 2011	RCT	59,953	National screening programme (Scotland)	N/A	50-74	N/A	N/A	N/A	Colorectal	N/A	1. "Know the facts" information booklet included with advance notification letter; 2. Advanced notification letter sent prior to standard invitation pack	1. Advance notification without booklet and followed by standard invitation pack; 2. Standard invitation pack only	Screening uptake	Risk ratio (95% CIs)= 0.99 (0.98, 1.01)	Risk ratio (95% CIs)= 1.09 (1.08, 1.11)	N/A	N/A	Cochrane Risk of Bias tool	Overall low risk of bias, with low risks on all items apart from unclear for incomplete outcome data
Lilliecreutz 2020	RCT	6,606	Sweden	Did not respond to invitation for conventional screening and had not had a smear test in 6 years (ages 30-49) and 8 years (ages 50-64)	30-64	Women	N/A	N/A	Cervical	6 months	Direct mailing of self-sampling kit + annual invitation	No intervention beyond standard invitation	Screening uptake	Per protocol participation difference (fractions) between intervention & control (with 95% CIs)= 0.19 (0.17, 0.20)	N/A	N/A	N/A	Cochrane Risk of Bias tool	Low risks of bias on all items

Lo et al 2014	RCT	23,180	National screening programme (UK)	N/A	60-69	N/A	N/A	N/A	Colorectal	N/A	Pre-formulated implementation intentions inserted into standard leaflet	Standard leaflet only	Screening uptake	Risk ratio (95% CIs)= 0.98 (0.95, 1.01)	N/A	N/A	N/A	Cochrane Risk of Bias tool	Overall unclear risk of bias, with unclear risks for allocation concealment, blinding of participants and personell and selective reporting biases, otherwise low
Lonnberg 2016	RCT	1,036	National screening programme (Norway)	Overdue (no smear within the past ~4 years)	25-69	Women	N/A	N/A	Cervical	6 months	Reminder letter with a scheduled appointment in 2-4 weeks time. There were limited possibilities for rescheduling, and appointments were primarily during normal office hours. Women were	Standard open reminder	Screening uptake	Events/total= 196/526 vs. 102/510	N/A	N/A	N/A	Cochrane Risk of Bias tool	Low risks of random sequence generation and incomplete outcome data biases, otherwise unclear risks on all

											not required to confirm their attendance in advance								items
MacDonald 2021	RCT (cluster)	538	New Zealand	Had not had a smear test in ≥4 years	25-69	Women	N/A	N/A	Cervical	N/A	Direct offer of self-sampling kit when attending intervention clinics. Participants randomised to receive the intervention could opt for a clinician taken HPV test or cervical smear	Offered a cervical smear when attending control clinics	Screening uptake	Per protocol participation difference (fractions) between intervention & control (with 95% CIs)= 0.20 (0.16, 0.25)	N/A	N/A	N/A	Cochrane Risk of Bias tool	Moderate risks for random sequence generation and reporting of timelines biases (under Reporting), otherwise low risks of biases including for selective reporting
MACS Group 2006	RCT	1,333	Australia	N/A	N/A	N/A	N/A	N/A	Colorectal	N/A	Use of choice between different screening tests (fecal immunochemical test, colonoscopy, flexible sigmoidoscopy plus fecal	Usual care	Screening uptake	Unadjusted OR (95% CIs)= 1.65 (1.04, 2.64)	N/A	N/A	N/A	Self-modified	Low risks for selection, attrition and reporting biases, moderate for

											immunochemical test)								performance and detection bias
Mant 1992	RCT	1,588	UK	N/A	N/A	N/A	N/A	N/A	Colorectal	N/A	1. Advanced notification letter; 2. Postal mailing of fecal occult blood test kits	Invitation for a health check	Screening uptake	Unadjusted OR (95% CIs)= 1.35 (0.99, 1.87)	Unadjusted OR (95% CIs)= 1.31 (0.98, 1.85)	N/A	N/A	Self-modified	Moderate risks for selection and performance and detection biases, high for attrition and reporting biases
Mathieu 2007	RCT	734	Australia	Considering a subsequent screening	70-71	Women	N/A	N/A	Breast	N/A	Booklet on options' outcomes, clinical problem, outcome probability, explicit values clarification, others' opinions, guidance with worksheet (Ottawa Decision Support Framework)	BreastScreen New South Wales brochure - includes information for women 70 + but no numeric information about the outcomes of screening	Multiple	Mean increase Knowledge= 2.62 (n=351) vs. 0.68 (n=357) (p< 0.001) Mean (SD)	N/A	N/A	N/A	Cochrane Risk of Bias tool	Low risks on all items, apart from unclear for blinding of participants and personnel

Decis
ional
confli
ct=
20.1
(14.5
)
(n=3
15)
vs.
21.9
(14.5
)
(n=2
95)
Even
ts/to
tal
Infor
med
value
s-
choic
e
cong
ruen
ce=
227/
309
vs.
136/
279
Even
ts/to
tal
Prop
ortio
n
unde
cided
=
17/3

PRISMA 2020 Checklist

														49 vs. 36/356 Mean Anxiety immediately after = 29.61 (n=321) vs. 29.34 (n=315)					
Mathieu 2010	RCT	412	Australia	Considering screening	N/A	Women	N/A	N/A	Breast	N/A	Internet programme + worksheet on options' outcomes, clinical problem, outcome probabilities, explicit values clarification, others' opinions, guidance (worksheet with questions relevant to decision-making process; one or more	Delayed intervention	Multiple	Mean Screening uptake= 52% (n=96) vs. 65% (n=127) (p= 0.05) Mean (SD) Knowledge= 73.5 (27.6	N/A	N/A	N/A	Cochrane Risk of Bias tool	Low risks on all items, apart from unclear for allocation concealment, blinding of participants and personnel and selective reporti

questions that asked patients to clarify their preferences; summary)	) (n=113) vs. 62.7 (27.6) (n=189) Mean Decisional conflict= 71% (n=91) vs. 64% (n=110) (p= 0.24) Events/total Informed values-choice congruence= 65/91 vs. 70/110 Events/total	ng biases

														Proportion undecided = 21/117 vs. 82/209 Mean Accurate risk perception= 3.02 (n=113) vs. 2.45 (n=189) (p< 0.001)					
McAvoy 1991	RCT	737	National screening programme in Leicester (England)	Overdue (not recorded as having had a smear test)	18-52	Women	Asian	N/A	Cervical	4 months	1. Posted multilingual printed material; 2. Face-to-face home visits with multilingual material	Received no intervention	Screening uptake	Events/total= 14/131 vs. 3/62	Risk ratio (95% CIs)= 5.83 (1.92, 17.67)	N/A	N/A	Cochrane Risk of Bias tool	Low risks of random sequence generation and incomplete outcome data biases, otherwi

																			se unclear risks on all items
McCaffery 2010	RCT	210	Australia	N/A	N/A	Women	N/A	N/A	Cervical	N/A	Written material	No information; repeat screening in six months	Knowledge (screening)	Mean (SD)= 81 (23.51) (n=77) vs. 72 (23.51) (n=71)	N/A	N/A	N/A	Cochrane Risk of Bias tool	Low risks on all items, apart from unclear for blinding of participants and personnel
McDowell 1989	RCT	2,034	Hospital-based family medical centre in Ottawa (Canada)	Due and overdue (no previous smear in past year)	18-35	Women	N/A	N/A	Cervical	1 year	1. GP letter and reminder letter after 21 days;<br>2. Telephone call	Usual care	Screening uptake	Events/total= 38/184 vs. 18/165	Events/total= 30/189 vs. 18/165	N/A	N/A	Cochrane Risk of Bias tool	Unclear risks of bias on all items, apart from low risk for incomplete outcome data
Moen et al 2020	RCT	10,360	Subdistricts of Bergen (Norway)	Non-exposed'	25-69	Women	Described as 'multi-ethnic'	N/A	Cervical	6 months	Multifaceted delivered in participants' languages	No intervention	Screening uptake	Risk ratio (95% CIs)= 1.12 (1.08, 1.16)	N/A	N/A	N/A	Effective Public Health Care Practice	Rated as moderate quality

																		Project (EPHPP) quantitative study quality assessment tool	
Morrell et al 2005	RCT	90,000	Community - New South Wales Pap Test Register (Australia)	Due (had not had a smear test for > 48 months)	20-69	Women	N/A	N/A	Cervical	90 days	Letter identical to that usually sent out to women at 27 months after latest Pap smear or letter giving a similar message, but phrased in a tone more sympathetic to other factors going on in the woman's life that might have stopped her from having the test to date	No intervention	Screening uptake	Events/total= 2630/59780 vs. 868/29919	N/A	N/A	N/A	Cochrane Risk of Bias tool	Unclear risks on all items, apart from low risk for incomplete outcome data
Moss et al 2017	RCT (cluster)	11,67,017	National screening programme (UK)	N/A	60-74	N/A	N/A	N/A	Colorectal	N/A	Participants issued fecal immunochemical test	Participants issued guaiac-based fecal	Screening uptake	Risk ratio (95% CIs)=	N/A	N/A	N/A	Cochrane Risk of	Overall low risk of bias, with

												occult blood test		1.12 (1.11 , 1.13)				Bias tool	low risks on all items apart from unclear for blinding of participants and personell
Mullins 2009	RCT	15,000	National screening programme (Australia)	Overdue	65-69	Women	N/A	N/A	Cervical	11 weeks	1. Invitation letter 2. Educational (printed material)	No intervention	Screening uptake	**Events/total= 235/5000 vs. 40/2500**	**Events/total= 215/4999 vs. 80/2500**	N/A	N/A	Cochrane Risk of Bias tool	Unclear risks on all items, apart from low risks for random sequence generation and incomplete outcome data biases
Nadarzynski et al 2012	RCT	606	UK	N/A	21 (mean)	Women	N/A	N/A	Cervical	N/A	Usual care + website delivery of HPV information, which included the same basic information	Usual care only: Basic description of cervical cancer	Risk perception (of cervical cervical cancer and HPV infectio	SMD (95% CIs)= -0.04 (-0.22, 0.14)	N/A	N/A	N/A	Cochrane Risk of Bias tool (version 2)	Overall some concerns, with concerns related to random

											plus a description of the causal role of HPV in cervical cancer + risk factor, which included the basic information plus information about cervical cancer risk factors		n)						isation process and deviations from intended interventions, otherwise low risks of biases
Neter et al 2014	RCT	29,833	Medical insurance records (Israel)	N/A	50-74	N/A	N/A	N/A	Colorectal	6 months	Use of 'implementation intentions': instruction leaflet sent to participants containing suggestions for overcoming common problems that individuals face in attempting to perform a fecal occult blood test, and an encouragement	Standard invitation	Screening uptake	Unadjusted OR (95% CIs)= 1.18 (1.12 , 1.24)	N/A	N/A	N/A	Cochrane Risk of Bias tool	Overall low risk of bias, with low risks on all items apart from unclear for blinding of participants and personell

Ng et al 1998	RCT	1,25,525	Population registry (Singapore)	No screening in the past 1 year or biopsy within 6 months	50-64	Women	N/A	N/A	Breast	2 years	Letter invitation for a free screening; if no reply: 2 follow-up letters	No invitation sent	Stage (0 or 1) of cancer diagnosis	Percentage= 64% of 28231 vs. 26% of 97294	N/A	N/A	N/A	Critical appraisal instruments from Joanna Briggs Institute for RCTs	Total score 7, with 'yes' for blinding of participants, identical treatment of groups, complete follow-up, analysis of participants in groups to which they were randomly assigned, same measurements for groups, reliable measures and appropriate trial design, otherwi

																			se unclear for all other items
Nichols 1986	RCT	17,824	UK	N/A	N/A	N/A	N/A	N/A	Colorectal	N/A	1. Printed information material (educational booklet) in addition to invitation letter;<br>2. Mailing fecal occult blood test kit to residence	1. Letter only;<br>2. Invitation to pick up kit at the clinic	Screening uptake	**Events/total= 2722/7170 vs. 2854/7227**	**Events/total= Scheduled appointment= 3108/8136 vs. 1809/3698 Open appointment= 3108/8136 vs. 587/2142**	N/A	N/A	Cochrane Risk of Bias tool + CASP criteria	Concerns related to all items apart from selective outcome reporting
O'Carroll et al 2015	RCT	39,762	National screening programme (Scotland)	N/A	50-74	N/A	N/A	N/A	Colorectal	N/A	1. Invitation pack included survey with questions to provoke anticipated regret;<br>2. Invitation pack included a survey including health locus	1. Invitation included survey without questions to provoke anticipated regret;<br>2. Standard invitation pack without	Screening uptake	**Risk ratio (95% CIs)= 1.01 (0.99, 1.03)**	**Risk ratio (95% CIs)= 1.00 (0.98, 1.01)**	N/A	N/A	Cochrane Risk of Bias tool	Overall low risk of bias, with low risks on all items apart from unclear for

											of control questions	survey							blinding of participants and personnel
O'Connor 1998	RCT	468	East London general practice (England)	N/A	N/A	Women	N/A	N/A	Breast	N/A	GP letter + explanatory leaflet + invitation from NHS breast screening programme	Invitation from NHS breast screening programme only	Screening uptake	Events/total= 134/ 236 vs. 120/ 234	N/A	N/A	N/A	Cochrane Risk of Bias tool + CASP criteria	Low risks of allocation, incomplete outcome data, baseline comparability and selective outcome reporting biases, high for blinding of assessors and intention to intervene

Page et al 2006	RCT	3,144	Breast Screen New South Wales (Australia)	On electoral roll during March 2004 who had never attended Breast Screen New South Wales for a mammogramy in two Screening and Assessment Service catchment areas (Australia)	50-54	Women	98.6% White	All SES	Breast	12 weeks	1. Mail recall/reminder in addition to invitation letter; 2. Phone call reminder in addition to invitation letter	Invitation letter	Screening uptake	Events/total= 402/669 vs. 304/658	Events/total= 61/785 vs. 43/786	N/A	N/A	Cochrane Risk of Bias tool + CASP criteria	Low risks of biases related to sequence generation, selective outcome reporting, pre-selected population and intention-to-treat analaysis inclusion, high risks for concealment, blinding of assessor and incomplete outcome data biases
Perestelo-Perez et al 2019	RCT	107	Spain	No previous screening	50-69	N/A	N/A	N/A	Colorectal	N/A	Web-based decision aid that was reviewed in	No intervention	Informed choice	Informed choice	N/A	N/A	N/A	Cochrane Risk of	Unclear risk of bias due to

											the company of a researcher that included clinical information, probabilities of outcomes, explicit values clarification, guidance in decision-making and summary document including content explored and participant responses regarding their preferences			Risk ratio (95% CIs)= 45.61 (6.35, 327.38)				Bias tool	blinding of participants and personnel (performance bias), selective reporting (reporting bias) and 'other bias'
Pérez-Lacasta 2019	RCT (cluster)	524	Spain	N/A	50.14 (intervention mean); 50.19 (control mean)	Women	N/A	N/A	Breast	N/A	Received a decision aid that was a leaflet with detailed information on the benefits and harms of screening. The decision aid provided information about options and associated benefits/harms and helped clarify	Received a standard leaflet that did not mention harms and recommended accepting the invitation to participate in the biennial exams of the breast cancer screening programme	Knowledge, informed choice, confidence, anxiety	Knowledge SMD (95% CIs)= 1.87 (1.47, 2.27) Informed choice Risk ratio (95% CIs)= 24.30	N/A	N/A	N/A	Cochrane Risk of Bias tool (version 2)	Overall high risk of bias, due to potential deviation from intended interventions, missing outcome data and the measur

											congruence between decisions and personal values	(usual care)		(3.35 , 176.08) Confidence SMD (95% CIs)= -0.41 (-0.74 , -0.07) Anxiety SMD (95% CIs)= 0.09 (-0.25 , 0.42)					ement of the outcome
Piana 2011	RCT	9,334	Bouches-du-Rhône (France)	Did not respond to invitation for conventional screening and had not had a cervical smear in > 2 years	35-69	Women	N/A	N/A	Cervical	N/A	Direct mailing of self-sampling kit, preceded by a notification with an opt-out option	Invitation for conventional cytology	Screening uptake	Events/total= 939/4400 vs. 311/4934	N/A	N/A	N/A	Cochrane Risk of Bias tool	Low risks of random sequence generation and incomplete outcome data biases, moderate for allocation concealment

																			and reporting of timelines, high for selective reporting
Pierce 1989	RCT	416	General practice (UK)	Due	N/A	Women	N/A	N/A	Cervical	1 year	Letter asking women to have a smear	No intervention	Screening uptake	Events/total= 45/140 vs. 20/134	N/A	N/A	N/A	Cochrane Risk of Bias tool	Low risk of incomplete outcome data bias, otherwise unclear for all other items
Pignone 2013	RCT	911	Australia	N/A	50-70	Men	White and 'Non-white'	N/A	Prostate	N/A	Online rating and ranking task and discrete choice experiments (explicit value clarification)	Balance sheet task (implicit value clarification)	Intention to screen	Events/total= 458/609 vs. 233/302	N/A	N/A	N/A	Cochrane Risk of Bias tool	Unclear risks of sequence generation, allocation concealment and blinding of participants biases, otherwise low risks

Pritchard 1995	RCT	757	A university general practice in Perth (Australia)	Due	36-69	Women	N/A	Socioeconomically disadvantaged area	Cervical	1 year	1. Letter with open invitation to make appointment; 2. Letter with fixed appointment	Usual care	Screening uptake	Events/total= 27/103 vs. 16/93	Events/total= 26/84 vs. 16/93	N/A	N/A	Cochrane Risk of Bias tool	Low risks of random sequence generation and incomplete outcome data biases, otherwise unclear risks on all items
Qureshi 2021	RCT	10,810	Community venues near Oslo (Norway)	Non-exposed'	20-69	Women	Pakistani and Somali	N/A	Cervical	6 months	Educational session delivered in participants' languages	No intervention	Screening uptake	Risk ratio (95% CIs)= 1.11 (1.05, 1.17)	N/A	N/A	N/A	Effective Public Health Care Practice Project (EPHPP) quantitative study quality assessment tool	Rated as weak quality

Racey 2016	RCT	818	Rural areas - opportunistic screening within primary care in Ontario (Canada)	Overdue/underscreened	30-70	Women	N/A	N/A	Cervical	N/A	1. Mailed HPV self-collected test<br>2. Invitation letter for Pap test	No intervention - opportunistic screening	Screening uptake	Risk ratio (95% CIs)= 3.7 (2.2, 6.4)	Events/total= 51/331 vs. 13/152	N/A	N/A	Cochrane Risk of Bias tool	Low risks of random sequence generation and incomplete outcome data biases, high risk of other bias, otherwise unclear risks on all items
Radde et al 2016	RCT	5,265	Population registry, Mainz communities (Germany)	N/A	30-65	Women	N/A	N/A	Cervical	3 years	1. Invitation letter for Pap test<br>2. Invitation letter for Pap test and information brochure	No invitation	Screening uptake	Events/total= 1911/2645 vs. 753/1241	Events/total= 1843/2630 vs. 753/1242	N/A	N/A	Cochrane Risk of Bias tool	Low risk of random sequence generation bias, high risk of incomplete outcome data bias, otherwise unclear risks on all

																			items
Raine et al 2016	RCT	1,67,741	General practices (England)	Eligible for screening	60-74	N/A	N/A	Aimed to reduced socioeconomic gradient in uptake	Colorectal	N/A	Reminder letter with added banner and text	Standard letter	Screening uptake	Adjusted ORs (95% CIs)= 1.07 (1.03, 1.11)	N/A	N/A	N/A	Cochrane Risk of Bias tool	Overall low risk of bias, with low risks on all items
Rat et al 2014	RCT	173	France	At increased melanoma risk	43.2 (mean)	76% women	N/A	N/A	Skin	5 months	Primary care physician counselling using tailored feedback	No intervention	Skin examination (self-reported)	**Events/total= 51/97 vs. 28/76**	N/A	N/A	N/A	United States Preventive Services Task Force Procedure Manual	Rated as 'fair' quality (failed to meet at least 1 criterion but had no known issue that would invalidate its results)
Rat et al 2017	RCT (cluster)	951	National (France)	Previously unresponsive	60.9 (mean)	38.9% women	N/A	N/A	Colorectal	1 year	Provider alerts	No intervention	Screening uptake	Events/total= 123/496 vs. 94/455	N/A	N/A	N/A	Cochrane Risk of Bias tool	Overall low risk of bias, with low risks on all items apart from unclear for allocati

																			on concealment and blinding of participants
Reder 2017	RCT	1,206	Germany	N/A	Those aged 50 included	Women	N/A	N/A	Breast	N/A	Online decision aid that consisted of a static information part and an interactive part. In the information part, the chance of each outcome was expressed as event rate per 200 women screened every 2 years for 20 years using absolute numbers accompanied by crowd figure. The advantages and disadvantages of the mammography screening programme and their probabilities	Received a booklet containing standard information, quality of the screening, breast cancer and its risk factors, procedure, interval cancers and symptoms, follow-up diagnoses, advantages and disadvantages of screening	Knowledge, decisional conflict, informed choice	Knowledge SMD (95% CIs)= 0.30 (0.15 , 0.44) Decisional conflict SMD (95% CIs)= -0.21 (-0.36 , -0.06) Informed choice Risk ratio (95% CIs)= 1.31 (1.04 , 1.65)	N/A	N/A	N/A	Cochrane Risk of Bias tool (version 2)	High risk of bias, including for missing outcome data and measurement of the outcome

											were described. The interactive part of the DA summarised the main points of the information part and encouraged engagement with the information								
Richardson 1994	RCT	890	Population-based screening programme (New Zealand)	N/A	50-64	Women	N/A	N/A	Breast	N/A	1. Invitation letter from GP; if no reply within 2 weeks a postal reminder was sent from the screening centre;<br>2. Telephone reminder (up to 3 calls made at different times of day)	1. Did not receive a letter with the invitation;<br>2. Postal reminder	Screening uptake	Events/total= 113/203 vs. 82/192	Events/total= 118/248 vs. 121/247	N/A	N/A	Cochrane Risk of Bias tool + CASP criteria	Low risks of biases related to baseline comparability, incomplete outcome data and selective outcome reporting, concerns related to allocation and blinding of

																			assessors, high risk of bias related to intention to intervene
Ritvo et al 2015	RCT	5,240	Health centre (Canada)	50-74	N/A	N/A	N/A	N/A	Colorectal	1 year	1. Patient navigation (face-to-face and telephone). This included colorectal cancer screening education, nurses eliciting participants' preferences of screening type and providing appropriate access;<br>2. Faecal occult blood tests (instead of conventional colonoscopy)	Usual care	Screening uptake	Events/total= 458/2629 vs. 214/2611	Events/total= 465/2629 vs. 319/2611	N/A	N/A	Cochrane Risk of Bias tool (version 2)	Overall some concerns, with low risks of bias on all items apart from some concerns related to effect of assignment to intervention

Roberto 2020	RCT	2,119	Regional organised programmes (Italy)	N/A	49.7 (mean)	Women	97.4% Italian (intervention); 96.2% Italian (control)	N/A	Breast	N/A	Online decision aid, non-static, with 19 screens, each covering 1 topic and answering a question. This included short coloured text, figures, bullet points and hyperlinks. The DA homepage used a nudging-like approach to highlight the 4 main sections: What is breast cancer?; What is mammography screening?; What are its benefits and harms?; and What results can be expected from mammography screening? The aid allowed women to decide which sections to access first,	Standard brochure that combined the best information from participating centres' brochures. The brochure was an online static web page divided into 4 sections. This included black-and-white text and no figures. Absolute numbers were reported about breast cancer	Decisional conflict, informed choice	Decisional conflict Risk ratio (95% CIs)= 0.75 (0.56, 0.99) Informed choice Risk ratio (95% CIs)= 1.19 (1.02, 1.38)	N/A	N/A	N/A	Cochrane Risk of Bias tool (version 2)	High risk of bias, including for missing outcome data and measurement of the outcome

											and move to other pages linked from the homepage. The aid provided a list of issues and concerns that possibly affect the screening decision. Each woman was asked to state the importance of each of these items								
Robinson et al 1994	RCT	153	One GP practice's records (UK)	N/A	50-74	N/A	N/A	N/A	Colorectal	N/A	1. Instructions do not include dietary restrictions; 2. Testing completed over 6 days	1. Instructions do include dietary restrictions; 2. Testing completed over 3 days	Screening uptake	Risk ratio (95% CIs)= 1.42 (1.09, 1.83)	Risk ratio (95% CIs)= 1.02 (0.80, 1.31)	N/A	N/A	Cochrane Risk of Bias tool	Overall unclear risk of bias, with unclear risks for random sequence generation, allocation concealment, blinding of participants and personnel and selectiv

																			e reporting, otherwise low
Robson 1989	RCT	1,605	Registered with a general practice in inner London (England)	Due	30-65	Women and men	N/A	N/A	Cervical	2 years	Patients had open access to a health promotion nurse and had their risk factors assessed and followed up by both their GP and the nurse	Usual care (i.e. managed by GP alone)	Screening uptake	Events/total= 606/799 vs. 392/608	N/A	N/A	N/A	Cochrane Risk of Bias tool	Low risks of random sequence generation and incomplete outcome data biases, high risk of other bias, otherwise unclear risks on all items
Roshanai 2009	RCT	163	Cancer genetic clinic of Uppsala University Hospital (Sweden)	N/A	N/A	Women	N/A	N/A	Breast	N/A	Standard genetic counselling + nurse consultation	Standard genetic counselling alone	Risk accuracy	Events/total= ≤ 3 months= 28/73 vs. 24/74 >3 months= 18/68 vs.	N/A	N/A	N/A	Cochrane Risk of Bias tool	Low risks of biases related to blinding of participants, personnel and outcome assessors,

														17/71					unclear risks of random sequence generation, allocation concealment, incomplete outcome data, selective reporting and other biases
Ruparel et al 2019	RCT (nested)	229	Primary care records and invited to a hospital setting for the Lung Cancer Screen Uptake Trial (subset of participants) (UK)	Smokers and former smokers (within 5 years of quitting)	60-75	N/A	N/A	N/A	Lung	N/A	5.5-minute information film and 10-page booklet. Participants given 10 minutes to watch film and/or read booklet. Film and booklet discussed lung cancer, benefits and harms of screening, low dose computer tomography procedure, and possible	Booklet only	Screening uptake, knowledge, decisional conflict	Percentage Screening uptake= 76.7% of 126 vs. 78.9% of 120 Mean increase (SD) Objective	N/A	N/A	N/A	Mixed Methods Appraisal Tool	Assessed as 'yes' on all items, apart from 'no' to whether outcome assessors were blinded to the intervention

		results from the scan	knowledge = 2.16 (1.8) (n=120) vs. 1.84 (1.9) (n=109) Mean increase (SD) Subjective knowledge = 0.92 (1.0) (n=120) vs. 0.55 (1.0) (n=109) Mean (SD) Decisional conflict= 8.5 (1.3) (n=120)	

														vs. 8.2 (1.5) (n=109)					
Sancho-Garnier 2013	RCT	18,730	France	Had not responded to invitation for conventional screening and had not had a cervical smear in > 2 years	35-69	Women	N/A	N/A	Cervical	N/A	Direct mailing of self-sampling kit, preceded by a notification	Invitation for conventional cytology at an outpatient clinic. The invitation included a list of centres performing the test	Screening uptake	Per protocol participation difference (fractions) between intervention & control (with 95% CIs)= 0.16 (0.15, 0.17)	N/A	N/A	N/A	Cochrane Risk of Bias tool	Low risks of incomplete outcome data and reporting of timelines biases, otherwise unclear risks
Sandiford et al 2019	RCT	7,601	Nationwide (New Zealand)	N/A	N/A	N/A	Maoris, Pacific-Islanders	N/A	Colorectal	N/A	Active telephone follow-up and advice on logistical barriers by ethnically aligned callers over 4 weeks, where	Reminder letter only	Screening uptake	Imputed gain (95% CIs)= Maori= 5.2% (1.8, 8.5)	N/A	N/A	N/A	Cochrane Risk of Bias tool (version 2)	Overall low risk of bias, with low risks on all items

											possible linguistically aligned			Pacific-Islander= 3.6% (0.7, 6.4)					
Santare et al 2015	RCT	15,000	Population, screening and cancer registries (Latvia)	N/A	50-74	N/A	N/A	N/A	Colorectal	N/A	1. Instructions do not include dietary restrictions (Fecal immunochemical test); 2. Kit includes round test tube with two caps (OC Sensor); 3. Advanced notification letter two weeks prior to receiving standard invitation pack	1. Instructions do include dietary restrictions (Guaiac-based faecal occult blood test); 2. Kit includes flat test tube with one cap (FOB Gold); 3. Standard invitation pack only	Screening uptake	Risk ratio (95% CIs)= 1.48 (1.41, 1.55)	Risk ratio (95% CIs)= 1.06 (1.02, 1.11)	Risk ratio (95% CIs)= 1.08 (1.04, 1.12)	N/A	Cochrane Risk of Bias tool	Overall unclear risk of bias, with unclear for blinding and selective reporting, otherwise low risks
Segnan 1998	RCT	16,454	Italy	N/A	N/A	Women	N/A	N/A	Breast, Cervical	N/A	1. Letter from GP; 2. Scheduled appointment	1. Letter 2. Open appointment	Screening uptake	**Events/total= Breast= 945/2013 vs. 837/2015 Cervical= 759/2100 vs.**	**Events/total= Breast= 945/2013 vs. 683/2016 Cervical= 759/2100 vs.**	N/A	N/A	Cochrane Risk of Bias tool + CASP criteria	High risk related to analysis of cluster RCTs, some concerns related to blinding of

PRISMA 2020 Checklist

														**647/ 2094**	**474/ 2093**				assessors, otherwise low risks
Segura 2001	RCT (cluster)	2,958	"Raval Nord" neighborhood of the city of Barcelona (Spain)	N/A	50-64	Women	N/A	Positive effect appeared restricted to women with lower educational levels	Breast	2 months	1. Letters sent by mail from the Primary Health Care Team;<br>2. Direct contact through a trained professional	Letters sent by mail from the program	Screening uptake	Events/total= 174/ 313 vs. 157/ 302	Events/total= 216/ 340 vs. 165/ 317	N/A	N/A	Cochrane Risk of Bias tool + CASP criteria	High risks of blinding of assessor, incomplete outcome data, intention-to-treat and cRTC analysis biases, otherwise low
Selva et al 2019	RCT	512	Catalonia (Spain)	N/A	50-69	Men and women	N/A	N/A	Colorectal	N/A	Non-tailored telephone education. Duration of calls: 5.25 min	Usual care: mailed fecal occult blood test kit with printed reminders	Screening uptake	Events/total= 122/ 256 vs. 102/ 256	N/A	N/A	N/A	Cochrane Risk of Bias tool	Low risks on all items, apart from high risk for blinding of participants and personnel bias
Senore et al 2015	RCT	20,701	National (Italy)	N/A	N/A	53.1% women	N/A	N/A	Colorectal	9 months	Pre-Fecal immunochemical test	No intervention	Screening uptake	Events/total=	N/A	N/A	N/A	Cochrane Risk	Overall high risk of

											advance notification			3888/10257 vs. 3580/10444				of Bias tool	bias, with unclear blinding and selective reporting biases, otherwise low
Seow 1998	RCT	1,428	Singapore	Had not responded	N/A	Women	N/A	N/A	Breast	N/A	1. Same letter as control but with a family information pack designed to address the most significant barriers to mammography; 2. Additional home visit to make contact with the woman and her family	Routine second reminder letter (with a screening date) sent through the mail	Screening uptake	Events/total= 38/500 vs. 35/500	Events/total= 57/428 vs. 35/500	N/A	N/A	Cochrane Risk of Bias tool + CASP criteria	High risk related to intention to intervene, otherwise low risks
Shankleman et al 2014	RCT	9,113	General practices in East London (England)	Eligible for screening	59-70	N/A	Health promotion delivered by phone among ethnic diversity populations	Health promotion delivered by phone among low socioeconomic status populations	Colorectal	8 months	1. Health promotion over the telephone: received standard invitation plus bi-lingual advocates phoned subjects a week after sending	Control GP practices: received usual care (i.e. NHS bowel cancer screening programme (BCSP) invitation)	Screening uptake	Median uptake= 46.7% of 2034 vs. 39.1% of 5227	Median uptake= 43.8% of 1852 vs. 39.1% of 5228	N/A	N/A	Cochrane Risk of Bias tool	Overall high risk of bias, with high risk for incomplete outcome data bias, unclear

											letters (3 attempts in total) plus callers offered colorectal cancer information and answered participants' questions;<br>2. Face-to-face health promotion: received standard invitation letter plus an invitation to attend a group health information session at the GP practice, which were held monthly and attendees were offered additional pictorial and multi-lingual guides to the test procedure								risks for random sequence generation, allocation concealment, blinding of participants and personnel and blinding of outcome assessment biases, low risk for selective reporting bias and inclusion of intention-to-treat analysis
Sharp 1996	RCT	782	UK	N/A	N/A	N/A	N/A	N/A	Breast	N/A	1. Nurse delivered home interview with a patient-	Letter from GP	Screening uptake	Events/total= 36/315	N/A	N/A	N/A	Cochrane Risk of Bias	High risk related to intentio

											specific health education component			vs. 21/160				tool + CASP criteria	n to intervene, concerns related to baseline comparability, otherwise low risks
Smith et al 2010	RCT	530	Community-based (Australia)	Average risk of colorectal cancer or slightly above	55-64	N/A	N/A	Socioeconomically disadvantaged areas	Colorectal	N/A	Paper-based decision aid with DVD with and without question prompt list	Standard national information booklet	Multiple	Absolute difference (95% CIs) Screening uptake= -16 (-24, -8) Mean (SD) Knowledge= 54.17 (27.83) (n=357) vs. 34.17 (14.25)	N/A	N/A	N/A	Cochrane Risk of Bias tool	Low risks on all items

(n=173)
Absolute difference (95% CIs)
Attitudes = -14 (-23, -5)
Absolute difference (95% CIs)
Informed choice= 22 (15, 29)
Absolute difference (95% CIs)
Decisional conflict= -13 (-22, -4)

Stamatiou 2008	RCT	1,135	Clinic, university-based (Greece)	N/A	50-86	Men	N/A	N/A	Prostate	2 years	Regular recommendation by physician + additional educational leaflet pre-consultation	Regular recommendation by physician only	Screening uptake	Events/total= 442/548 vs. 227/587	N/A	N/A	N/A	Cochrane Risk of Bias tool	High risks for blinding of personnel and incomplete outcome data biases, low risk for selective outcome reporting, otherwise unclear risks
Steadman 2004	RCT	76	Community, non-psychology undergraduates (UK)	N/A	18-35	Men	N/A	N/A	Prostate	3 weeks	Implementation intention requiring participant to decide when and where they would perform testicular self-examination in the next 3 weeks	Usual care	Screening uptake	Events/total= 30/46 vs. 12/30	N/A	N/A	N/A	Cochrane Risk of Bias tool	High risks for incomplete outcome data and other biases, unclear for allocation concealment, blinding of participants

																			and blinding of assessor, otherwise low
Steckelberg 2011	RCT	1,577	Germany	No screening history	N/A	N/A	N/A	N/A	Colorectal	N/A	Brochure on options' outcomes, clinical problem, and outcome probabilities	Usual care using pamphlet	Multiple	Events/total Screening uptake= 141/785 vs. 134/792 Mean (SD) Knowledge= 53.8 (28.8) (n=785) vs. 31.3 (15) (n=792) Events/total Accurate risk perc	N/A	N/A	N/A	Cochrane Risk of Bias tool	Low risks on all items, apart from unclear risk of other bias

														eptions= 361/785 vs. 141/792 Events/total Informed values-choice congruence= 345/785 vs. 101/792					
Stein et al 2005	RCT	1,140	Community (Devon, England)	Overdue (no record of screening in past 15 years)	39-64	Women	N/A	N/A	Cervical	3 months	1. Telephone call. Telephone call from experienced research nurse using a prepared script. Maxiumum of three attempts were made on consecutive days; 2. Letter from Health Authority	No Intervention	Screening uptake	**Events/total= 4/111 vs. 1/95**	**Events/total= 13/219 vs. 1/95**	**Events/total= 5/221 vs. 1/95**	N/A	Cochrane Risk of Bias tool	Low risk for random sequence generation bias, high for incomplete outcome data, otherwise unclear

											District Cervical Screening Commisioner on behalf of National Cervical Screening Programme; 3. Letter from a well known journalist and broadcaster (Claire Rayner) who was also Chair of the Patients Association							
Stephens et al 2007	RCT (cluster)	91	Adelaide (South Australia)	1st-degree relatives	> 18 years	57% women	N/A	N/A	Colorectal	3 months	Information pamphlet sent to patient's relatives 1 week after operation regarding colorectal cancer risks, benefits to screening, cues and barriers	Information provided to index patients by the treating surgeon regarding risk associated with family colorectal cancer	Screening uptake	Percentage= 6% of 32 vs. 8% of 59	N/A	N/A	N/A	Cochrane Risk of Bias tool (adaption)	Target sample size not achieved, otherwise low risks
Stoffel et al 2019	RCT	N/A	Spain	N/A	N/A	N/A	N/A	N/A	Colorectal	N/A	Invitation letters advocating social norms of undergoing screening, that a high proportion of individuals	Standard invitation letter	Screening uptake	Unadjusted OR (95% CIs)= 1.00 (0.85, 1.18)	N/A	N/A	N/A	Modified Jadad scale	Rated as moderate quality

											are already participating								
Stoffel et al 2021	RCT	N/A	Cyprus	N/A	N/A	N/A	N/A	N/A	Colorectal	Eigth weeks	Invitation letters advocating social norms of undergoing screening, that a high proportion of individuals are already participating	Standard invitation letter	Screening uptake	Unadjusted OR (95% CIs)= 1.01 (0.73, 1.41)	N/A	N/A	N/A	Modified Jadad scale	Rated as high quality
Sultana 2016	RCT	8,160	Victorian residents (Australia)	Never or underscreened (not screened in past 5 years)	30-69	Women	N/A	N/A	Cervical	6 months	Sent a pre-invitation letter to receive a self-sampling kit. The second letter was sent three weeks after the first, including an information brochure on HPV and cervical cancer, the collection device with user instructions, an information form and a postage paid envelope for returning the sample and form	Received a single invitation letter (never-screened) or a standard reminder letter (under-screened) to have a Pap test. A Pap test brochure, form and pre-paid envelope similar to the intervention arm were included in the letter	Screening uptake	Events/total= 2270/7140 vs. 126/1020	N/A	N/A	N/A	Cochrane Risk of Bias tool	Low risks on all items, apart from unclear risk of other bias

Szarewski 2011	RCT	6,000	UK	Did not attend for conventional cervical screening	N/A	Women	N/A	N/A	Cervical	N/A	Mailing of a self-sampling device for HPV testing	Recall for Pap test at clinic in non-responders	Screening uptake	Events/total= 153/1500 vs. 68/1500	N/A	N/A	N/A	Cochrane Risk of Bias tool	Medium risks for selection biases, otherwise low
Thomas 2014	RCT	26	Community (Australia)	No history of prostate cancer	50-70	Men	N/A	N/A	Prostate	N/A	Received prostate-specific antigen fact sheet + attended 2-day community jury where three experts discussed the benefits, harm and general information about prostate-specific antigen testing, with discussion between participants and experts	Received prostate-specific antigen fact sheet only	Intention to screen	Events/total= 5/12 vs. 11/14	N/A	N/A	N/A	Cochrane Risk of Bias tool	High risk of blinding of participants bias, unclear for blinding of personnel, otherwise low risks
Tinmouth et al 2015	RCT	3,594	Canada	N/A	N/A	N/A	N/A	N/A	Colorectal	6 months	Addition of a guaiac-based fecal occult blood test kit to a second mailed invitation	Standard invitation	Screening uptake	Unadjusted OR (95% CIs)= 2.35 (1.93 , 2.90)	N/A	N/A	N/A	Self-modified	Low risks for attrition and reporting biases, moderate for selectio

																			n and performance and detection biases
Tran 2015	RCT	1,170	From 86 general practice clinics (France)	N/A	50-75	Men	N/A	N/A	Prostate	4 months	Visual decision aid	Usual care (physicians answered patients' questions)	Screening intention	**Events/total= 331/586 vs. 432/578**	N/A	N/A	N/A	Cochrane Risk of Bias tool	Overall unclear risk of bias, with unclear risks for blinding and selective outcome reporting biases, otherwise low
Tranberg 2018	RCT	9,791	Screening programme (Denmark)	Did not reply to a first invitation and were due to a second reminder	30-64	Women	N/A	N/A	Cervical	6 months	1. Reminder mailing with self-sampling kit;<br>2. Reminder mailing with self-sampling to be ordered (opt-in). Women were also offered the possibility to contact a GP for collection of cytology specimen. For	Reminder mailing inviting women to have a cytology specimen taken by a clinician	Screening uptake	**Per protocol participation difference (fractions) between intervention & control**	**Per protocol participation difference (fractions) between intervention & control**	N/A	N/A	Cochrane Risk of Bias tool	Low risks of bias on all items

											both arms: reminder letter if response after 4 months			**(with 95% CIs)= -0.06 (-0.08, -0.04)**	**(with 95% CIs)= -0.17 (-0.19, -0.15)**				
Trevena et al 2008	RCT	314	Australia	Not previously screened	N/A	N/A	N/A	N/A	Colorectal	N/A	Age-gender-family history specific DA booklet with information on options, outcome probabilities, explicit values clarification, guidance (personal worksheet with steps in decision making) (Theory of planned behaviour)	Usual care by consumer guidelines recommending faecal occult blood testing	Multiple	Events/total Screening uptake= 117/134 vs. 124/137 Events/total Knowledge (positive score)= 28/134 vs. 8/137 Events/total Informed values-	N/A	N/A	N/A	Cochrane Risk of Bias tool	Unclear risks of blinding of participants and personnel and incomplete outcome data biases, otherwise low

														choice congruence= 14/134 vs. 2/137					
van Roon et al 2011	RCT	5,000	Municipal database (Netherlands)	N/A	50-74	N/A	N/A	N/A	Colorectal	8 months	Use of a mailed advanced notification letter two weeks prior to receiving standard invitation pack	Standard invitation pack only	Screening uptake	Unadjusted OR (95% CIs)= 1.20 (1.07, 1.34)	N/A	N/A	N/A	Cochrane Risk of Bias tool	Overall unclear risk of bias, with unclear for blinding of participants and personnel, incomplete outcome data and selective reporting, otherwise low
van Rossum et al 2008	RCT	20,623	Municipal database (Netherlands)	N/A	50-75	N/A	N/A	N/A	Colorectal	N/A	Sample from one bowel motion required, collected in tube (Fecal immunochem	Sample from three bowel motions required collected on cards	Screening uptake	Risk ratio (95% CIs)= 1.28 (1.24,	N/A	N/A	N/A	Cochrane Risk of Bias tool	Overall low risk of bias, with unclear for blindin

											ical test)	(Guaiac-based faecal occult blood test)		1.31)					g of outcome assessment, otherwise low
Verne et al 1993	RCT	1,842	GP practice records (UK)	N/A	40-75	N/A	N/A	N/A	Colorectal	N/A	1. Instructions do not include dietary restrictions; 2. Results of test are self-analysed at home; 3. Stool sample collected using a wipe	1. Instructions do include dietary restrictions; 2. Results posted to laboratory for analysis; 3. Standard stool sample collection from bowl	Screening uptake	Risk ratio (95% CIs)= 1.05 (0.96, 1.15)	Risk ratio (95% CIs)= 1.03 (0.92, 1.15)	Risk ratio (95% CIs)= 1.06 (0.95, 1.18)	N/A	Cochrane Risk of Bias tool	Overall unclear risk of bias, with unclear risks of selection, blinding of outcome assessment, selective reporting and other bias, otherwise low
Vidal et al 2014	RCT	12,786	Southern Barcelona metropolitan area (Spain)	N/A	N/A	N/A	N/A	N/A	Breast	5 months	Text message reminder three days before a scheduled appointment with or without a message, with a new appointment date if	No intervention	Screening uptake	Events/total= 2785/3719 vs. 5893/9067	N/A	N/A	N/A	Cochrane Risk of Bias tool (version 2)	Overall high risk of bias, with some concerns related to bias arising from

											requested								the randomisation process and in selection of the reported result, otherwise low risks
Vinker et al 2002	RCT	2,315	Two primary care clinics (Israel)	N/A	50-75	N/A	N/A	N/A	Colorectal	1 year	1. Physician reminder group;<br>2. Patient reminder: Phone call reminder;<br>3. Patient reminder: Letter reminder	Usual care	Screening uptake	Unadjusted OR (95% CIs)= 12.52 (4.58, 34.17)	Unadjusted OR (95% CIs)= 11.21 (3.98, 31.51)	Unadjusted OR (95% CIs)= 6.99 (2.44, 20.03)	N/A	Cochrane Risk of Bias tool	Overall high risk of bias, with high risks for incomplete outcome data and selective reporting, otherwise unclear risks
Virtanen 2011	RCT	4,160	Espoo municipalities (Finland)	Had not attended a screening after an invitation to screen (non-attenders)	30-60	Women	Immigrants had lowest participation rates in the program	N/A	Cervical	N/A	Received by mail a self-sampling kit, an information letter on the study, an informed consent	Received a new invitation letter with a new appointment for screening. They also	Screening uptake	**Events/total= 337/1130 vs. 795/3030**	N/A	N/A	N/A	Cochrane Risk of Bias tool	Unclear risks of allocation concealment, incomplete outcom

											document and a data sheet on HPV infections and cervical cancer screening	received the same questionnaire as the self-sampling arm							e data and other biases, otherwise low risks
Ward 1991	RCT	204 patients of 16 GPs	General practice in inner metropolitan region of Sydney (Australia)	Due	20-65	Women	N/A	N/A	Cervical	1 month	Maximal intervention: GP advised women of need for smear and offered to perform it immediately; GP attempted to persuade those not consenting during that consultation by exploring barriers and reasons for self-exclusions. If still did not consent, GP advised making an appointment for smear within a week	Minimal intervention: GP advised eligible women of need for smear and offered to perform it immediately. Those not consenting advised to make appointment for smear within a week	Screening uptake	Events/total= 60/89 vs. 52/95	N/A	N/A	N/A	Cochrane Risk of Bias tool	Unclear risks on all items, apart from low for incomplete outcome data
Wardle et al 2003	RCT	2,966	Trial centres to recruit patients registered with GPs in London (England)	A 'harder-to-reach' population eligible for screening	55-64	N/A	N/A	A 'harder-to-reach' population	Colorectal	3 months	Received a mailed psychoeducational intervention (booklet) three weeks before	Received usual care	Screening uptake	Percentage= 53.2% of 1453 vs. 49.9	N/A	N/A	N/A	Cochrane Risk of Bias tool	Overall unclear risk of bias, with unclear risks for random

receiving the usual screening invitation. Booklet educational materials drew on various frameworks; health belief model (HBM), Theory of Planned Behaviour and regret theories to address screening barriers and increase positive expectations	% of 1513	sequence generation, allocation concealment, blinding of participants and personnel, blinding of outcome assessment and incomplete outcome data biases, low risk for selective reporting bias and inclusion of intention-to-treat analysis

Wardle et al 2016	RCT (cluster)	2,65,434	Population database of GP patients (UK)	N/A	60-74	N/A	N/A	Aims to reduce the socioeconomic gradient of uptake	Colorectal	N/A	1. Letter from GP with invitation pack endorsing screening; 2. A narrative leaflet with presenting stories of previous participants sent with the invitation pack; 3. A 'gist' leaflet: Simplified version of the screening information leaflet designed for low literacy and numeracy readers (NB: Four interventions were reported on, but one of these were also cited in Raine et al 2016 and is therefore excluded here)	1. Standard invitation from screening organisation; 2. Standard invitation pack without narrative leaflet; 3. Standard invitation pack with standard information leaflet	Screening uptake	**Adjusted ORs (95% CIs)= 1.07 (1.04, 1.10)**	**Adjusted ORs (95% CIs)= 1.00 (0.96, 1.03)**	**Adjusted ORs (95% CIs)= 1.03 (0.99, 1.06)**	N/A	Cochrane Risk of Bias tool	Overall low risk of bias, with low risks on all items

Watson 2006	RCT	997	UK	N/A	N/A	Men	N/A	N/A	Prostate	N/A	Leaflet on options' outcomes, clinical problem, outcome probability	Usual care	Screening uptake/ Knowledge	Events/total Screening uptake= 119/465 vs. 149/512 Mean (range) Knowledge= 75% (0-100%) (n=468) vs. 25% (0-100%) (n=522)	N/A	N/A	N/A	Cochrane Risk of Bias tool	Low risks of random sequence generation, allocation concealment, blinding of outcome assessment and incomplete outcome data biases, otherwise unclear
Watson et al 2013	RCT	11,579	National screening programme (UK)	N/A	60-74	N/A	N/A	N/A	Colorectal	N/A	Survey with medical and lifestyle questions sent within a few days of invitation pack	Standard information pack without survey	Screening uptake	Risk ratio (95% CIs)= 0.91 (0.88, 0.94)	N/A	N/A	N/A	Cochrane Risk of Bias tool	Overall low risk of bias, with unclear for allocation concealment

																			and blinding of participants and personnel, otherwise low
Watts 2014	RCT	138	Australia	Had at least one first- or second-degree relative with a previous diagnosis of prostate cancer	40-79	Men	N/A	N/A	Prostate	1 year	Tailored online patient decision aid	Non-tailored online educational information	Screening uptake	Events/total= 29/42 vs. 36/48	N/A	N/A	N/A	Cochrane Risk of Bias tool	High risks of random sequence generation, incomplete outcome data and other biases, otherwise low
Wikström 2011	RCT	4,060	Sweden	Non-responder	39-60	Women	N/A	N/A	Cervical	N/A	Mailing of a self-sampling device for HPV testing	Recall for Pap test at clinic in non-responders	Screening uptake	Events/total= 779/2000 vs. 188/2060	N/A	N/A	N/A	Cochrane Risk of Bias tool	Low risks of attrition and reporting biases, otherwise moderate
Williams 1989	RCT	392	UK	N/A	N/A	Women	N/A	N/A	Breast	N/A	Letter specified an appointment and women were asked to	Open-ended letter inviting women to return a	Screening uptake	Events/total= 162/188	N/A	N/A	N/A	Cochrane Risk of Bias	Some concerns related to

											cancel or alter appointments, but not to confirm them	form indicating convenient times; appointment was then sent		vs. 154/204				tool + CASP criteria	allocation and selective outcome reporting biases (and incomplete outcome data), high risk of intention to intervene bias, otherwise low risks
Wilson 1987	RCT	250	Five general practices in the Nottingham Health Authority area (UK)	Due (recorded as never having a smear)	45-65	Women	N/A	N/A	Cervical	3 weeks	Sent an appointment + two reminders	Letter of invitation to make an appointment + two reminders	Screening uptake	Events/total= 56/118 vs. 39/122	N/A	N/A	N/A	Cochrane Risk of Bias tool	Low risks for allocation concealment and incomplete outcome data biases, otherwise unclear
Wilson et al 2015	RCT	3,408	National screening programme	N/A	50-74	N/A	N/A	N/A	Colorectal	3 months	1. Tailored Personalised Decision Support	Received usual care	Screening uptake	Unadjusted OR (95%	Unadjusted OR (95%	N/A	N/A	Cochrane Risk of	Overall high risk of bias,

			(Australia)								group: received health information tailored to their stage of readiness to screen; 2. Non-Tailored Personalised Decision Support group: received an online booklet with information about colorectal cancer screening			CIs)= 0.94 (0.77, 1.15)	CIs)= 0.96 (0.78, 1.17)			Bias tool	with high risks for allocation concealment and blinding of participants and personnel biases, otherwise low
Wong 2021	RCT	402	Community centres in various districts (Hong Kong)	Non-exposed'	25 and above	Women	South Asian	N/A	Cervical	3 months	Multifaceted delivered in participants' languages	No intervention	Screening uptake	**Risk ratio (95% CIs)= 1.86 (1.63, 2.13)**	N/A	N/A	N/A	Effective Public Health Care Practice Project (EPHPP) quantitative study quality	Rated as moderate quality

																		assessment tool	
Youl 2005	RCT	1,322	Registered on the Queensland State Electoral Roll (Australia)	N/A	30-79	Men	N/A	N/A	Skin	N/A	Personalised motivational invitation letter signed by a well-known and popular Australian sportsman + factual mailed brochure containing educational information about melanoma and screening	Only personalised motivational invitation letter signed by a well-known and popular Australian sportsman	Screening uptake	Events/total= 128/661 vs. 122/661	N/A	N/A	N/A	Cochrane Risk of Bias tool	Unclear risks for sequence generation, allocation concealment, blinding of participants and other biases, otherwise low risks on all items
Youl 2015	RCT (parallel)	370	Community dwellers (participants from the Queensland electoral and Medicare rolls, Australia)	N/A	Intervention= 31.6 (mean); control= 31.8 (mean)	67% women	N/A	N/A	Skin	12 months	Personalised text message reminders for improving sun protection habits based on the social cognitive theory, which used a conversational tone (weekly for the first three months and monthly during the	No intervention	Multiple	Unadjusted MD (95% CIs) Sun protection habits index = 3 months= 0.02	N/A	N/A	N/A	Cochrane Risk of Bias tool (version 2)	Overall high risk of bias, with high risk due to bias in measurement of the outcome and some concer

following nine months)	(- 0.07, 0.11) 12 months= 0.13 (0.03 , 0.23) Unadjusted risk ratio (95% CIs) Any skin self-examination in past 3 months= 3 months= 1.13 (0.84 , 1.51) 12 months= 1.22 (0.95 , 1.56) Unadjuste	ns due to deviations from intended interventions, otherwise low risks

														d risk ratio (95% CIs) Whole-body skin self-examination at time of the last skin self-examination= 3 months= 1.02 (0.66, 1.57) 12 months= 1.27 (0.72, 2.25)					
Zajac et al 2010	RCT	1,800	GP records and electoral roll (Australia)	N/A	50+	N/A	N/A	N/A	Colorectal	3 subsequent rounds, but no information of	Letter from GP with invitation pack endorsing screening	Standard invitation letter sent from central screening service	Screening uptake	RR (95% CIs)= Round 2= 1.25 (1.11	N/A	N/A	N/A	Cochrane Risk of Bias tool	Overall high risk of bias, but only high for

their duration	, 1.41) Round 3= 1.18 (1.04, 1.35) Round 4= 1.33 (1.17, 1.51)	other bias and unclear for blinding of outcome assessment, otherwise low risk on all items

STUDY CHARACTERISTICS	SAMPLE									INTERVENTION			OUTCOMES					STUDY QUALITY	
**Citation**	**Study design**	**N (baseline total)**	**Setting/country**	**Screening/risk status (initial)**	**Age (year range or mean)**	**Gender**	**Ethnicity**	**Socioeconomic (IMD)**	**Cancer type(s)**	**Duration (evaluation)**	**Intervention arm(s)**	**Comparator(s)/control(s)**	**Outcome measure(s)**	**Intervention (Arm 1) vs. Comparator OR Effect size**	**Intervention (Arm 2) vs. Comparator OR Effect size**	**Intervention (Arm 3) vs. Comparator OR Effect size**	**Intervention (Arm 4) vs. Comparator OR Effect size**	**Tool used (by review)**	**Reviews' assessments**
Acera et al 2017	RCT (cluster)	N/A, women were recruited from	Primary healthcare services in Barcelona (Spain)	Overdue	30-70	Women	N/A	N/A	Cervical	N/A	1. Personalised letter; 2. Personalised letter with	Routine protocol	Screening uptake	Events/total= 1178/2098 vs. 214/1039	Events/total= 3831/6088 vs. 214/1040	Events/total= 2016/3601 vs. 1178/2099	N/A	Cochrane Risk of Bias tool	Unclear risk of bias on all items apart from low risk of incomplete

		4 different participating centres. Each participating centre was assigned one study arm									telephone reminder; 3. Information leaflet (printed)								outcome data
Albada 2012	RCT	197	Attendance at a genetic counselling clinic (Netherlands)	N/A	N/A	Women	N/A	N/A	Breast	≤ 3 months	Pre-visit educational website (E-info gene)	Usual care (brief standard pre-visit leaflet)	Risk perception	**SMD (95% CIs)= -0.09 (-0.37, 0.19)**	N/A	N/A	N/A	Cochrane Risk of Bias tool	Low selection bias, high risk due to lack of blinding, low risk due to incomplete outcome data, low risk of selective reporting, unclear for other potential biases

Arcas et al 2014	RCT	703	Spain	N/A	50-69	Women	N/A	N/A	Breast	2 months	Invitation letter and text message reminder 2 days before the mammography appointment	N/A	Screening uptake	Events/total= 182/233 vs. 340/470	N/A	N/A	N/A	Cochrane Risk of Bias tool	Overall rated as high risk of bias
Aubin-Auger et al 2014	RCT (cluster)	45 (GPs)	France	N/A	N/A	N/A	N/A	N/A	Colorectal	7 months	Implementation of a training course focused on communication skills among GPs	N/A	Screening uptake	Unadjusted OR (95% CIs)= 1.22 (1.07, 1.41)	N/A	N/A	N/A	Cochrane Risk of Bias tool	Low risk of bias on all items
Bais 2007	RCT	2,624	Netherlands	Did not respond to the invitation for conventional screening and the first reminder (> 6 months)	30-50	Women	N/A	N/A	Cervical	6 months	Direct mailing of cervicovaginal brush self-sampling kit (a telephone helpline	Invitation for conventional cytology with an explanatory letter (a telephone helpline and/or website	Screening uptake	**Per protocol participation difference (%) between interventio**	N/A	N/A	N/A	Cochrane Risk of Bias tool	Moderate risk of allocation concealment bias, otherwise low risk of bias on all items

											and/or website with information was available throughout the study)	with information was available throughout the study)		**n & control (with 95% CIs)= 13.7 (8.7, 18.6)**					
Barthe et al 2015	RCT	3,422	GP practices in Paris (France)	Eligible for screening	50-74	N/A	N/A	N/A	Colorectal	6 months	Standard letter signed by GP inviting patients to visit the GP's office to obtain guaiac-based faecal occult blood test	Standard invitation letter and standard reminder	Screening uptake	Unadjusted OR (95% CIs)= 1.04 (0.83, 1.31)	N/A	N/A	N/A	Cochrane Risk of Bias tool	Overall unclear risk of bias, with unclear risks for random sequence generation, blinding of participants and personnel and blinding of outcome assessment biases, otherwise low risks
Bartholomew et al 2019	RCT	5,271	Auckland (New Zealand)	Non-adherent to initial screening invitation	N/A	N/A	Maori and Pacific residents	N/A	Colorectal	3 months	DVD (6-minute describtion of the import	Usual reminder letter only	Screening uptake	Events/total= 372/2341 vs.	N/A	N/A	N/A	Cochrane Risk of Bias tool	Low risk of bias on all items

											ance of screening, ease of test, nature of return, participants' positive experiences of diagnostic follow-up test) and a reminder letter			628/2883					
Baxter and Barata 2011	RCT	193	Canada	N/A	18.43 (mean)	Women	N/A	N/A	Cervical	6 months	Detailed in-depth information (written/verbal) about sexual transmission of HPV	Written information on HPV but did not describe HPV as an STI	Knowledge (about cervical cancer, HPV infection and HPV vaccination)	SMD (95% CIs)= 1.07 (0.75, 1.39)	N/A	N/A	N/A	Cochrane Risk of Bias tool (version 2)	Overall rated as some concerns: some concerns with randomisation and deviations from intended interventions, otherwise low risk of bias
Beti Thompson 2017	RCT	293	Spain	N/A	43.9 (mean)	Women	N/A	N/A	Cervical	7 months	Web video containing	Usual care	Screening uptake	Risk ratio (95% CIs)=	N/A	N/A	N/A	Cochrane Risk of	Low risk of bias on all items

											information about cervical cancer screening, encouragement to undergo screening and information about low cost clinics where women could go for the screening			1.38 (0.84, 1.54)				Bias tool	
Birkenfeld et al 2011	RCT	16,132	Israel	N/A	N/A	N/A	N/A	A higher socioeconomic status was associated with a higher uptake of screening test	Colorectal	N/A	Faecal immunological test	Faecal occult blood test	Screening uptake	Unadjusted OR (95% CIs)= 0.92 (0.85, 0.99)	N/A	N/A	N/A	Self-modified	Low selection bias, otherwise moderate performance, detection, attrition and reporting biases

Boguradzka et al 2014	RCT	600	A group physician practice of four primary care physicians in the urban areas of Warsaw (Poland)	Eligible for screening	50-65	N/A	N/A	N/A	Colorectal	6 months	Primary care physicians directly recommended screening at the end of health visit. Discussion included information about the benefits of colorectal cancer screening and early treatment plus recommendation to participate in colonoscopy screening plus	Primary care physicians prompted patients to obtain a colorectal cancer information leaflet after a scheduled health visit	Screening uptake	Adjusted OR (95% CIs)= 5.33 (3.55, 8.00)	N/A	N/A	N/A	Cochrane Risk of Bias tool	Overall high risk of bias, with high risk for blinding of outcome assessment bias and no intention-to-treat analysis, otherwise low risks

											information about the procedure plus help with screening arrangements								
Bowman 1995	RCT	342	General practice (Australia)	Overdue	18-70	Women	N/A	N/A	Cervical	6 months	GP signature on reminder letter	Standard letter without signature	Screening uptake	Events/total= 52/178 vs. 26/164	N/A	N/A	N/A	Cochrane Risk of Bias tool	Unclear risk of selection, reporting and other biases, low risk (blinding), high risk (incomplete outcome data)
Brain 2000	RCT	545	2 family cancer clinics in Wales (UK)	N/A	N/A	Women	N/A	N/A	Breast	>3 months	Multidisciplinary genetic assessment	Surgical assessment	Risk perception	SMD (95% CIs)= ≤ 3 months= -0.08 (-0.25, 0.09) >3 months= -0.07 (-0.24,	N/A	N/A	N/A	Cochrane Risk of Bias tool	Low risk on items, but unclear for incomplete outcome data and selective reporting

														0.10)					
Braithwaite 2005	RCT	72	UK	N/A	N/A	Women (with a family history of breast cancer)	N/A	N/A	Breast	≤ 3 months	GRACE (genetic risk assessment in the clinical environment) tool	Standard genetic risk counselling	Risk perception	SMD (95% CIs)= 0.54 (0.06, 1.01)	N/A	N/A	N/A	Cochrane Risk of Bias tool	Low risk (blinding, other potential biases), but unclear for allocation concealment, incomplete outcome data and selective reporting
Brewer 2021	RCT	3,553	GP clinics (New Zealand)	Never- and under-screened (no screening recorded for at least the last 5 years prior to enrolment)	30-69	Women	Maori, Pacific and Asian ethnicities	N/A	Cervical	N/A	1. Direct mailing of self-sampling kit, preceded by a notification; 2. Direct offer of self-sampling kit at their usual general	Invitation for conventional cytology (at a clinic, at an independent service providers, or with a study nurse), preceded by a notification	Screening uptake	**Per protocol participation difference (%) between intervention & control (with 95% CIs)=**	**Per protocol participation difference (%) between intervention & control (with 95% CIs)=**	N/A	N/A	Cochrane Risk of Bias tool	Low risk of bias on all items, apart from moderate risk for selective reporting

											practice, preceded by a notification			**0.11 (0.09, 0.14)**	**0.04 (0.02, 0.05)**				
Broberg 2013	RCT	7,207	71 antenatal health clinics in Western Sweden	Overdue	N/A	Women	N/A	N/A	Cervical	12 months	Telephone call to offer appointment for a smear test (up to 10 attempts made)	Routine care	Screening uptake	Events/total= 718/3207 vs. 422/4000	N/A	N/A	N/A	Cochrane Risk of Bias tool	Low risk of random sequence generation bias, unclear of allocation concealment, blinding, reporting and other biases, high risk of incomplete outcome data
Broc et al 2015	RCT	22,025	France	N/A	50-74	Men and women	N/A	N/A	Colorectal	4 months	Flexible motivational interviews, where counselor gave no information or advice about colorectal	Received reminder mail and fecal occult blood test according to specifications in standard guidelines of national colorectal	Screening uptake	Percentage = 29.5% of 2268 9.2% of 19757	N/A	N/A	N/A	Cochrane Risk of Bias tool	Low risk of reporting, incomplete outcome data and other biases, unclear for random sequence generatio

cancer screening. Based on the motivational interviewing, the counselor: let the participants explore their reasons for being (or not being) screened; tailored the session to the subjects' pace. The mean duration was 7.5 minutes, and the interviews were normall	cancer screening programme	n, allocation concealment and blinding

											y completed in 1 call								
Buehler et al 1997	RCT	441	2 family medicine clinics (1 urban, 1 rural) affiliated with the Memorial University of Newfoundland (Canada)	Due (had not had Pap test in the past 3 years)	18-69	Women	N/A	N/A	Cervical	N/A	Sent an invitation asking them to seek a Pap test followed by a reminder letter 4 weeks later	No letters sent	Screening uptake	**Events/total= 19/178 vs. 13/208**	N/A	N/A	N/A	Cochrane Risk of Bias tool	Low risk of random sequence generation and incomplete outcome data biases, unclear for all other items
Cadman 2015	RCT	6,000	UK	Did not respond to >2 invitations for screening (> 46 weeks)	25-65	Women	N/A	N/A	Cervical	N/A	Direct mailing of self-sampling kit	Invitation for conventional cytology	Screening uptake	Per protocol participation difference (%) between intervention & control	N/A	N/A	N/A	Cochrane Risk of Bias tool	Low risk of bias on all items (blinding N/A)

														(with 95% CIs)= 2.80 (1.40, 4.10)					
Campbell 1997	RCT	411	New South Wales (Australia)	Had not had a cervical (Pap) smear in previous 30 months	N/A	Women	N/A	N/A	Cervical	N/A	Computer generated printed personalised feedback, listing 'risk factor' of not having a smear within past 2 years	General risk information	Screening uptake	Events/total= 52/148 vs. 33/124	N/A	N/A	N/A	Cochrane Risk of Bias tool	Low risk of biases for incomplete outcome data, baseline comparability and measure against contamination, unclear for allocation concealment, blinding, reporting and funding biases, high for random sequence generation
Clouston et al 2014	RCT	N/A, 39 medical clinic clust	Winnipeg, Manitoba (Canada)	N/A	50-70	N/A	N/A	N/A	Colorectal	4 months	Family physicians gave their patients a	Usual care	Screening uptake	**Events/total= 805/2026 663/1837**	N/A	N/A	N/A	Cochrane Risk of Bias tool	Low risk of all biases, apart from unclear for other

		ers with family physicians and their patients eligible for colorectal screening									patient decision aid in the form of a refrigerator magnet, which directed patients to accessing colorectal cancer information and a screening nurse-managed support line and website								biases
Clover et al 1996	RCT	2,329	Australia (larger and smaller towns, results for the former extracted due	N/A	40-69	Women	N/A	N/A	Breast	N/A	1. Mass media promotion; 2. Family physician involvement	Community participation	Screening uptake	Percentage (with 95% CIs)= 34% vs. 51% (10-24); Z = -	Percentage (with 95% CIs)= 68% vs. 51% (10-24); Z = -	N/A	N/A	Quality Assessment Tool for Quantitative Studi	Global methodological quality = 'Strong'

			to relevance)											4.96; p < .001	4.53; p < .01			es	
Cole et al 2003	RCT	1,818	Electoral roll (Australia)	N/A	50-69	N/A	N/A	N/A	Colorectal	N/A	1. Reducing the required stool samples from three to two, combined with a brush application; 2. Instructions do not include dietary restrictions	1. Kits that requires participant to collect three samples using spatula; 2. Instructions do include dietary restrictions	Screening uptake	RR (95% CIs)= 1.47 (1.28, 1.68)	RR (95% CIs)= 1.50 (1.27, 1.76)	N/A	N/A	Cochrane Risk of Bias tool	Overall low, with low on all items
Cole et al 2007	RCT	1,200	Electoral roll (Australia)	N/A	50-74	N/A	N/A	N/A	Colorectal	N/A	1. Photographs and advocacy statements from lay persons within standard invitation	1+2. Standard invitation letter containing colorectal cancer prevention and value and ease of screening; 3. Standard invitation	Screening uptake	**RR (95% CIs)= 0.91 (0.79, 1.05)**	**RR (95% CIs)= 1.02 (0.89, 1.17)**	**RR (95% CIs)= 1.22 (1.08, 1.39)**	N/A	Cochrane Risk of Bias tool	Overall unclear, with all items unclear apart from low on blinding of outcome assessment and selective reporting

											letter; 2. Positively framed information about colorectal cancer risks within standard invitation letter; 3. Advanced notification letter two weeks prior to receiving standard invitation pack	pack only							
Darlin 2013	RCT	1,500	Sweden	Had not had smears for >9 years	32-65	Women	N/A	N/A	Cervical	N/A	Direct mailing of self-sampling kit. After one month, a	Invitation for high-risk HPV testing at an outpatient clinic. The invitation	Screening uptake	**Per protocol participation difference (%)**	N/A	N/A	N/A	Cochrane Risk of Bias tool	Moderate for all items apart from low for incomplete outcome

											reminder including another self-sampling kit was sent to non-responders	included several alternative appointments. A reminder was sent to non-responders		**between intervention & control (with 95% CIs)= 10.50 (7.70, 13.30)**					data
Davidson et al 1999	RCT	100	Family Medical Teaching Centre (Canada)	N/A	50-79	Men	N/A	N/A	Prostate	N/A	Verbal and written information about pros and cons of prostate screening and discussion with doctor at a periodic health examination	Given same information as intervention, but only after second interview	Decisional conflict/ Screening uptake	Mean Difference (95% CIs)= Decisional conflict= -10.44 (-16.10 , -4.77)<br><br>Risk ratio (95% CIs)= Actual screening decision= 1.33 (0.88-2.00)	N/A	N/A	N/A	Cochrane Risk of Bias tool	Overall high risk of bias, with blinding and allocation concealment at high risk but low for sequence generation. Low risk of incomplete outcome data for decisional conflict and screening decision

Decker et al 2013	RCT (cluster)	31,452	Manitoba (Canada)	Never had screening	30-69	Women	N/A	N/A	Cervical	6 months	Invitation letter and brochure mailed to participants. The invitation letter was personally addressed in English and French and stated that the woman had not had a Pap test in at least 5 years, described the benefits of screening, and provided Pap test	Not mailed an invitation letter but given an index date of screening that matched the invitation date	Screening uptake	Events/total= 1010/17068 vs. 441/14384	N/A	N/A	N/A	Cochrane Risk of Bias tool	Low risk of selection biases and incomplete outcome data, unclear for all other items

											locations. Screening availability in all the locations were confirmed to ensure access to screening								
Del Mar 1998	RCT	689	On electoral roll in South Brisbane (Australia)	Due and overdue	18-67	Women	Vietnamese	N/A	Cervical	1 year	Personal letter (in Vietnamese) informing them about screening and its benefits	Did not receive a letter	Screening uptake	Events/total= 36/359 vs. 39/330	N/A	N/A	N/A	Cochrane Risk of Bias tool	Low risk of blinding and incomplete outcome data, unclear for all other items
Denters et al 2013	RCT	10,265	National screening program (Netherlands)	N/A	50-75	N/A	N/A	N/A	Colorectal	N/A	Kit that includes feces collection paper for the toilet bowl	Kit that does not include feces collection paper	Screening uptake	Events/total= 2673/5129 vs. 2694/5136	N/A	N/A	N/A	Cochrane Risk of Bias tool	Overall unclear, with low selection biases and incomplete outcome data, but unclear for all

																			other items
Deutekom et al 2010	RCT	20,623	Municipal records (Netherlands)	N/A	50-75	N/A	N/A	N/A	Colorectal	N/A	Test involves one sample collected with brush (fecal immunochemical test)	Test involves three samples collected with cardboard stick (guaiac-based fecal occult blood test)	Screening uptake	Events/total= 6159/10322 vs. 4839/10301	N/A	N/A	N/A	Cochrane Risk of Bias tool	Overall low, with low on all items apart from unclear for blinding of outcome assessment
Dodd 2019	RCT	N/A	Australia	N/A	N/A	N/A	N/A	N/A	Colorectal	1.5 months	Training course targeted at GPs	Usual care	Screening uptake	Unadjusted OR (95% CIs)= 10.24 (2.90, 36.60)	N/A	N/A	N/A	Downs and Black Checklist	Rated as overall 'good' quality
Dubey et al 2006	RCT	4 clinics (38 physicians) involving 13 preventive health servi	St Michael's Hospital/University of Toronto (Canada)	N/A	N/A	N/A	N/A	N/A	Breast, cervical and colorectal	5 months	Prompt/reminder form with patient gender-specific Preventive Care Checklist Forms © attache	Usual care	Screening uptake	Relative risk ratio (95% CIs)= 1.41 (0.76, 2.61)	Relative risk ratio (95% CIs)= 0.92 (0.83, 1.01)	Relative risk ratio (95% CIs)= 6.69 (1.90, 24.10)	N/A	Cochrane Risk of Bias tool (adaption)	Publication status, funding source, randomisation method reported, follow-up period and intention-to-treat analysis reported, abstracto

		ces									d by clerical staff to charts for: 1. Mammography; 2. Pap test; 3. Fecal occult blood test								rs blinded in pre-intervention period and achieved target sample size, 85-90% statistical power, some potential concerns over differences in baseline chcracteristics
Eaker 2004	RCT	12,240	Uppsala county (Sweden)	Due (no Pap smear within past 3 years)	25-59	Women	N/A	N/A	Cervical	5 months	Modified letter (with educational brochure)	Standard letter (without educational brochure)	Screening uptake	Events/total= 1638/6065 vs. 1566/6092	N/A	N/A	N/A	Cochrane Risk of Bias tool	Low risk of allocation concealment and incomplete outcome data biases, unclear for all other items

PRISMA 2020 Checklist

Elfström 2019	RCT	6,000	Health services (Sweden)	Did not have a screening test on record for at least 10 years, or who had been sent at least 10 annual renewed invitations and who were not blocked from invitations due to hysterectomy or screening program opt-out	33-60	Women	N/A	N/A	Cervical	3 months	1. Direct mailing of self-sampling kit along with an invitation letter and instructions; 2. Opportunity to order a selfsampling kit through an online application	No intervention beyond standard invitation	Screening uptake	**Per protocol participation difference (fractions) between intervention & control (with 95% CIs)= 0.16 (0.14, 0.18)**	**Per protocol participation difference (fractions) between intervention & control (with 95% CIs)= 0.06 (0.05, 0.08)**	N/A	N/A	Cochrane Risk of Bias tool	Low risk of all biases
Emery et al 2019	RCT	551	11 general practices in Perth, Western Australia, and 6 in Melbourne, Victoria (Australia)	N/A	N/A	N/A	N/A	N/A	Lung	12 months	Spirometry was performed then participants were guided through a self-help manual to	Spirometry, brief general discussion on lung health	Knowledge, consultation rates and times	Mean difference (p) Knowledge = 1 month= -0.2 (0.3954) 12 month	N/A	N/A	N/A	Mixed Methods Appraisal Tool	Yes on all items indicating low risk of bias/high quality

“increase the salience and personal relevance of symptoms, improve knowledge of symptoms by introducing chest disease prototypes, reinforce the benefits of early intervention in LC and other chest disease, and sanction early consultation.” (p.2) Action plans were	hs= -0.1 (0.6083) Relative rate (95% CIs) Respiratory consultations= 1.40 (1.08-1.82) Chi-square (p) Time to first consultation= 1.5923 (0.207) Hazard ratio (95% CIs) Time to present for consultatio

then developed and linked to symptom checklist. Coping plans were discussed to address barriers to consultation. Monthly prompts (SMS, emails, postcards, phone calls, and fridge magnets) were tailored to individual preferences.	ns= 0.827 (0.64, 1.07)

Enerly 2016	RCT	3,393	Norwegian Cervical Cancer Screening Programme (Norway)	Non-attenders due to receive a second reminder for screening	25-69	Women	N/A	N/A	Cervical	N/A	Sent an information letter, inviting them to participate in the Self-Sampling (SESAM) study. Self-sampling devices were sent to the participants with user instructions, informed consent form, a prepaid return envelope, and a questionnaire to collect informa	Only sent a second reminder letter according to the Norwegian Cervical Cancer Screening Programme guidelines	Screening uptake	Events/total= 267/800 vs. 601/2593	N/A	N/A	N/A	Cochrane Risk of Bias tool	Low risk on all items, apart from unclear for other bias

										tion on attitudes and ease of self-sampling, and to identify reasons for nonattendance according to recommendations									
Evans 2010	RCT	514	Wales	N/A	N/A	Men	N/A	N/A	Prostate	N/A	Online programme on options' outcomes, clinical problem, outcome probabilities, explicit values clarification, others' opinion, guidance	Usual care	Knowledge, decisional conflict, screening uptake	Mean (n) Knowledge = 4.9 (89) vs. 2.17 (103) Mean (SD) Decisional conflict= 38.1 (24.2) (n=89) vs. 49.6 (24.2) (n=103)	N/A	N/A	N/A	Cochrane Risk of Bias tool	Low risk on all items, apart from unclear for random sequence generation and blinding of participants and personnel

											(interactive computer programme; summary)			Events/total Screening uptake= 4/127 vs. 11/123					
Federici et al 2005	RCT (cluster)	7,332	Italy	N/A	N/A	N/A	N/A	N/A	Colorectal	N/A	Faecal immunological test	Guaiac-based faecal occult blood test	Screening uptake	Unadjusted OR (95% CIs)= 1.29 (1.17, 1.43)	N/A	N/A	N/A	Self-modified	Low reporting bias, otherwise moderate selection, performance, detection and attrition biases
Federici et al 2006	RCT (cluster)	7332 patients (distributed amongst 130 GP for intervention, 13 hospital	Italy	N/A	N/A	N/A	N/A	N/A	Colorectal	45 days	Economic incentive €1,000 ($CAN1.57, 2008 10 31) for GP's participation, €10 for each patient they screene	Usual care (Guaiac and immunochemical faecal occult blood test without financial incentive)	Screening uptake	Relative risk ratio (95% CIs)= 3.40 (3.13, 3.70)	N/A	N/A	N/A	Cochrane Risk of Bias tool (adaption)	Publication status and follow-up period reported, target sample size achieved and 90% statistical power, funding source, randomisation

		for comparison)									d (Guaiac and immunochemical faecal occult blood test)								method, baseline characteristics for GPs, blinding and intention-to-treat analysis not reported
Firmino-Machado et al 2019	RCT	1,220	Portugal	N/A	25-49	Women	N/A	N/A	Cervical	45 days	Automated or customised text messages and phone calls, followed by text message reminders of the appointment	Usual care	Screening uptake	Events/total= 103/202 vs. 70/205	N/A	N/A	N/A	Cochrane Risk of Bias tool (version 2)	Overall rated as unclear risk of bias, but only unclear for randomisation bias and low on all other items
Fry 2003	RCT	373	UK	Referred to the regional clinical genetics department for breast cancer genetic risk	N/A	Women	N/A	N/A	Breast	6 months	Novel community-based service	Standard regional service	Risk perception	Percentage perceived their risk to be moderate or high=	N/A	N/A	N/A	Cochrane Risk of Bias tool	Randomisation sequence and allocation both rated as adequate and other potential risks of

				counselling										4 weeks= 92% (n=129) vs. 92% (n=147) 6 months= 91% (n=123) vs. 92% (n=140)					bias were discussed, but blinding 'not adequately described', incomplete outcome data 'unclearly described' and there was risk of bias due to selective reporting
Fujiwara et al 2015	RCT	1,912	Urban area (Japan)	Had not participated in screening for more than a year ('non-adherent')	20-39	Women	N/A	N/A	Cervical	N/A	1. Received a printed reminder with information on the possible benefits of screening; 2. Received a printed remind	Received a printed reminder with simple information	Screening uptake	Percentage = 11.4% of 622 vs. 4.9% of 650	Percentage = 10.3% of 640 vs. 4.9% of 650	N/A	N/A	Cochrane Risk of Bias tool	Low risk of all biases, apart from unclear for other biases

											er with information on the possible benefits and risk of screening								
Gabel et al 2020	RCT	1,723	National screening programme (Denmark)	N/A	53-74	N/A	N/A	N/A	Colorectal	N/A	Web-based decision aid	Nothing apart from national information pamphlet	Screening uptake, knowledge, attitudes, decisional conflict	Unadjusted OR (95% CIs) Screening uptake= 1.40 (1.16, 1.71) Absolute difference (95% CIs) Knowledge = 0.09 (-0.05, 0.24) Absolute difference (95% CIs)	N/A	N/A	N/A	Critical appraisal instruments from Joanna Briggs Institute for experimental and quasi-experimental studies	Yes on all items apart from blinding

PRISMA 2020 Checklist

														Attitudes= 0.45 (0.00, 0.91) Unadjusted OR (95% CIs) Decisional conflict= -0.11 (-0.20, -0.01)					
Gattell ari 2003	RCT	248	Australia	N/A	N/A	Men	N/A	N/A	Prostate	N/A	Pamphlet on options' outcomes, clinical problem, outcome probability, explicit values clarification	Usual care using brief information on screening test and chances of false-positive results	Screening uptake, knowledge, accurate risk perceptions, decisional conflict	Events/total Screening uptake= 27/106 vs. 25/108 Mean (SD) Knowledge = 50 (18.4) (n=106) vs. 45 (15.9) (n=108)	N/A	N/A	N/A	Cochrane Risk of Bias tool	Low risk of allocation concealment, detection and other bias, unclear for all other items

														Events/total Accurate risk perceptions= 57/106 vs. 11/108 Mean (SD) Decisional conflict= 42.5 (20) (n=106) vs. 42.5 (33.3) (n=108)					
Gattellari 2005	RCT	421	Australia	N/A	N/A	Men	N/A	N/A	Prostate	N/A	Pamphlet on options' outcomes, clinical problem, outcome probability, explicit values	Video on clinical problem, outcome probability, others' opinion, or usual care using brief information on screening test and chances	Screening uptake, knowledge, decisional conflict	Events/total Screening uptake= 37/131 vs. 42/136 Mean (SD) Knowledge	N/A	N/A	N/A	Cochrane Risk of Bias tool	Low risk of bias on all items apart from unclear for selective reporting

											clarification	of false-positive results		= 57.2 (21.3) (n=131) vs. 42.2 (16.7) (n=136) Mean (SD) Decisional conflict= 30.8 (19.3) (n=131) vs. 29.2 (15) (n=136)					
Gimeno-Garcia et al 2009	RCT	158	Spain	N/A	N/A	N/A	N/A	N/A	Colorectal	12 months	Video-based educational intervention + standard letter	Standard letter only	Screening uptake	Unadjusted OR (95% CIs)= 1.91 (0.95, 3.89)	N/A	N/A	N/A	Cochrane Risk of Bias tool	Overall high risk of bias, with unclear selection bias and inadequate/selective reporting, unclear risk of other biases
Giorgi 2000	RCT	8,634	Italy	Non-responders	N/A	Women	N/A	N/A	Breast	N/A	Letter from GP	Standard letter	Screening uptake	Events/total= 1897/	N/A	N/A	N/A	Cochrane Risk of	Unclear sequence generation, high

														2552 vs. 3875/ 6082				Bias tool + CASP criteria	risk of concealment and blinding of assessor biases, otherwise low risks
Giorgi Rossi 2015	RCT	14,041	Organised screening programmes in six local health authorities (Northern Italy)	Had not responded to an earlier screening invitation letter	30-64	Women	N/A	N/A	Cervical	N/A	1. Received self-sampler by mail directly at home. This was preceded by an explanatory letter sent one week earlier; 2. Offered opportunity to pick the self-sampling device up at an area	Received a standard invitation letter to perform either a Pap test or an HPV test at the clinic according to that centre's routine screening	Screening uptake	Percentage = 21.6% of 4516 vs. 11.9% of 5012	Percentage = 12.0% of 4513 vs. 11.9% of 5012	N/A	N/A	Cochrane Risk of Bias tool	Low risk on all items, apart from unclear for other bias

											pharmacy								
Glazebrook et al 2006	RCT	589	Participants at high risk of developing melanoma recruited from Family Practices within Nottinghamshire (UK)	N/A	N/A	N/A	N/A	N/A	Skin	6 months	Multimedia programme called "Skinsafe" with eight sections designed to be completed in 10 to 15 minutes. Included animation, photographs and simple text to inform users about the dangers from excessive sun exposure; how to	Usual care	Screening uptake, knowledge (regarding screening test/condition concerned)	Adjusted OR (95% CIs) Screening uptake= 1.67 (1.04, 2.69) SMD (95% CIs) Knowledge = 0.40 (0.23, 0.56)	N/A	N/A	N/A	Cochrane Risk of Bias tool	Low risk of random sequence generation and other biases, high for selective reporting, unclear on all remaining items

protect skin from the sun; characteristics of skin at risk; early signs of melanoma; how to reduce risk from melanoma; how to check skin for suspicious lesions. The last section was designed to provide individualised feedback on the persons relative risk for skin cancer. Health

											Belief Model was used to base the intervention								
Gold 2011	RCT (parallel)	358	Community dwellers (Australia)	N/A	N/A	39.9% women	N/A	N/A	Skin	4 months	Fortnightly humorous and short text message reminders for improving sun protection habits that used informal language and were linked to particular annual events where possible	Usual care	Behaviour/knowledge	Risk ratio (95% CIs) Consideration of the long-term consequences of prolonged UV exposure= 1.01 (0.84, 1.20)	N/A	N/A	N/A	Cochrane Risk of Bias tool (version 2)	Overall high risk, with high risk of bias due to deviations from intended interventions, otherwise some concerns for all other items
Guiriguet et al	RCT (cluster)	41,042	National (Spain)	No prior screening	58.7 (mean)	53.6% wo	N/A	N/A	Colorectal	1 year	Received an electro	Did not receive a reminder	Screening uptake	Events/total=	N/A	N/A	N/A	Cochrane Risk	Overall low risk, with low

2016	)					men					nic alert reminding patients to discuss screening			9536/21619 vs. 8196/19423				of Bias tool	risk of bias on all items
Gummersbach 2015	RCT	792	Family practices in the federal state of North-Rhine–Westphalia (Germany)	Had not yet received their first invitation to be screened but are just about to receive it	48.67 (intervention mean); 48.76 (control mean)	Women	93.26% native German language (intervention); 89.71% native German language (control)	N/A	Breast	N/A	Edge flyer format leaflet with evidence-based information about the potential benefits and harms of screening. The information included: benefit, mortality reduction, sensitivity, specificity, number	Edge flyer format leaflet that promotes breast cancer screening with limited information about the benefit and sensitivity of mammography screening, the rate of a pathological result of screening, interval cancer, the potential side effects of X-ray	Knowledge, decisional conflict	Knowledge SMD (95% CIs)= 0.13 (-0.09, 0.34) Decisional conflict SMD (95% CIs)= 0.32 (0.11, 0.53)	N/A	N/A	N/A	Cochrane Risk of Bias tool (version 2)	Overall low risk, with low risk of bias on all items

needed to screen, overdiagnosis, false-positive results rate, increase of operation and radiation of women who do not benefit from mammography screening, the rate of a pathological result of screening, interval cancer, and recommended to self-check for

											breast cancer								
Hagoel et al 2016	RCT	48,091	National database (Israel)	No colonoscopy in past 3 years, no fecal occult blood test in previous 1 year	50-74	N/A	N/A	N/A	Colorectal	6 months	Interrogative reminders, with or without reference to social context	Noninterrogative reminders, with or without social context, or no reminders at all	Screening uptake	Adjusted OR (95% CIs)= 1.11 (1.05, 1.19)	N/A	N/A	N/A	Cochrane Risk of Bias tool (version 2)	Overall low risk, with low risk of bias on all items
Haguenoer 2015	RCT	5,998	Regional screening programme (France)	Overdue	30-65	Women	N/A	N/A	Cervical	9 months	1. Recall invitation/reminder for Pap smear (intervention) vs. No intervention (control); 2. Direct mailing of HPV self-samplin	See intervention arms for comparisons	Screening uptake	Events/total= 233/2000 vs. 198/1999	Events/total= 450/1999 vs. 233/2000	N/A	N/A	Cochrane Risk of Bias tool	Low risk of selection and incomplete outcome data biases, unclear for all other items

											g kit (intervention) vs. Recall invitation for Pap smear (control)								
Hegenscheid 2011	RCT	10,954	Germany	Non-responders	50-69	Women	N/A	N/A	Breast	N/A	Phone call reminder in addition to invitation letter	Invitation letter only	Screening uptake	Events/total= 728/2455 vs. 770/2952	N/A	N/A	N/A	Cochrane Risk of Bias tool + CASP criteria	High risk of concealment bias, some concerns regarding blinding of assessor, otherwise low risks on all items
Heranney 2011	RCT	10,662	Organised screening (France)	Overdue	N/A	Women	N/A	N/A	Cervical	8 months	Telephone reminder that Pap smears were necessary. Ten attempts were made to contact	Letter invitation for a Pap smear	Screening uptake	Events/total= 335/5310 vs. 309/5352	N/A	N/A	N/A	Cochrane Risk of Bias tool	Low risk of incomplete outcome data, but otherwise unclear risks on all items
Heverin 2011	RCT	78	Community, univers	N/A	18-32	Men	N/A	N/A	Prostate	2 months	Testicular self-examin	Testicular self-examinati	Screening uptake	Events/total=	N/A	N/A	N/A	Cochrane Risk	High risks of sequence

			ity (Ireland)								ation demonstration video and once or twice implementation intention	on demonstration video only		43/53 vs. 21/25				of Bias tool	generation, allocation concealment and blinding of participants biases, otherwise low risks
Hewitson 2011	RCT	1,288	UK	N/A	N/A	N/A	N/A	N/A	Colorectal	20 weeks	1. GP-signed invitation letter to promote fecal occult blood test; 2. Enhanced procedural information leaflet to promote fecal occult blood test	A fecal occult blood test kit was only sent a week after first mailed letter	Screening uptake	Unadjusted OR (95% CIs)= 1.26 (1.01, 1.58)	Unadjusted OR (95% CIs)= 1.26 (1.01, 1.58)	N/A	N/A	Cochrane Risk of Bias tool	Overall low risk, with low risk of bias on all items
Hirst et al 2017	RCT	8,269	London (UK)	N/A	60-74	Men and	N/A	N/A	Colorectal	4 months	Automated text messag	Usual care: mailed fecal	Screening uptake	Events/total= 1674/	N/A	N/A	N/A	Cochrane Risk of	Overall low risk, with low risk of

						women					e reminder. Frequency of text message reminder: 1	occult blood test kit with printed reminders		4134 vs. 1648/4135				Bias tool	bias on all items apart from unclear for selective reporting
Hoare 1994	RCT	498	UK	N/A	N/A	Women	N/A	N/A	Breast	N/A	Trained link-workers contacted all women a few weeks before invitations were sent. If no information was obtained, a second visit was made. Link-workers conducted interviews in an	Received no visits	Screening uptake	Events/total= 122/247 vs. 117/251	N/A	N/A	N/A	Cochrane Risk of Bias tool + CASP criteria	High risk or concerns relating to all items, apart from baseline comparability and selective outcome reporting

											appropriate language, using a semi-structured questionnaire. A short explanation about breast screening was provided and women were encouraged to take up a forthcoming invitation								
Hol et al 2010	RCT	15,011	Netherlands	N/A	N/A	N/A	N/A	N/A	Colorectal	1 year	Use of fecal immunochemical test and patient mailed reminders	Usual care	Screening uptake	Unadjusted OR (95% CIs)= 1.63 (1.50, 1.77)	N/A	N/A	N/A	Self-modified	Low risks of selection, attrition and reporting biases, moderate for performance and detection bias

Hong 2014	RCT	923	Community (South Korea)	N/A	50-59	Men	N/A	N/A	Colorectal	3 months	1. Paper-based educational information sent by mail; 2. Educational information and promotion to undergo screening through telephone counselling	Usual care	Screening uptake	Events/total= 38/230 vs. 30/223	Events/total= 56/243 vs. 30/224	N/A	N/A	Cochrane Risk of Bias tool	Low risks of blinding of assessor, incomplete outcome data and selective outcome reporting biases, otherwise unclear risks
Hou et al 2005	RCT	424	Taiwan	Had not had a Pap test in the previous 12 months	30 years and older	Women	Chinese	N/A	Cervical	3 months	Three-month program utilising direct mail communication as well as a phone-counselling	Received a monthly newsletter with health information in general from the hospital	Screening uptake	Percentage = 50% of 212 vs. 32% of 212	N/A	N/A	N/A	Cochrane Risk of Bias tool	Low risks of blinding and selective reporting biases, high of selection and incomplete outcome data biases,

											component, received educational brochures with theory and evidence-based messages								unclear of other bias
Huf et al 2020	RCT	14,587	North West London (UK)	N/A	24-64	Women	N/A	N/A	Cervical	18 weeks	1. SMS without manipulation; 2. Primary care physician endorsed SMS	No SMS	Screening uptake	Adjusted OR (95% CIs)= 1.18 (1.02, 1.37)	Adjusted OR (95% CIs)= 1.19 (1.03, 1.38)	N/A	N/A	Cochrane Risk of Bias tool (version 2)	Overall low risk of bias, with low risks on all items
Hughes et al 2005	RCT (cluster)	3,358	GP records (rural Australia)	N/A	50-74	N/A	N/A	N/A	Colorectal	N/A	Test involves two samples collected with brush and no diet restrictions (fecal immunochemical test)	Test involves three samples collected with spatula and includes diet restrictions (guaiac-based faecal occult blood test	Screening uptake	Unadjusted OR (95% CIs)= 1.93 (1.61, 2.31)	N/A	N/A	N/A	Cochrane Risk of Bias tool	Overall high risk of bias, with high risks for selection, incomplete outcome data and other biases, while unclear for blinding and

PRISMA 2020 Checklist

											)							selective reporting
Hunt 1998	RCT 372	Community - identified from files at a women's clinic staffed by Aboriginal health workers in Danila Bilba (Australia)	Overdue	N/A	Women	Aboriginal	N/A	Cervical	3 months	1. Personal approach being approached by Aboriginal health workers and invited for screening; 2. Letter designed by Aboriginal workers stating individuals overdue for smear and inviting them to attend	Usual care with reminder tags for clinic staff attached to medical records	Screening uptake	Events/total= 4/60 vs. 0/61	Events/total= 2/63 vs. 0/61	N/A	N/A	Cochrane Risk of Bias tool	Low risk for random sequence generation, blinding and incomplete outcome data biases, unclear for allocation concealment, selective reporting and other biases

Ilic 2008	RCT	161	Community and online (Australia)	Had not previously been tested	>45 years	Men	N/A	N/A	Prostate	N/A	1. Mailed video containing standard educational information about prostate cancer; 2. Website containing standard educational information about prostate cancer	Mailed 28-pages pamphlet containing standard educational information about prostate cancer (content/messages identical as for intervention groups)	Screening uptake	Events/total= 32/53 vs. 34/49	Events/total= 42/54 vs. 34/49	N/A	N/A	Cochrane Risk of Bias tool	Low risks of bias on all items, apart from unclear for blinding of participants
Ingrand et al 2016	RCT (cluster)	304	France	Siblings of patients diagnosed with colorectal cancer or adenomatous polyps	N/A	N/A	N/A	N/A	Colorectal	12 months	Specialised screening nurse (familiar with applicability and constraints of	Usual care	Screening uptake	Events/total= 90/160 vs. 51/144	N/A	N/A	N/A	Cochrane Risk of Bias tool (version 2)	Overall low risk of bias, with low risks on all items

colonoscopy) performed counselling via telephone interviews and materials by mails to inform participants of the increased risk of colorectal cancer and benefits of colonoscopy. The information was tailored according to each participant’s psychosocial characteristics

Ishikawa et al 2012	RCT	3,236	Listed in local health department database (Japan)	No screening in past 2 years	51-59 (except 55 years)	Women	N/A	N/A	Breast	5 months	Individual assessment, assessment-based tailored letter to prompt study participants to participate in mammography screening (divided into three segments: high intention; low intention and high breast cancer worry; low intention and low breast cancer worry), particip	Non-tailored reminder	Screening uptake	OR (95% CIs)= 4.02 (2.67, 6.06)	N/A	N/A	N/A	Critical appraisal instruments from Joanna Briggs Institute for RCTs	Total score 9, with 'no' for similarity between groups at baselin and whether identical treatment of groups, unclear for concealed allocation and blinding of participants, otherwise 'yes' on all items

											ants then had to return postcard to receive tickets for free screening, which they could use at local clinics								
Jalili 2019	RCT	1,052	Canada	Unscreened women: no Pap test in registry and who had been registered for 5+ years. Non-responders: women who had been sent an invitation letter to be screened, but who remained unscreen	30-65	Women	N/A	N/A	Cervical	6 months	Direct mailing of self-sampling kit + reminder letter after 8 weeks for non-responders	No intervention beyond standard invitation	Screening uptake	Per protocol participation difference (fractions) between intervention & control (with 95% CIs)= 0.07 (0.04, 0.10)	N/A	N/A	N/A	Cochrane Risk of Bias tool	Low risks of bias on all items, apart from moderate for random sequence generation (not documented in study)

			ed															
Janda 2014	RCT	930	Community (Australia)	No previous history of melanoma	> 50 years	Men	N/A	N/A	Skin	7 months	Video-based skin awareness educational materials with a message from national sports personality and melanoma survivors + written educational materials	Written educational materials only	Screening uptake	Events/total= 246/436 vs. 229/434	N/A	N/A	N/A	Cochrane Risk of Bias tool	Low risks on all items
Janda et al 2011	RCT	929	Community (Australia)	N/A	>50 years	Men	N/A	N/A	Skin	N/A	Researcher guide and colour brochure containing	Researcher guide and colour brochure only	Screening uptake	Events/total= 153/420 vs. 126/411	N/A	N/A	N/A	Cochrane Risk of Bias tool	Overall rated as high methodological quality, with low risks of bias on

											educational information + body chart diagram + video/DVD + two postcard reminders								all items apart from unclear for allocation concealment and blinding of participants
Jensen et al 2009	RCT (cluster)	14,979	Local screening programme for cancer (Denmark)	Overdue	23-59	Women	N/A	N/A	Cervical	9 months	Personalised targeted invitation letter from GP. GP also received visit from facilitator to discuss ways to increase uptake of screening	Standard invitation letter only	Screening uptake	Events/total= 1448/7527 vs. 947/7452	N/A	N/A	N/A	Cochrane Risk of Bias tool	Unclear risks of bias on all items
Kellen 2018	RCT	35,895	Belgium	Without screening record since 8	30-64	Women	N/A	N/A	Cervical	1 year	1. Reminder mailing	1. Reminder mailing inviting	Screening uptake	Per protocol partic	Per protocol partici	N/A	N/A	Cochrane Risk of	Low risks of bias on all items, apart

				years							with self-sampling kit (mail-to-all); 2. Reminder mailing with self-sampling to be ordered (opt-in)	women to have a cytology specimen taken by a clinician (=routine intervention); 2. No invitation		ipation difference (fractions) between intervention & control (with 95% CIs)= no letter comparison= 0.11 (0.10, 0.12); recall letter comparison= 0.08 (0.07, 0.09)	pation difference (fractions) between intervention & control (with 95% CIs)= no letter comparison = 0.03 (0.02, 0.03); recall letter comparison = 0.00 (-0.01, 0.01)			Bias tool	from high for allocation concealment and moderate e for selective reporting
Kerrison et al 2015	RCT	2,240	UK	Due to be invited for first routine screen	47-53	Women	N/A	N/A	Breast	N/A	Text message reminder 48 hours before the appointment	Usual care	Screening uptake (proportion attending the appointment within 60 days	Events/total= 759/1122 vs. 703/1118	N/A	N/A	N/A	Cochrane Risk of Bias tool (version 2)	Overall low risk of bias, with low risks on all items

											and an additional text message if they did not attend the initial appointment		of the initial appointment)						
King et al 1994	RCT (cluster)	1,281	GP records (Australia)	N/A	45-75	N/A	N/A	N/A	Colorectal	N/A	Low literacy booklet with graphics and risk information sent with invitation pack	Standard invitation pack only	Screening uptake	Risk ratio (95% CIs)= 1.01 (0.92, 1.12)	N/A	N/A	N/A	Cochrane Risk of Bias tool	Overall unclear risk of bias, with unclear risk for selection, blinding of participants and personell and selective reporting biases, otherwise low

Kitchener 2017	RCT (cluster)	6,213	UK	Due for their first invitation and who in phase 1 of the 'STRATEGIC trial' did not respond to invitation letters (with or without pre-leaflet or with/without online booking) to screening after 6 months	20 (Grampian); 25 (Manchester)	Women	N/A	N/A	Cervical	18 months	1. Direct mailing of unrequested self-sampling kits (mail-to-all); 2. Direct mailing of requested self-sampling kits (opt-in)	No intervention beyond standard invitation	Screening uptake	**Per protocol participation difference (fractions) between intervention & control (with 95% CIs)= -0.09 (-0.11, -0.07)**	**Per protocol participation difference (fractions) between intervention & control (with 95% CIs)= -0.15 (-0.16, -0.13)**	N/A	N/A	Cochrane Risk of Bias tool	Moderate risks of selection biases, otherwise low
Kregting 2020	RCT	1,312	South West screening region of the Netherlands	N/A	60.1 (intervention mean); 59.9 (control mean)	Women	N/A	N/A	Breast	N/A	Official breast cancer screening information leaflet from the Dutch National Institute for Public Health	No intervention	Knowledge, informed choice	**Knowledge SMD (95% CIs)= 0.21 (0.09, 0.34) Informed choice Risk ratio (95% CIs)= 1.07**	N/A	N/A	N/A	Cochrane Risk of Bias tool (version 2)	Concerns related to the randomisation process and selection of the reported results

and the Environ ment. The leaflet was develo ped based on the opinion of experts and contain ed informa tion about: a) the screeni ng invitati on; b) the screeni ng process ; c) possibl e screeni ng outcom es; and d) benefit s and harms (overdi agnosis ,	**(0.99 , 1.15)**

											overtreatment, false-negatives and interval cancers)								
Lancaster 1992	RCT	2,131	General practices in North Manchester (UK)	Due	50-64	Women	N/A	N/A	Cervical	N/A	Cervical screening invitation sent with breast screening invitation	Breast screening invitation only sent	Screening uptake	Events/total= 151/908 vs. 89/886	N/A	N/A	N/A	Cochrane Risk of Bias tool	Low risk of incomplete outcome data, unclear risks on all other items
Levi et al 2011	RCT	12,537	Israel	N/A	N/A	N/A	N/A	N/A	Colorectal	N/A	Faecal immunological test	Guaiac-based faecal occult blood test	Screening uptake	Unadjusted OR (95% CIs)= 0.86 (0.80, 0.94)	N/A	N/A	N/A	Self-modified	Low reporting bias, otherwise moderate selection, performance, detection and attrition biases
Libby et al 2011	RCT	59,953	National screeni	N/A	50-74	N/A	N/A	N/A	Colorectal	N/A	1. "Know the	1. Advance notificatio	Screening uptake	Risk ratio (95%	Risk ratio (95%	N/A	N/A	Cochrane Risk	Overall low risk of bias,

			ng programme (Scotland)								facts” information booklet included with advance notification letter; 2. Advanced notification letter sent prior to standard invitation pack	n without booklet and followed by standard invitation pack; 2. Standard invitation pack only		CIs)= 0.99 (0.98, 1.01)	CIs)= 1.09 (1.08, 1.11)			of Bias tool	with low risks on all items apart from unclear for incomplete outcome data
Lilliecreutz 2020	RCT	6,606	Sweden	Did not respond to invitation for conventional screening and had not had a smear test in 6 years (ages 30-49) and 8 years (ages 50-64)	30-64	Women	N/A	N/A	Cervical	6 months	Direct mailing of self-sampling kit + annual invitation	No intervention beyond standard invitation	Screening uptake	Per protocol participation difference (fractions) between intervention & control (with 95%	N/A	N/A	N/A	Cochrane Risk of Bias tool	Low risks of bias on all items

														CIs)= 0.19 (0.17, 0.20)					
Lo et al 2014	RCT	23,180	National screening programme (UK)	N/A	60-69	N/A	N/A	N/A	Colorectal	N/A	Pre-formulated implementation intentions inserted into standard leaflet	Standard leaflet only	Screening uptake	Risk ratio (95% CIs)= 0.98 (0.95, 1.01)	N/A	N/A	N/A	Cochrane Risk of Bias tool	Overall unclear risk of bias, with unclear risks for allocation concealment, blinding of participants and personell and selective reporting biases, otherwise low
Lonnberg 2016	RCT	1,036	National screening programme (Norway)	Overdue (no smear within the past ~4 years)	25-69	Women	N/A	N/A	Cervical	6 months	Reminder letter with a scheduled appointment in 2-4 weeks time. There were limited possibilities for resched	Standard open reminder	Screening uptake	Events/total= 196/526 vs. 102/510	N/A	N/A	N/A	Cochrane Risk of Bias tool	Low risks of random sequence generation and incomplete outcome data biases, otherwise unclear risks on all items

											uling, and appointments were primarily during normal office hours. Women were not required to confirm their attendance in advance								
MacDonald 2021	RCT (cluster)	538	New Zealand	Had not had a smear test in ≥4 years	25-69	Women	N/A	N/A	Cervical	N/A	Direct offer of self-sampling kit when attending intervention clinics. Participants randomised to receive the intervention could	Offered a cervical smear when attending control clinics	Screening uptake	Per protocol participation difference (fractions) between intervention & control (with 95% CIs)=	N/A	N/A	N/A	Cochrane Risk of Bias tool	Moderate risks for random sequence generation and reporting of timelines biases (under Reporting), otherwise low risks of biases including for selective

											opt for a clinician taken HPV test or cervical smear			0.20 (0.16, 0.25)					reporting
MACS Group 2006	RCT	1,333	Australia	N/A	N/A	N/A	N/A	N/A	Colorectal	N/A	Use of choice between different screening tests (fecal immunochemical test, colonoscopy, flexible sigmoidoscopy plus fecal immunochemical test)	Usual care	Screening uptake	Unadjusted OR (95% CIs)= 1.65 (1.04, 2.64)	N/A	N/A	N/A	Self-modified	Low risks for selection, attrition and reporting biases, moderate for performance and detection bias
Mant 1992	RCT	1,588	UK	N/A	N/A	N/A	N/A	N/A	Colorectal	N/A	1. Advanced notification letter; 2. Postal mailing of fecal occult blood	Invitation for a health check	Screening uptake	Unadjusted OR (95% CIs)= 1.35 (0.99, 1.87)	Unadjusted OR (95% CIs)= 1.31 (0.98, 1.85)	N/A	N/A	Self-modified	Moderate risks for selection and performance and detection biases, high for attrition and reporting

PRISMA 2020 Checklist

										test kits									biases
Mathieu 2007	RCT	734	Australia	Considering a subsequent screening	70-71	Women	N/A	N/A	Breast	N/A	Booklet on options' outcomes, clinical problem, outcome probability, explicit values clarification, others' opinions, guidance with worksheet (Ottawa Decision Support Framework)	BreastScreen New South Wales brochure - includes information for women 70 + but no numeric information about the outcomes of screening	Multiple	Mean increase Knowledge = 2.62 (n=351) vs. 0.68 (n=357) (p< 0.001) Mean (SD) Decisional conflict= 20.1 (14.5) (n=315) vs. 21.9 (14.5) (n=295) Events/total Informed values-choice congruence =	N/A	N/A	N/A	Cochrane Risk of Bias tool	Low risks on all items, apart from unclear for blinding of participants and personnel

													227/309 vs. 136/279 Events/total Proportion undecided = 17/349 vs. 36/356 Mean Anxiety immediately after= 29.61 (n=321) vs. 29.34 (n=315)					
Mathieu 2010	RCT 412	Australia	Considering screening	N/A	Women	N/A	N/A	Breast	N/A	Internet programme + worksheet on options' outcomes, clinical problem,	Delayed intervention	Multiple	Mean Screening uptake= 52% (n=96) vs. 65% (n=127) (p= 0.05) Mean	N/A	N/A	N/A	Cochrane Risk of Bias tool	Low risks on all items, apart from unclear for allocation concealment, blinding of participa

outcome probabilities, explicit values clarification, others' opinions, guidance (worksheet with questions relevant to decision-making process; one or more questions that asked patients to clarify their preferences; summary)	(SD) Knowledge = 73.5 (27.6) (n=113) vs. 62.7 (27.6) (n=189) Mean Decisional conflict= 71% (n=91) vs. 64% (n=110) (p= 0.24) Events/total Informed values-choice congruence = 65/91 vs. 70/110 Events/tota	nts and personnel and selective reporting biases

														l Proportion undecided = 21/117 vs. 82/209 Mean Accurate risk perception = 3.02 (n=113) vs. 2.45 (n=189) (p< 0.001)					
McAvoy 1991	RCT	737	National screening programme in Leicester (England)	Overdue (not recorded as having had a smear test)	18-52	Women	Asian	N/A	Cervical	4 months	1. Posted multilingual printed material; 2. Face-to-face home visits with multilingual material	Received no intervention	Screening uptake	Events/total= 14/131 vs. 3/62	Risk ratio (95% CIs)= 5.83 (1.92, 17.67)	N/A	N/A	Cochrane Risk of Bias tool	Low risks of random sequence generation and incomplete outcome data biases, otherwise unclear risks on all items

McCaffery 2010	RCT	210	Australia	N/A	N/A	Women	N/A	N/A	Cervical	N/A	Written material	No information; repeat screening in six months	Knowledge (screening)	Mean (SD)= 81 (23.51) (n=77) vs. 72 (23.51) (n=71)	N/A	N/A	N/A	Cochrane Risk of Bias tool	Low risks on all items, apart from unclear for blinding of participants and personnel
McDowell 1989	RCT	2,034	Hospital-based family medical centre in Ottawa (Canada)	Due and overdue (no previous smear in past year)	18-35	Women	N/A	N/A	Cervical	1 year	1. GP letter and reminder letter after 21 days; 2. Telephone call	Usual care	Screening uptake	Events/total= 38/184 vs. 18/165	Events/total= 30/189 vs. 18/165	N/A	N/A	Cochrane Risk of Bias tool	Unclear risks of bias on all items, apart from low risk for incomplete outcome data
Moen et al 2020	RCT	10,360	Subdistricts of Bergen (Norway)	Non-exposed'	25-69	Women	Described as 'multi-ethnic'	N/A	Cervical	6 months	Multifaceted delivered in participants' languages	No intervention	Screening uptake	Risk ratio (95% CIs)= 1.12 (1.08, 1.16)	N/A	N/A	N/A	Effective Public Health Care Practice Project (EPHPP) quantitative study quali	Rated as moderate quality

																		ty assessment tool	
Morrell et al 2005	RCT	90,000	Community - New South Wales Pap Test Register (Australia)	Due (had not had a smear test for > 48 months)	20-69	Women	N/A	N/A	Cervical	90 days	Letter identical to that usually sent out to women at 27 months after latest Pap smear or letter giving a similar message, but phrased in a tone more sympathetic to other factors going on in the woman's life that might have stopped her	No intervention	Screening uptake	Events/total= 2630/59780 vs. 868/29919	N/A	N/A	N/A	Cochrane Risk of Bias tool	Unclear risks on all items, apart from low risk for incomplete outcome data

											from having the test to date								
Moss et al 2017	RCT (cluster)	11,67,017	National screening programme (UK)	N/A	60-74	N/A	N/A	N/A	Colorectal	N/A	Participants issued fecal immunochemical test	Participants issued guaiac-based fecal occult blood test	Screening uptake	Risk ratio (95% CIs)= 1.12 (1.11, 1.13)	N/A	N/A	N/A	Cochrane Risk of Bias tool	Overall low risk of bias, with low risks on all items apart from unclear for blinding of participants and personell
Mullins 2009	RCT	15,000	National screening programme (Australia)	Overdue	65-69	Women	N/A	N/A	Cervical	11 weeks	1. Invitation letter 2. Educational (printed material)	No intervention	Screening uptake	**Events/total= 235/5000 vs. 40/2500**	**Events/total= 215/4999 vs. 80/2500**	N/A	N/A	Cochrane Risk of Bias tool	Unclear risks on all items, apart from low risks for random sequence generation and incomplete outcome data biases
Nadarzynski et al 2012	RCT	606	UK	N/A	21 (mean)	Women	N/A	N/A	Cervical	N/A	Usual care + website delivery of HPV information, which	Usual care only: Basic description of cervical cancer	Risk perception (of cervical cancer and HPV infectio	SMD (95% CIs)= -0.04 (-0.22, 0.14)	N/A	N/A	N/A	Cochrane Risk of Bias tool (version 2)	Overall some concerns, with concerns related to randomis

											included the same basic information plus a description of the causal role of HPV in cervical cancer + risk factor, which included the basic information plus information about cervical cancer risk factors		n)						ation process and deviations from intended interventions, otherwise low risks of biases
Neter et al 2014	RCT	29,833	Medical insurance records (Israel)	N/A	50-74	N/A	N/A	N/A	Colorectal	6 months	Use of 'implementation intentions': instruction leaflet sent to particip	Standard invitation	Screening uptake	Unadjusted OR (95% CIs)= 1.18 (1.12, 1.24)	N/A	N/A	N/A	Cochrane Risk of Bias tool	Overall low risk of bias, with low risks on all items apart from unclear for blinding

PRISMA 2020 Checklist

											ants containing suggestions for overcoming common problems that individuals face in attempting to perform a fecal occult blood test, and an encouragement								of participants and personell
Ng et al 1998	RCT	1,25,525	Population registry (Singapore)	No screening in the past 1 year or biopsy within 6 months	50-64	Women	N/A	N/A	Breast	2 years	Letter invitation for a free screening; if no reply: 2 follow-up letters	No invitation sent	Stage (0 or 1) of cancer diagnosis	Percentage = 64% of 28231 vs. 26% of 97294	N/A	N/A	N/A	Critical appraisal instruments from Joanna Briggs Institute for RCTs	Total score 7, with 'yes' for blinding of participants, identical treatment of groups, complete follow-up, analysis

PRISMA 2020 Checklist

																			of participants in groups to which they were randomly assigned, same measurements for groups, reliable measures and appropriate trial design, otherwise unclear for all other items
Nichols 1986	RCT	17,824	UK	N/A	N/A	N/A	N/A	N/A	Colorectal	N/A	1. Printed information material (educational booklet) in addition to invitation letter; 2. Mailing fecal	1. Letter only; 2. Invitation to pick up kit at the clinic	Screening uptake	**Events/total= 2722/7170 vs. 2854/7227**	**Events/total= Scheduled appointment= 3108/8136 vs. 1809/3698 Open appointment= 3108/**	N/A	N/A	Cochrane Risk of Bias tool + CASP criteria	Concerns related to all items apart from selective outcome reporting

											occult blood test kit to residence				**8136 vs. 587/2142**				
O'Carroll et al 2015	RCT	39,762	National screening programme (Scotland)	N/A	50-74	N/A	N/A	N/A	Colorectal	N/A	1. Invitation pack included survey with questions to provoke anticipated regret; 2. Invitation pack included a survey including health locus of control questions	1. Invitation included survey without questions to provoke anticipated regret; 2. Standard invitation pack without survey	Screening uptake	**Risk ratio (95% CIs)= 1.01 (0.99, 1.03)**	**Risk ratio (95% CIs)= 1.00 (0.98, 1.01)**	N/A	N/A	Cochrane Risk of Bias tool	Overall low risk of bias, with low risks on all items apart from unclear for blinding of participants and personnel
O'Connor 1998	RCT	468	East London general practice (England)	N/A	N/A	Women	N/A	N/A	Breast	N/A	GP letter + explanatory leaflet + invitation from	Invitation from NHS breast screening programme only	Screening uptake	Events/total= 134/236 vs. 120/234	N/A	N/A	N/A	Cochrane Risk of Bias tool + CASP criter	Low risks of allocation, incomplete outcome data, baseline

											NHS breast screening programme							ia	comparability and selective outcome reporting biases, high for blinding of assessors and intention to intervene
Page et al 2006	RCT	3,144	Breast Screen New South Wales (Australia)	On electoral roll during March 2004 who had never attended Breast Screen New South Wales for a mammogramy in two Screening and Assessment Service catchment areas (Australia)	50-54	Women	98.6% White	All SES	Breast	12 weeks	1. Mail recall/reminder in addition to invitation letter; 2. Phone call reminder in addition to invitation letter	Invitation letter	Screening uptake	Events/total= 402/669 vs. 304/658	Events/total= 61/785 vs. 43/786	N/A	N/A	Cochrane Risk of Bias tool + CASP criteria	Low risks of biases related to sequence generation, selective outcome reporting, pre-selected population and intention-to-treat analaysis inclusion, high risks for concealment, blinding of assessor and incomplete

																		outcome data biases
Perestelo-Perez et al 2019	RCT	107	Spain	No previous screening	50-69	N/A	N/A	N/A	Colorectal	N/A	Web-based decision aid that was reviewed in the company of a researcher that included clinical information, probabilities of outcomes, explicit values clarification, guidance in decision-making and summary	No intervention	Informed choice	Informed choice Risk ratio (95% CIs)= 45.61 (6.35, 327.38)	N/A	N/A	N/A	Cochrane Risk of Bias tool	Unclear risk of bias due to blinding of participants and personnel (performance bias), selective reporting (reporting bias) and 'other bias'

											document including content explored and participant responses regarding their preferences								
Pérez-Lacasta 2019	RCT (cluster)	524	Spain	N/A	50.14 (intervention mean); 50.19 (control mean)	Women	N/A	N/A	Breast	N/A	Received a decision aid that was a leaflet with detailed information on the benefits and harms of screening. The decision aid provided information about options	Received a standard leaflet that did not mention harms and recommended accepting the invitation to participate in the biennial exams of the breast cancer screening programme (usual care)	Knowledge, informed choice, confidence, anxiety	Knowledge SMD (95% CIs)= 1.87 (1.47, 2.27) Informed choice Risk ratio (95% CIs)= 24.30 (3.35, 176.08) Confidence SMD (95% CIs)= -0.41	N/A	N/A	N/A	Cochrane Risk of Bias tool (version 2)	Overall high risk of bias, due to potential deviation from intended interventions, missing outcome data and the measurement of the outcome

											and associated benefits/harms and helped clarify congruence between decisions and personal values			(-0.74, -0.07) Anxiety SMD (95% CIs)= 0.09 (-0.25, 0.42)					
Piana 2011	RCT	9,334	Bouches-du-Rhône (France)	Did not respond to invitation for conventional screening and had not had a cervical smear in > 2 years	35-69	Women	N/A	N/A	Cervical	N/A	Direct mailing of self-sampling kit, preceded by a notification with an opt-out option	Invitation for conventional cytology	Screening uptake	Events/total= 939/4400 vs. 311/4934	N/A	N/A	N/A	Cochrane Risk of Bias tool	Low risks of random sequence generation and incomplete outcome data biases, moderate for allocation concealment and reporting of timelines, high for selective reporting
Pierce 1989	RCT	416	General practic	Due	N/A	Wom	N/A	N/A	Cervical	1 year	Letter asking women	No intervention	Screening uptake	Events/total=	N/A	N/A	N/A	Cochrane Risk	Low risk of incomple

		e (UK)			en					to have a smear			45/140 vs. 20/134				of Bias tool	te outcome data bias, otherwise unclear for all other items
Pignone 2013	RCT 911	Australia	N/A	50-70	Men	White and 'Non-white'	N/A	Prostate	N/A	Online rating and ranking task and discrete choice experiments (explicit value clarification)	Balance sheet task (implicit value clarification)	Intention to screen	Events/total= 458/609 vs. 233/302	N/A	N/A	N/A	Cochrane Risk of Bias tool	Unclear risks of sequence generation, allocation concealment and blinding of participants biases, otherwise low risks
Pritchard 1995	RCT 757	A university general practice in Perth (Australia)	Due	36-69	Women	N/A	Socioeconomically disadvantaged area	Cervical	1 year	1. Letter with open invitation to make appointment; 2. Letter with fixed appointment	Usual care	Screening uptake	Events/total= 27/103 vs. 16/93	Events/total= 26/84 vs. 16/93	N/A	N/A	Cochrane Risk of Bias tool	Low risks of random sequence generation and incomplete outcome data biases, otherwise unclear risks on all items

Qureshi 2021	RCT	10,810	Community venues near Oslo (Norway)	Non-exposed'	20-69	Women	Pakistani and Somali	N/A	Cervical	6 months	Educational session delivered in participants' languages	No intervention	Screening uptake	Risk ratio (95% CIs)= 1.11 (1.05, 1.17)	N/A	N/A	N/A	Effective Public Health Care Practice Project (EPHPP) quantitative study quality assessment tool	Rated as weak quality
Racey 2016	RCT	818	Rural areas - opportunistic screening within primary care in Ontario (Canada)	Overdue/ underscreened	30-70	Women	N/A	N/A	Cervical	N/A	1. Mailed HPV self-collected test 2. Invitation letter for Pap test	No intervention - opportunistic screening	Screening uptake	Risk ratio (95% CIs)= 3.7 (2.2, 6.4)	Events/total= 51/331 vs. 13/152	N/A	N/A	Cochrane Risk of Bias tool	Low risks of random sequence generation and incomplete outcome data biases, high risk of other bias, otherwise unclear risks on all items

Radde et al 2016	RCT	5,265	Population registry, Mainz communities (Germany)	N/A	30-65	Women	N/A	N/A	Cervical	3 years	1. Invitation letter for Pap test 2. Invitation letter for Pap test and information brochure	No invitation	Screening uptake	Events/total= 1911/2645 vs. 753/1241	Events/total= 1843/2630 vs. 753/1242	N/A	N/A	Cochrane Risk of Bias tool	Low risk of random sequence generation bias, high risk of incomplete outcome data bias, otherwise unclear risks on all items
Raine et al 2016	RCT	1,67,741	General practices (England)	Eligible for screening	60-74	N/A	N/A	Aimed to reduced socioeconomic gradient in uptake	Colorectal	N/A	Reminder letter with added banner and text	Standard letter	Screening uptake	Adjusted ORs (95% CIs)= 1.07 (1.03, 1.11)	N/A	N/A	N/A	Cochrane Risk of Bias tool	Overall low risk of bias, with low risks on all items
Rat et al 2014	RCT	173	France	At increased melanoma risk	43.2 (mean)	76% women	N/A	N/A	Skin	5 months	Primary care physician counselling using tailored feedback	No intervention	Skin examination (self-reported)	**Events/total= 51/97 vs. 28/76**	N/A	N/A	N/A	United States Preventive Services Task Force Procedure Manual	Rated as 'fair' quality (failed to meet at least 1 criterion but had no known issue that would invalidate its results)

Rat et al 2017	RCT (cluster)	951	National (France)	Previously unresponsive	60.9 (mean)	38.9% women	N/A	N/A	Colorectal	1 year	Provider alerts	No intervention	Screening uptake	Events/total= 123/496 vs. 94/455	N/A	N/A	N/A	Cochrane Risk of Bias tool	Overall low risk of bias, with low risks on all items apart from unclear for allocation concealment and blinding of participants
Reder 2017	RCT	1,206	Germany	N/A	Those aged 50 included	Women	N/A	N/A	Breast	N/A	Online decision aid that consisted of a static information part and an interactive part. In the information part, the chance of each outcome was expressed as	Received a booklet containing standard information, quality of the screening, breast cancer and its risk factors, procedure, interval cancers and symptoms, follow-up diagnoses, advantag	Knowledge, decisional conflict, informed choice	Knowledge SMD (95% CIs)= 0.30 (0.15, 0.44) Decisional conflict SMD (95% CIs)= -0.21 (-0.36, -0.06) Informed choice Risk ratio	N/A	N/A	N/A	Cochrane Risk of Bias tool (version 2)	High risk of bias, including for missing outcome data and measurement of the outcome

event rate per 200 women screened every 2 years for 20 years using absolute numbers accompanied by crowd figure. The advantages and disadvantages of the mammography screening programme and their probabilities were described. The interactive part	es and disadvantages of screening	(95% CIs)= 1.31 (1.04, 1.65)

											of the DA summarised the main points of the information part and encouraged engagement with the information								
Richardson 1994	RCT	890	Population-based screening programme (New Zealand)	N/A	50-64	Women	N/A	N/A	Breast	N/A	1. Invitation letter from GP; if no reply within 2 weeks a postal reminder was sent from the screening centre; 2. Teleph	1. Did not receive a letter with the invitation; 2. Postal reminder	Screening uptake	Events/total= 113/203 vs. 82/192	Events/total= 118/248 vs. 121/247	N/A	N/A	Cochrane Risk of Bias tool + CASP criteria	Low risks of biases related to baseline comparability, incomplete outcome data and selective outcome reporting, concerns related to allocation and blinding of

											one reminder (up to 3 calls made at different times of day)								assessors, high risk of bias related to intention to intervene
Ritvo et al 2015	RCT	5,240	Health centre (Canada)	50-74	N/A	N/A	N/A	N/A	Colorectal	1 year	1. Patient navigation (face-to-face and telephone). This included colorectal cancer screening education, nurses eliciting participants' preferences of screening type and providing appropriate	Usual care	Screening uptake	Events/total= 458/2629 vs. 214/2611	Events/total= 465/2629 vs. 319/2611	N/A	N/A	Cochrane Risk of Bias tool (version 2)	Overall some concerns, with low risks of bias on all items apart from some concerns related to effect of assignment to intervention

											access; 2. Faecal occult blood tests (instead of conventional colonoscopy)								
Roberto 2020	RCT	2,119	Regional organised programmes (Italy)	N/A	49.7 (mean)	Women	97.4% Italian (intervention); 96.2% Italian (control)	N/A	Breast	N/A	Online decision aid, non-static, with 19 screens, each covering 1 topic and answering a question. This included short coloured text, figures, bullet points and hyperlinks. The DA homepage used a	Standard brochure that combined the best information from participating centres' brochures. The brochure was an online static web page divided into 4 sections. This included black-and-white text and no figures. Absolute numbers were	Decisional conflict, informed choice	Decisional conflict Risk ratio (95% CIs)= 0.75 (0.56, 0.99) Informed choice Risk ratio (95% CIs)= 1.19 (1.02, 1.38)	N/A	N/A	N/A	Cochrane Risk of Bias tool (version 2)	High risk of bias, including for missing outcome data and measurement of the outcome

nudging-like approach to highlight the 4 main sections: What is breast cancer?; What is mammography screening?; What are its benefits and harms?; and What results can be expected from mammography screening? The aid allowed women to decide which section	reported about breast cancer

											s to access first, and move to other pages linked from the homepage. The aid provided a list of issues and concerns that possibly affect the screening decision. Each woman was asked to state the importance of each of these items								
Robinson et al 1994	RCT	153	One GP practice's records	N/A	50-74	N/A	N/A	N/A	Colorectal	N/A	1. Instructions do not	1. Instructions do include	Screening uptake	Risk ratio (95% CIs)=	Risk ratio (95% CIs)=	N/A	N/A	Cochrane Risk of	Overall unclear risk of bias, with

			(UK)								include dietary restrictions; 2. Testing completed over 6 days	dietary restrictions; 2. Testing completed over 3 days		1.42 (1.09, 1.83)	1.02 (0.80, 1.31)			Bias tool	unclear risks for random sequence generation, allocation concealment, blinding of participants and personnel and selective reporting, otherwise low
Robson 1989	RCT	1,605	Registered with a general practice in inner London (England)	Due	30-65	Women and men	N/A	N/A	Cervical	2 years	Patients had open access to a health promotion nurse and had their risk factors assessed and followed up by both their GP and the	Usual care (i.e. managed by GP alone)	Screening uptake	Events/total= 606/799 vs. 392/608	N/A	N/A	N/A	Cochrane Risk of Bias tool	Low risks of random sequence generation and incomplete outcome data biases, high risk of other bias, otherwise unclear risks on all items

										nurse								
Roshanai 2009	RCT 163	Cancer genetic clinic of Uppsala University Hospital (Sweden)	N/A	N/A	Women	N/A	N/A	Breast	N/A	Standard genetic counselling + nurse consultation	Standard genetic counselling alone	Risk accuracy	Events/total= ≤ 3 months= 28/73 vs. 24/74 >3 months= 18/68 vs. 17/71	N/A	N/A	N/A	Cochrane Risk of Bias tool	Low risks of biases related to blinding of participants, personnel and outcome assessors, unclear risks of random sequence generation, allocation concealment, incomplete outcome data, selective reporting and other biases
Ruparel et al 2019	RCT 229 (nested)	Primary care records and invited to a hospital setting for the	Smokers and former smokers (within 5 years of quitting)	60-75	N/A	N/A	N/A	Lung	N/A	5.5-minute information film and 10-page booklet. Particip	Booklet only	Screening uptake, knowledge, decisional conflict	Percentage Screening uptake= 76.7 % of 126 vs.	N/A	N/A	N/A	Mixed Methods Appraisal Tool	Assessed as 'yes' on all items, apart from 'no' to whether outcome assessors

PRISMA 2020 Checklist

Lung Cancer Screen Uptake Trial (subset of participants) (UK)	ants given 10 minutes to watch film and/or read booklet. Film and booklet discussed lung cancer, benefits and harms of screening, low dose computer tomography procedure, and possible results from the scan	78.9% of 120 Mean increase (SD) Objective knowledge = 2.16 (1.8) (n=120) vs. 1.84 (1.9) (n=109) Mean increase (SD) Subjective knowledge = 0.92 (1.0) (n=120) vs. 0.55 (1.0) (n=109) Mean (SD) Decisional confli	were blinded to the intervention

														ct= 8.5 (1.3) (n=120) vs. 8.2 (1.5) (n=109)					
Sancho-Garnier 2013	RCT	18,730	France	Had not responded to invitation for conventional screening and had not had a cervical smear in > 2 years	35-69	Women	N/A	N/A	Cervical	N/A	Direct mailing of self-sampling kit, preceded by a notification	Invitation for conventional cytology at an outpatient clinic. The invitation included a list of centres performing the test	Screening uptake	Per protocol participation difference (fractions) between intervention & control (with 95% CIs)= 0.16 (0.15, 0.17)	N/A	N/A	N/A	Cochrane Risk of Bias tool	Low risks of incomplete outcome data and reporting of timelines biases, otherwise unclear risks
Sandiford et al 2019	RCT	7,601	Nationwide (New Zealand)	N/A	N/A	N/A	Maoris, Pacific-Islanders	N/A	Colorectal	N/A	Active telephone follow-up and advice on logistical barriers	Reminder letter only	Screening uptake	Imputed gain (95% CIs)= Maori = 5.2% (1.8, 8.5)	N/A	N/A	N/A	Cochrane Risk of Bias tool (version 2)	Overall low risk of bias, with low risks on all items

											by ethnically aligned callers over 4 weeks, where possible linguistically aligned			Pacific-Islander= 3.6% (0.7, 6.4)					
Santare et al 2015	RCT	15,000	Population, screening and cancer registries (Latvia)	N/A	50-74	N/A	N/A	N/A	Colorectal	N/A	1. Instructions do not include dietary restrictions (Fecal immunochemical test); 2. Kit includes round test tube with two caps (OC Sensor); 3. Advanced notification	1. Instructions do include dietary restrictions (Guaiac-based faecal occult blood test); 2. Kit includes flat test tube with one cap (FOB Gold); 3. Standard invitation pack only	Screening uptake	Risk ratio (95% CIs)= 1.48 (1.41, 1.55)	Risk ratio (95% CIs)= 1.06 (1.02, 1.11)	Risk ratio (95% CIs)= 1.08 (1.04, 1.12)	N/A	Cochrane Risk of Bias tool	Overall unclear risk of bias, with unclear for blinding and selective reporting, otherwise low risks

											letter two weeks prior to receiving standard invitation pack								
Segnan 1998	RCT	16,454	Italy	N/A	N/A	Women	N/A	N/A	Breast, Cervical	N/A	1. Letter from GP; 2. Scheduled appointment	1. Letter 2. Open appointment	Screening uptake	**Events/total= Breast= 945/2013 vs. 837/2015 Cervical= 759/2100 vs. 647/2094**	**Events/total= Breast= 945/2013 vs. 683/2016 Cervical= 759/2100 vs. 474/2093**	N/A	N/A	Cochrane Risk of Bias tool + CASP criteria	High risk related to analysis of cluster RCTs, some concerns related to blinding of assessors, otherwise low risks
Segura 2001	RCT (cluster)	2,958	“Raval Nord” neighborhood of the city of Barcelona (Spain)	N/A	50-64	Women	N/A	Positive effect appeared restricted to women with lower educational levels	Breast	2 months	1. Letters sent by mail from the Primary Health Care Team; 2. Direct contact through a	Letters sent by mail from the program	Screening uptake	Events/total= 174/313 vs. 157/302	Events/total= 216/340 vs. 165/317	N/A	N/A	Cochrane Risk of Bias tool + CASP criteria	High risks of blinding of assessor, incomplete outcome data, intention-to-treat and cRTC analysis biases, otherwis

											trained professional								e low
Selva et al 2019	RCT	512	Catalonia (Spain)	N/A	50-69	Men and women	N/A	N/A	Colorectal	N/A	Non-tailored telephone education. Duration of calls: 5.25 min	Usual care: mailed fecal occult blood test kit with printed reminders	Screening uptake	Events/total= 122/256 vs. 102/256	N/A	N/A	N/A	Cochrane Risk of Bias tool	Low risks on all items, apart from high risk for blinding of participants and personnel bias
Senore et al 2015	RCT	20,701	National (Italy)	N/A	N/A	53.1% women	N/A	N/A	Colorectal	9 months	Pre-Fecal immunochemical test advance notification	No intervention	Screening uptake	Events/total= 3888/10257 vs. 3580/10444	N/A	N/A	N/A	Cochrane Risk of Bias tool	Overall high risk of bias, with unclear blinding and selective reporting biases, otherwise low
Seow 1998	RCT	1,428	Singapore	Had not responded	N/A	Women	N/A	N/A	Breast	N/A	1. Same letter as control but with a family information pack designed to address the most	Routine second reminder letter (with a screening date) sent through the mail	Screening uptake	Events/total= 38/500 vs. 35/500	Events/total= 57/428 vs. 35/500	N/A	N/A	Cochrane Risk of Bias tool + CASP criteria	High risk related to intention to intervene, otherwise low risks

											significant barriers to mammography; 2. Additional home visit to make contact with the woman and her family								
Shankleman et al 2014	RCT	9,113	General practices in East London (England)	Eligible for screening	59-70	N/A	Health promotion delivered by phone among ethnic diversity populations	Health promotion delivered by phone among low socioeconomic status populations	Colorectal	8 months	1. Health promotion over the telephone: received standard invitation plus bi-lingual advocates phoned subjects a week after	Control GP practices: received usual care (i.e. NHS bowel cancer screening programme (BCSP) invitation)	Screening uptake	Median uptake= 46.7% of 2034 vs. 39.1% of 5227	Median uptake= 43.8% of 1852 vs. 39.1% of 5228	N/A	N/A	Cochrane Risk of Bias tool	Overall high risk of bias, with high risk for incomplete outcome data bias, unclear risks for random sequence generation, allocation concealment, blinding of participants and personne

		sending letters (3 attempts in total) plus callers offered colorectal cancer information and answered participants' questions; 2. Face-to-face health promotion: received standard invitation letter plus an invitation to attend a group health information session		l and blinding of outcome assessment biases, low risk for selective reporting bias and inclusion of intention-to-treat analysis		

											at the GP practice, which were held monthly and attendees were offered additional pictorial and multi-lingual guides to the test procedure								
Sharp 1996	RCT	782	UK	N/A	N/A	N/A	N/A	N/A	Breast	N/A	1. Nurse delivered home interview with a patient-specific health education component	Letter from GP	Screening uptake	Events/total= 36/315 vs. 21/160	N/A	N/A	N/A	Cochrane Risk of Bias tool + CASP criteria	High risk related to intention to intervene, concerns related to baseline comparability, otherwise low risks

Smith et al 2010	RCT	530	Community-based (Australia)	Average risk of colorectal cancer or slightly above	55-64	N/A	N/A	Socioeconomically disadvantaged areas	Colorectal	N/A	Paper-based decision aid with DVD with and without question prompt list	Standard national information booklet	Multiple	Absolute difference (95% CIs) Screening uptake= -16 (-24, -8) Mean (SD) Knowledge = 54.17 (27.83) (n=357) vs. 34.17 (14.25) (n=173) Absolute difference (95% CIs) Attitudes= -14 (-23, -5) Absolute differ	N/A	N/A	N/A	Cochrane Risk of Bias tool	Low risks on all items

														ence (95% CIs) Informed choice= 22 (15, 29) Absolute difference (95% CIs) Decisional conflict= -13 (-22, -4)					
Stamatiou 2008	RCT	1,135	Clinic, university-based (Greece)	N/A	50-86	Men	N/A	N/A	Prostate	2 years	Regular recommendation by physician + additional educational leaflet pre-consultation	Regular recommendation by physician only	Screening uptake	Events/total= 442/548 vs. 227/587	N/A	N/A	N/A	Cochrane Risk of Bias tool	High risks for blinding of personnel and incomplete outcome data biases, low risk for selective outcome reporting, otherwise unclear

																			risks
Steadman 2004	RCT	76	Community, non-psychology undergraduates (UK)	N/A	18-35	Men	N/A	N/A	Prostate	3 weeks	Implementation intention requiring participant to decide when and where they would perform testicular self-examination in the next 3 weeks	Usual care	Screening uptake	Events/total= 30/46 vs. 12/30	N/A	N/A	N/A	Cochrane Risk of Bias tool	High risks for incomplete outcome data and other biases, unclear for allocation concealment, blinding of participants and blinding of assessor, otherwise low
Steckelberg 2011	RCT	1,577	Germany	No screening history	N/A	N/A	N/A	N/A	Colorectal	N/A	Brochure on options' outcomes, clinical problem, and outcome probabilities	Usual care using pamphlet	Multiple	Events/total Screening uptake= 141/785 vs. 134/792 Mean (SD) Knowledge	N/A	N/A	N/A	Cochrane Risk of Bias tool	Low risks on all items, apart from unclear risk of other bias

=
53.8
(28.8)
(n=78
5) vs.
31.3
(15)
(n=79
2)
Event
s/tota
l
Accur
ate
risk
perce
ption
s=
361/7
85 vs.
141/7
92
Event
s/tota
l
Infor
med
value
s-
choic
e
congr
uence
=
345/7
85 vs.
101/7
92

Stein et al 2005	RCT	1,140	Community (Devon, England)	Overdue (no record of screening in past 15 years)	39-64	Women	N/A	N/A	Cervical	3 months	1. Telephone call. Telephone call from experienced research nurse using a prepared script. Maxiumum of three attempts were made on consecutive days; 2. Letter from Health Authority District Cervical Screening Commisioner on behalf of National	No Intervention	Screening uptake	**Events/total= 4/111 vs. 1/95**	**Events/total= 13/219 vs. 1/95**	**Events/total= 5/221 vs. 1/95**	N/A	Cochrane Risk of Bias tool	Low risk for random sequence generation bias, high for incomplete outcome data, otherwise unclear

											Cervical Screening Programme; 3. Letter from a well known journalist and broadcaster (Claire Rayner) who was also Chair of the Patients Association								
Stephens et al 2007	RCT (cluster)	91	Adelaide (South Australia)	1st-degree relatives	> 18 years	57 % women	N/A	N/A	Colorectal	3 months	Information pamphlet sent to patient's relatives 1 week after operation regarding colorectal	Information provided to index patients by the treating surgeon regarding risk associated with family colorectal cancer	Screening uptake	Percentage = 6% of 32 vs. 8% of 59	N/A	N/A	N/A	Cochrane Risk of Bias tool (adaption)	Target sample size not achieved, otherwise low risks

											cancer risks, benefits to screening, cues and barriers								
Stoffel et al 2019	RCT	N/A	Spain	N/A	N/A	N/A	N/A	N/A	Colorectal	N/A	Invitation letters advocating social norms of undergoing screening, that a high proportion of individuals are already participating	Standard invitation letter	Screening uptake	Unadjusted OR (95% CIs)= 1.00 (0.85, 1.18)	N/A	N/A	N/A	Modified Jadad scale	Rated as moderate quality
Stoffel et al 2021	RCT	N/A	Cyprus	N/A	N/A	N/A	N/A	N/A	Colorectal	Eigth weeks	Invitation letters advocating social norms of undergoing screening, that a high	Standard invitation letter	Screening uptake	Unadjusted OR (95% CIs)= 1.01 (0.73, 1.41)	N/A	N/A	N/A	Modified Jadad scale	Rated as high quality

											proportion of individuals are already participating								
Sultana 2016	RCT	8,160	Victorian residents (Australia)	Never or underscreened (not screened in past 5 years)	30-69	Women	N/A	N/A	Cervical	6 months	Sent a pre-invitation letter to receive a self-sampling kit. The second letter was sent three weeks after the first, including an information brochure on HPV and cervical cancer, the collection device with	Received a single invitation letter (never-screened) or a standard reminder letter (under-screened) to have a Pap test. A Pap test brochure, form and pre-paid envelope similar to the intervention arm were included in the letter	Screening uptake	Events/total= 2270/7140 vs. 126/1020	N/A	N/A	N/A	Cochrane Risk of Bias tool	Low risks on all items, apart from unclear risk of other bias

											user instructions, an information form and a postage paid envelope for returning the sample and form								
Szarewski 2011	RCT	6,000	UK	Did not attend for conventional cervical screening	N/A	Women	N/A	N/A	Cervical	N/A	Mailing of a self-sampling device for HPV testing	Recall for Pap test at clinic in non-responders	Screening uptake	Events/total= 153/1500 vs. 68/1500	N/A	N/A	N/A	Cochrane Risk of Bias tool	Medium risks for selection biases, otherwise low
Thomas 2014	RCT	26	Community (Australia)	No history of prostate cancer	50-70	Men	N/A	N/A	Prostate	N/A	Received prostate-specific antigen fact sheet + attended 2-day community jury where three experts discussed the	Received prostate-specific antigen fact sheet only	Intention to screen	Events/total= 5/12 vs. 11/14	N/A	N/A	N/A	Cochrane Risk of Bias tool	High risk of blinding of participants bias, unclear for blinding of personnel, otherwise low risks

											benefits, harm and general information about prostate-specific antigen testing, with discussion between participants and experts								
Tinmouth et al 2015	RCT	3,594	Canada	N/A	N/A	N/A	N/A	N/A	Colorectal	6 months	Addition of a guaiac-based fecal occult blood test kit to a second mailed invitation	Standard invitation	Screening uptake	Unadjusted OR (95% CIs)= 2.35 (1.93, 2.90)	N/A	N/A	N/A	Self-modified	Low risks for attrition and reporting biases, moderate for selection and performance and detection biases
Tran 2015	RCT	1,170	From 86 general practice clinics (France	N/A	50-75	Men	N/A	N/A	Prostate	4 months	Visual decision aid	Usual care (physicians answered patients' questions	Screening intention	**Events/total= 331/586 vs. 432/578**	N/A	N/A	N/A	Cochrane Risk of Bias tool	Overall unclear risk of bias, with unclear risks for blinding

			)									)							and selective outcome reporting biases, otherwise low
Tranberg 2018	RCT	9,791	Screening programme (Denmark)	Did not reply to a first invitation and were due to a second reminder	30-64	Women	N/A	N/A	Cervical	6 months	1. Reminder mailing with self-sampling kit; 2. Reminder mailing with self-sampling to be ordered (opt-in). Women were also offered the possibility to contact a GP for collection of cytology specimen. For	Reminder mailing inviting women to have a cytology specimen taken by a clinician	Screening uptake	**Per protocol participation difference (fractions) between intervention & control (with 95% CIs)= -0.06 (-0.08, -0.04)**	**Per protocol participation difference (fractions) between intervention & control (with 95% CIs)= -0.17 (-0.19, -0.15)**	N/A	N/A	Cochrane Risk of Bias tool	Low risks of bias on all items

										both arms: reminder letter if response after 4 months								
Trevena et al 2008	RCT 314	Australia	Not previously screened	N/A	N/A	N/A	N/A	Colorectal	N/A	Age-gender-family history specific DA booklet with information on options, outcome probabilities, explicit values clarification, guidance (personal worksheet with steps in decision making) (Theory	Usual care by consumer guidelines recommending faecal occult blood testing	Multiple	Events/total Screening uptake= 117/134 vs. 124/137 Events/total Knowledge (positive score)= 28/134 vs. 8/137 Events/total Informed values-choice congr	N/A	N/A	N/A	Cochrane Risk of Bias tool	Unclear risks of blinding of participants and personnel and incomplete outcome data biases, otherwise low

											of planned behaviour)			uence = 14/134 vs. 2/137					
van Roon et al 2011	RCT	5,000	Municipal database (Netherlands)	N/A	50-74	N/A	N/A	N/A	Colorectal	8 months	Use of a mailed advanced notification letter two weeks prior to receiving standard invitation pack	Standard invitation pack only	Screening uptake	Unadjusted OR (95% CIs)= 1.20 (1.07, 1.34)	N/A	N/A	N/A	Cochrane Risk of Bias tool	Overall unclear risk of bias, with unclear for blinding of participants and personnel, incomplete outcome data and selective reporting, otherwise low
van Rossum et al 2008	RCT	20,623	Municipal database (Netherlands)	N/A	50-75	N/A	N/A	N/A	Colorectal	N/A	Sample from one bowel motion required, collected in tube (Fecal immunochemical test)	Sample from three bowel motions required collected on cards (Guaiac-based faecal occult blood test)	Screening uptake	Risk ratio (95% CIs)= 1.28 (1.24, 1.31)	N/A	N/A	N/A	Cochrane Risk of Bias tool	Overall low risk of bias, with unclear for blinding of outcome assessment, otherwise low

PRISMA 2020 Checklist

Verne et al 1993	RCT	1,842	GP practice records (UK)	N/A	40-75	N/A	N/A	N/A	Colorectal	N/A	1. Instructions do not include dietary restrictions; 2. Results of test are self-analysed at home; 3. Stool sample collected using a wipe	1. Instructions do include dietary restrictions; 2. Results posted to laboratory for analysis; 3. Standard stool sample collection from bowl	Screening uptake	Risk ratio (95% CIs)= 1.05 (0.96, 1.15)	Risk ratio (95% CIs)= 1.03 (0.92, 1.15)	Risk ratio (95% CIs)= 1.06 (0.95, 1.18)	N/A	Cochrane Risk of Bias tool	Overall unclear risk of bias, with unclear risks of selection, blinding of outcome assessment, selective reporting and other bias, otherwise low
Vidal et al 2014	RCT	12,786	Southern Barcelona metropolitan area (Spain)	N/A	N/A	N/A	N/A	N/A	Breast	5 months	Text message reminder three days before a scheduled appointment with or without a message, with a new appointment	No intervention	Screening uptake	Events/total= 2785/3719 vs. 5893/9067	N/A	N/A	N/A	Cochrane Risk of Bias tool (version 2)	Overall high risk of bias, with some concerns related to bias arising from the randomisation process and in selection of the reported result, otherwise low risks

											date if requested								
Vinker et al 2002	RCT	2,315	Two primary care clinics (Israel)	N/A	50-75	N/A	N/A	N/A	Colorectal	1 year	1. Physician reminder group; 2. Patient reminder: Phone call reminder; 3. Patient reminder: Letter reminder	Usual care	Screening uptake	Unadjusted OR (95% CIs)= 12.52 (4.58, 34.17)	Unadjusted OR (95% CIs)= 11.21 (3.98, 31.51)	Unadjusted OR (95% CIs)= 6.99 (2.44, 20.03)	N/A	Cochrane Risk of Bias tool	Overall high risk of bias, with high risks for incomplete outcome data and selective reporting, otherwise unclear risks
Virtanen 2011	RCT	4,160	Espoo municipalities (Finland)	Had not attended a screening after an invitation to screen (non-attenders)	30-60	Women	Immigrants had lowest participation rates in the program	N/A	Cervical	N/A	Received by mail a self-sampling kit, an information letter on the study, an informed consent document and a data	Received a new invitation letter with a new appointment for screening. They also received the same questionnaire as the self-sampling arm	Screening uptake	**Events/total= 337/1130 vs. 795/3030**	N/A	N/A	N/A	Cochrane Risk of Bias tool	Unclear risks of allocation concealment, incomplete outcome data and other biases, otherwise low risks

												sheet on HPV infections and cervical cancer screening								
Ward 1991	RCT	204 patients of 16 GPs	General practice in inner metropolitan region of Sydney (Australia)	Due	20-65	Women	N/A	N/A	Cervical	1 month	Maximal intervention: GP advised women of need for smear and offered to perform it immediately; GP attempted to persuade those not consenting during that consultation by exploring barriers	Minimal intervention: GP advised eligible women of need for smear and offered to perform it immediately. Those not consenting advised to make appointment for smear within a week	Screening uptake	Events/total= 60/89 vs. 52/95	N/A	N/A	N/A	Cochrane Risk of Bias tool	Unclear risks on all items, apart from low for incomplete outcome data	

											and reasons for self-exclusions. If still did not consent, GP advised making an appointment for smear within a week								
Wardle et al 2003	RCT	2,966	Trial centres to recruit patients registered with GPs in London (England)	A 'harder-to-reach' population eligible for screening	55-64	N/A	N/A	A 'harder-to-reach' population	Colorectal	3 months	Received a mailed psychoeducational intervention (booklet) three weeks before receiving the usual screening invitation. Booklet educational materials drew	Received usual care	Screening uptake	Percentage = 53.2% of 1453 vs. 49.9% of 1513	N/A	N/A	N/A	Cochrane Risk of Bias tool	Overall unclear risk of bias, with unclear risks for random sequence generation, allocation concealment, blinding of participants and personnel, blinding of outcome assessment and incomple

											on various frameworks; health belief model (HBM), Theory of Planned Behaviour and regret theories to address screening barriers and increase positive expectations								te outcome data biases, low risk for selective reporting bias and inclusion of intention-to-treat analysis
Wardle et al 2016	RCT (cluster)	2,65,434	Population database of GP patients (UK)	N/A	60-74	N/A	N/A	Aims to reduce the socioeconomic gradient of uptake	Colorectal	N/A	1. Letter from GP with invitation pack endorsing screening; 2. A narrative leaflet with	1. Standard invitation from screening organisation; 2. Standard invitation pack without narrative leaflet; 3.	Screening uptake	**Adjusted ORs (95% CIs)= 1.07 (1.04, 1.10)**	**Adjusted ORs (95% CIs)= 1.00 (0.96, 1.03)**	**Adjusted ORs (95% CIs)= 1.03 (0.99, 1.06)**	N/A	Cochrane Risk of Bias tool	Overall low risk of bias, with low risks on all items

presenting stories of previous participants sent with the invitation pack; 3. A 'gist' leaflet: Simplified version of the screening information leaflet designed for low literacy and numeracy readers (NB: Four interventions were reported on, but one	Standard invitation pack with standard information leaflet

											of these were also cited in Raine et al 2016 and is therefore excluded here)								
Watson 2006	RCT	997	UK	N/A	N/A	Men	N/A	N/A	Prostate	N/A	Leaflet on options' outcomes, clinical problem, outcome probability	Usual care	Screening uptake/ Knowledge	Events/total Screening uptake= 119/465 vs. 149/512 Mean (range) Knowledge = 75% (0-100%) (n=468) vs. 25% (0-100%) (n=52	N/A	N/A	N/A	Cochrane Risk of Bias tool	Low risks of random sequence generation, allocation concealment, blinding of outcome assessment and incomplete outcome data biases, otherwise unclear

														2)					
Watson et al 2013	RCT	11,579	National screening programme (UK)	N/A	60-74	N/A	N/A	N/A	Colorectal	N/A	Survey with medical and lifestyle questions sent within a few days of invitation pack	Standard information pack without survey	Screening uptake	Risk ratio (95% CIs)= 0.91 (0.88, 0.94)	N/A	N/A	N/A	Cochrane Risk of Bias tool	Overall low risk of bias, with unclear for allocation concealment and blinding of participants and personnel, otherwise low
Watts 2014	RCT	138	Australia	Had at least one first- or second-degree relative with a previous diagnosis of prostate cancer	40-79	Men	N/A	N/A	Prostate	1 year	Tailored online patient decision aid	Non-tailored online educational information	Screening uptake	Events/total= 29/42 vs. 36/48	N/A	N/A	N/A	Cochrane Risk of Bias tool	High risks of random sequence generation, incomplete outcome data and other biases, otherwise low
Wikström 2011	RCT	4,060	Sweden	Non-responder	39-60	Women	N/A	N/A	Cervical	N/A	Mailing of a self-sampling device for HPV	Recall for Pap test at clinic in non-responders	Screening uptake	Events/total= 779/2000 vs. 188/2	N/A	N/A	N/A	Cochrane Risk of Bias tool	Low risks of attrition and reporting biases, otherwis

										testing			060					e moderate
Williams 1989	RCT 392	UK	N/A	N/A	Women	N/A	N/A	Breast	N/A	Letter specified an appointment and women were asked to cancel or alter appointments, but not to confirm them	Open-ended letter inviting women to return a form indicating convenient times; appointment was then sent	Screening uptake	Events/total= 162/188 vs. 154/204	N/A	N/A	N/A	Cochrane Risk of Bias tool + CASP criteria	Some concerns related to allocation and selective outcome reporting biases (and incomplete outcome data), high risk of intention to intervene bias, otherwise low risks
Wilson 1987	RCT 250	Five general practices in the Nottingham Health Authority area (UK)	Due (recorded as never having a smear)	45-65	Women	N/A	N/A	Cervical	3 weeks	Sent an appointment + two reminders	Letter of invitation to make an appointment + two reminders	Screening uptake	Events/total= 56/118 vs. 39/122	N/A	N/A	N/A	Cochrane Risk of Bias tool	Low risks for allocation concealment and incomplete outcome data biases, otherwise unclear

Wilson et al 2015	RCT	3,408	National screening programme (Australia)	N/A	50-74	N/A	N/A	N/A	Colorectal	3 months	1. Tailored Personalised Decision Support group: received health information tailored to their stage of readiness to screen; 2. Non-Tailored Personalised Decision Support group: received an online booklet with information about colorectal cancer screening	Received usual care	Screening uptake	Unadjusted OR (95% CIs)= 0.94 (0.77, 1.15)	Unadjusted OR (95% CIs)= 0.96 (0.78, 1.17)	N/A	N/A	Cochrane Risk of Bias tool	Overall high risk of bias, with high risks for allocation concealment and blinding of participants and personnel biases, otherwise low

Wong 2021	RCT	402	Community centres in various districts (Hong Kong)	Non-exposed'	25 and above	Women	South Asian	N/A	Cervical	3 months	Multifaceted delivered in participants' languages	No intervention	Screening uptake	**Risk ratio (95% CIs)= 1.86 (1.63, 2.13)**	N/A	N/A	N/A	Effective Public Health Care Practice Project (EPHPP) quantitative study quality assessment tool	Rated as moderate quality
Youl 2005	RCT	1,322	Registered on the Queensland State Electoral Roll (Australia)	N/A	30-79	Men	N/A	N/A	Skin	N/A	Personalised motivational invitation letter signed by a well-known and popular Australian sportsman + factual mailed brochur	Only personalised motivational invitation letter signed by a well-known and popular Australian sportsman	Screening uptake	Events/total= 128/661 vs. 122/661	N/A	N/A	N/A	Cochrane Risk of Bias tool	Unclear risks for sequence generation, allocation concealment, blinding of participants and other biases, otherwise low risks on all items

											e containing educational information about melanoma and screening								
Youl 2015	RCT (parallel)	370	Community dwellers (participants from the Queensland electoral and Medicare rolls, Australia)	N/A	Intervention n= 31.6 (mean); control= 31.8 (mean)	67% women	N/A	N/A	Skin	12 months	Personalised text message reminders for improving sun protection habits based on the social cognitive theory, which used a conversational tone (weekly for the first three months and monthl	No intervention	Multiple	Unadjusted MD (95% CIs) Sun protection habits index = 3 months= 0.02 (-0.07, 0.11) 12 months= 0.13 (0.03, 0.23) Unadjusted risk ratio (95%	N/A	N/A	N/A	Cochrane Risk of Bias tool (version 2)	Overall high risk of bias, with high risk due to bias in measurement of the outcome and some concerns due to deviations from intended interventions, otherwise low risks

y during the following nine months)	CIs) Any skin self-examination in past 3 months= 3 months= 1.13 (0.84, 1.51) 12 months= 1.22 (0.95, 1.56) Unadjusted risk ratio (95% CIs) Whole-body skin self-examination at time of the last skin self-exami

														nation= 3 months= 1.02 (0.66, 1.57) 12 months= 1.27 (0.72, 2.25)					
Zajac et al 2010	RCT	1,800	GP records and electoral roll (Australia)	N/A	50+	N/A	N/A	N/A	Colorectal	3 subsequent rounds, but no information of their duration	Letter from GP with invitation pack endorsing screening	Standard invitation letter sent from central screening service	Screening uptake	RR (95% CIs)= Round 2= 1.25 (1.11, 1.41) Round 3= 1.18 (1.04, 1.35) Round 4= 1.33 (1.17, 1.51)	N/A	N/A	N/A	Cochrane Risk of Bias tool	Overall high risk of bias, but only high for other bias and unclear for blinding of outcome assessment, otherwise low risk on all items

Meta-analyses (other outcomes)

NB:

PLEASE NOTE THAT THE RESPECTIVE OUTCOMES WITHIN EACH INTERVENTION HAVE BEEN INDICATED IN BLUE SUBHEADINGS BELOW

COUNSELLING (AT THE CLINIC)

RISK ACCURACY

ANALYSIS	*K ESTIMATES*	*OR*	*95% CI*	*TAU^2*	*$Q_{W\,(DF)}$*	*$Q_{B\,(DF)}$*	*P-VALUE*
**AVERAGE ESTIMATE**	1	1.14	0.53, 2.46	<0.01	<0.01(0)		N/A

SELF-EXAMINATION (FOR SYMPTOMS)

ANALYSIS	*K ESTIMATES*	*OR*	*95% CI*	*TAU^2*	*$Q_{W\,(DF)}$*	*$Q_{B\,(DF)}$*	*P-VALUE*
**AVERAGE ESTIMATE**	1	1.90	1.03, 3.51	<0.01	<0.01(0)		N/A

COUNSELLING (IN THE COMMUNITY)

SCREENING INTENTION

ANALYSIS	*K ESTIMATES*	*OR*	*95% CI*	*TAU^2*	*$Q_{W\,(DF)}$*	*$Q_{B\,(DF)}$*	*P-VALUE*
**AVERAGE ESTIMATE**	1	0.19	0.04, 1.08	<0.01	<0.01(0)		N/A

DECISION AIDS

IMPROVED KNOWLEDGE

	K ESTIMATES	OR	95% CI (+PI)	TAU^2	$Q_{W\ (DF)}$	$Q_{B\ (DF)}$
	10	3.36	1.79, 6.31 (0.24, 344.55)	0.68	246.16(9)	
	7	3.88	1.52, 9.93	0.93	225.06(6)	
	1	1.99	1.11, 3.58	<0.01	<0.01(0)	
	4	4.09	0.47, 35.86	1.76	80.22(3)	
	2	5.82	2.33, 14.54	<0.01	0.58(1)	
						11.84(2)
	2	7.62	0.00, 2.2E+08	3.54	32.27(1)	
	3	2.15	0.55, 8.32	0.26	18.20(2)	
						0.83(1)
	1	29.72	14.39, 61.39	<0.01	<0.01(0)	
	1	1.99	1.11, 3.58	<0.01	<0.01(0)	
	2	5.82	2.33, 14.54	<0.01	0.58(1)	
	3	2.15	0.55, 8.32	0.26	18.20(2)	
						41.65(3)
**TEXT**						
	1	1.51	1.38, 1.66	<0.01	<0.01(0)	
	2	12.75	0.00, 3.6E+05	1.23	17.78(1)	
	4	2.70	1.25, 5.81	0.15	9.09(3)	
						12.23(2)
	0	N/A	N/A	N/A	N/A	
	0	N/A	N/A	N/A	N/A	
	6	2.81	1.52, 5.22	0.32	172.83(5)	
	1	29.72	14.39, 61.39	<0.01	<0.01(0)	
						33.78(1)
	1	1.72	1.31, 2.22	<0.01	<0.01(0)	
**AND ONLINE)**	2	3.03	0.02, 430.62	0.27	7.95(1)	
	1	2.03	1.33, 3.11	<0.01	<0.01(0)	
	1	4.43	3.16, 6.20	<0.01	<0.01(0)	
						7.95(1)
	1	2.03	1.34, 3.14	<0.01	<0.01(0)	
	0	N/A	N/A	N/A	N/A	
						N/A
	1	2.03	1.33, 3.11	<0.01	<0.01(0)	
	1	4.43	3.16, 6.20	<0.01	<0.01(0)	
						7.95(1)
**TEXT**						
XCL. EUROPE)	2	3.03	0.02, 430.62	0.27	7.95(1)	
	0	N/A	N/A	N/A	N/A	
						N/A
	0	N/A	N/A	N/A	N/A	

	1	4.43	3.19, 6.25	<0.01	<0.01(0)	
	0	N/A	N/A	N/A	N/A	
						N/A
	1	4.43	3.16, 6.20	<0.01	<0.01(0)	
	1	2.03	1.33, 3.11	<0.01	<0.01(0)	
						7.95(1)

INFORMED CHOICE

ANALYSIS	*K ESTIMATES*	*OR*	*95% CI*	*TAU²*	*$Q_{W (DF)}$*	*$Q_{B (DF)}$*	*P-VALUE*
**AVERAGE ESTIMATE**	9	3.47	1.47, 8.20	0.82	144.66(8)		<0.01
**PRINTED**	4	4.97	1.72, 14.35	0.21	11.28(3)		0.01
**AGE**							
50-59	1	24.30	3.35, 176.08	<0.01	<0.01(0)		N/A
60-69	2	5.42	2.44, 12.03	<0.01	0.25(1)		0.62
70+	1	2.91	2.06, 4.11	<0.01	<0.01(0)		N/A
						11.04(2)	<0.01*
**GENDER**							
WOMEN	2	6.66	0.00, 3.4E+06	1.73	4.28(1)		0.04
MEN	0	N/A	N/A	N/A	N/A		N/A
						N/A	N/A
**CANCER TYPE**							
BREAST	2	6.66	0.00, 3.4E+06	1.73	4.28(1)		0.04
COLORECTAL	2	5.42	2.44, 12.03	<0.01	0.25(1)		0.62
						0.04(1)	0.84
**GEOGRAPHICAL CONTEXT**							
REST OF EUROPE (EXCL. UK)	2	8.18	0.00, 45520.98	0.62	2.20(1)		0.14
REST OF THE WORLD	2	3.62	0.02, 681.66	0.19	1.60(1)		0.21
						1.05(1)	0.30
**ETHNICITY**	0	N/A	N/A	N/A	N/A		N/A
**AREA DEPRIVATION**	0	N/A	N/A	N/A	N/A		N/A
**RISK OF BIAS**							
UNCLEAR	3	4.28	1.46, 12.56	0.14	8.44(2)		0.01
HIGH	1	24.30	3.35, 176.08	<0.01	<0.01(0)		N/A
						2.75(1)	0.10
**ONLINE**	3	3.38	0.03, 452.48	3.24	13.35(2)		<0.01
**AGE**							
UNDER 50	1	1.19	1.02, 1.38	<0.01	<0.01(0)		N/A
50-59	1	1.31	1.04, 1.65	<0.01	<0.01(0)		N/A
						0.47(1)	0.49
**GENDER**							
WOMEN	2	1.22	0.70, 2.14	<0.01	0.47(1)		0.49
MEN	0	N/A	N/A	N/A	N/A		N/A
						N/A	N/A
**CANCER TYPE**							
BREAST	2	1.22	0.70, 2.14	<0.01	0.47(1)		0.49
COLORECTAL	1	45.61	6.35, 327.38	<0.01	<0.01(0)		N/A
						12.88(1)	<0.01*
**GEOGRAPHICAL CONTEXT**							
REST OF EUROPE	3	3.38	0.03, 452.48	3.24	13.35(2)		<0.01
OTHER	0	N/A	N/A	N/A	N/A		N/A
						N/A	N/A
**ETHNICITY**	0	N/A	N/A	N/A	N/A		N/A
**AREA DEPRIVATION**	0	N/A	N/A	N/A	N/A		N/A
**RISK OF BIAS**							
UNCLEAR	1	45.61	6.35, 327.38	<0.01	<0.01(0)		N/A
HIGH	2	1.22	0.70, 2.14	<0.01	0.47(1)		0.49
						12.88(1)	<0.01*
**COMBINED (PRINTED AND ONLINE)**	2	2.33	0.01, 994.47	0.37	5.72(1)		0.02
**AGE**							
UNDER 50	1	1.43	0.79, 2.59	<0.01	<0.01(0)		N/A

50-59	1	3.71	2.24, 6.15	<0.01	<0.01(0)		N/A
						5.72(1)	0.02*
**GENDER**							
WOMEN	1	1.43	0.79, 2.60	<0.01	<0.01(0)		N/A
MEN	0	N/A	N/A	N/A	N/A		N/A
						N/A	N/A
**CANCER TYPE**							
BREAST	1	1.43	0.79, 2.59	<0.01	<0.01(0)		N/A
COLORECTAL	1	3.71	2.24, 6.15	<0.01	<0.01(0)		N/A
						5.72(1)	0.02*
**GEOGRAPHICAL CONTEXT**							
REST OF THE WORLD (EXCL. EUROPE)	2	2.33	0.01, 994.47	0.37	5.72(1)		0.02
OTHER	0	N/A	N/A	N/A	N/A		N/A
						N/A	N/A
**ETHNICITY**	0	N/A	N/A	N/A	N/A		N/A
**AREA DEPRIVATION**							
MOST DEPRIVED	1	3.71	2.24, 6.16	<0.01	<0.01(0)		N/A
LEAST DEPRIVED	0	N/A	N/A	N/A	N/A		N/A
						N/A	N/A
**RISK OF BIAS**							
LOW	1	3.71	2.24, 6.15	<0.01	<0.01(0)		N/A
UNCLEAR	1	1.43	0.79, 2.59	<0.01	<0.01(0)		N/A
						5.72(1)	0.02*

	K ESTIMATES	*OR*	*95% CI*	*TAU²*	$Q_{W (DF)}$	$Q_{B (DF)}$	*P-VALUE*
	8	0.81	0.64, 1.02	0.02	14.55(7)		0.04
	3	0.93	0.56, 1.55	0.01	2.28(2)		0.32
	2	1.10	0.39, 3.12	<0.01	0.24(1)		0.62
	1	0.80	0.60, 1.06	<0.01	<0.01(0)		N/A
						2.04(1)	0.15
	1	0.80	0.60, 1.06	<0.01	<0.01(0)		N/A
	2	1.10	0.39, 3.12	<0.01	0.24(1)		0.62
						2.04(1)	0.15
	1	0.80	0.60, 1.06	<0.01	<0.01(0)		N/A
	2	1.10	0.39, 3.12	<0.01	0.24(1)		0.62
						2.04(1)	0.15
**EXT**							
XCL. EUROPE)	3	0.93	0.56, 1.55	0.01	2.28(2)		0.32
	0	N/A	N/A	N/A	N/A		N/A
						N/A	N/A
	0	N/A	N/A	N/A	N/A		N/A
	0	N/A	N/A	N/A	N/A		N/A
	3	0.93	0.56, 1.55	0.01	2.28(2)		0.32
	0	N/A	N/A	N/A	N/A		N/A
						N/A	N/A
	4	0.72	0.51, 1.01	0.01	5.93(3)		0.11
	1	0.75	0.56, 0.99	<0.01	<0.01(0)		N/A
	1	0.68	0.52, 0.90	<0.01	<0.01(0)		N/A
	2	0.62	0.01, 35.91	0.17	5.32(1)		0.02

PRISMA 2020 Checklist

DECISIONAL CONFLICT

RISK ACCURACY

	K	OR	95% CI	TAU²	$Q_{W (DF)}$	$Q_{B (DF)}$	P-VALUE
						0.39(2)	0.82
	2	0.71	0.38, 1.33	<0.01	0.24(1)		0.63
	1	0.43	0.25, 0.71	<0.01	<0.01(0)		N/A
						3.15(1)	0.08
	2	0.71	0.38, 1.33	<0.01	0.24(1)		0.63
	1	0.82	0.69, 0.97	<0.01	<0.01(0)		N/A
	1	0.43	0.26, 0.72	<0.01	<0.01(0)		N/A
						5.70(2)	0.06
**EXT**	1	0.43	0.26, 0.72	<0.01	<0.01(0)		N/A
	3	0.77	0.61, 0.97	<0.01	1.36(2)		0.51
						4.58(1)	0.03*
	0	N/A	N/A	N/A	N/A		N/A
	0	N/A	N/A	N/A	N/A		N/A
	2	0.62	0.01, 35.91	0.17	5.32(1)		0.02
	2	0.71	0.38, 1.33	<0.01	0.24(1)		0.63
						0.16(1)	0.69
**ND ONLINE)**	1	1.43	0.79, 2.60	<0.01	<0.01(0)		N/A

ANALYSIS	*K ESTIMATES*	*OR*	*95% CI*	*TAU²*	*$Q_{W (DF)}$*	*$Q_{B (DF)}$*	*P-VALUE*
**AVERAGE ESTIMATE (PRINTED)**	2	5.95	0.01, 2497.68	0.38	6.02(1)		0.01
**AGE**							
50-59	1	10.26	4.94, 21.31	<0.01	<0.01(0)		N/A
60-69	1	3.93	3.12, 4.95	<0.01	<0.01(0)		N/A
						6.02(1)	0.01*
**GENDER**							
WOMEN	0	N/A	N/A	N/A	N/A		N/A
MEN	1	10.26	4.94, 21.31	<0.01	<0.01(0)		N/A
						N/A	N/A
**CANCER TYPE**							
COLORECTAL	1	3.93	3.12, 4.95	<0.01	<0.01(0)		N/A
PROSTATE	1	10.26	4.94, 21.31	<0.01	<0.01(0)		N/A
						6.02(1)	0.01*
**GEOGRAPHICAL CONTEXT**							
REST OF EUROPE (EXCL. UK)	1	3.93	3.12, 4.95	<0.01	<0.01(0)		N/A
REST OF THE WORLD	1	10.26	4.94, 21.31	<0.01	<0.01(0)		N/A
						6.02(1)	0.01*

PROPORTION UNDECIDED

	K ESTIMATES	*OR*	*95% CI*	*TAU²*	*$Q_{W (DF)}$*	*$Q_{B (DF)}$*	*P-VALUE*	
	2	0.39	0.06, 2.63	<0.01	0.54(1)		0.46	
	1	0.46	0.25, 0.83	<0.01	<0.01(0)		N/A	N/A
**ND ONLINE)**	1	0.34	0.20, 0.59	<0.01	<0.01(0)		N/A	N/A

	K	OR	95% CI	TAU²	Qw (DF)	QB (DF)	P-VALUE
**ETHNICITY**	0	N/A	N/A	N/A	N/A		N/A
**AREA DEPRIVATION**	0	N/A	N/A	N/A	N/A		N/A
**RISK OF BIAS**							
UNCLEAR	2	5.95	0.01, 2497.68	0.38	6.02(1)		0.01
OTHER	0	N/A	N/A	N/A	N/A		N/A
						N/A	N/A

TIVE TOWARDS

SCREE

DENCE

SCREENING INTENTION

ETY

ANALYSIS	*K ESTIMATES*	*OR*	*95% CI*	*TAU²*	*$Q_{W (DF)}$*	*$Q_{B (DF)}$*	*P-VALUE*
**AVERAGE ESTIMATE (PRINTED FORM)**	1	0.44	0.34, 0.56	<0.01	<0.01(0)		N/A

TIONAL

INFORI

NOWLEDGE

ANALYSIS	*K ESTIMATES*	*OR*	*95% CI*	*TAU²*	*$Q_{W (DF)}$*	*$Q_{B (DF)}$*	*P-VALUE*
**AVERAGE ESTIMATE COMBINED (PRINTED AND ONLINE)**	1	0.57	0.39, 0.82	<0.01	<0.01(0)		N/A
**AREA DEPRIVATION**							
MOST DEPRIVED	1	0.57	0.39, 0.82	<0.01	<0.01(0)		N/A
LEAST DEPRIVED	0	N/A	N/A	N/A	N/A		N/A
						N/A	N/A

ANALYSIS	*K ESTIMATES*	*OR*	*95% CI*	*TAU²*	*$Q_{W (DF)}$*	*$Q_{B (DF)}$*	*P-VALUE*
**AVERAGE ESTIMATE (PRINTED FORM)**	1	0.48	0.26, 0.88	<0.01	<0.01(0)		N/A

ANALYSIS	*K ESTIMATES*	*OR*	*95% CI*	*TAU²*	*$Q_{W (DF)}$*	*$Q_{B (DF)}$*	*P-VALUE*
**AVERAGE ESTIMATE (PRINTED FORM)**	1	1.18	0.64, 2.14	<0.01	<0.01(0)		N/A

	K ESTIMATES	*OR*	*95% CI*	*TAU²*	*$Q_{W (DF)}$*	*$Q_{B (DF)}$*
	5	2.08	0.92, 4.71	0.36	28.13(4)	
	2	1.41	0.65, 3.04	<0.01	0.37(1)	
	1	1.27	0.85, 1.85	<0.01	<0.01(0)	
	1	1.46	1.18, 1.85	<0.01	<0.01(0)	
						0.37(1)
	2	1.41	0.65, 3.04	<0.01	0.37(1)	
	0	N/A	N/A	N/A	N/A	
						N/A
	2	1.41	0.65, 3.04	<0.01	0.37(1)	
	0	N/A	N/A	N/A	N/A	
						N/A
**XT**						
	2	1.41	0.65, 3.04	<0.01	0.37(1)	
	0	N/A	N/A	N/A	N/A	
						N/A
	0	N/A	N/A	N/A	N/A	

	0	N/A	N/A	N/A	N/A	
	1	1.27	0.85, 1.85	<0.01	<0.01(0)	
	1	1.46	1.18, 1.85	<0.01	<0.01(0)	
						0.37(1)
	1	6.96	3.90, 12.44	<0.01	<0.01(0)	
E	1	2.07	1.52, 2.76	<0.01	<0.01(0)	
D VIDEO)	1	1.67	1.03, 2.69	<0.01	<0.01(0)	
	1	1.67	1.03, 2.69	<0.01	<0.01(0)	
	0	N/A	N/A	N/A	N/A	
						N/A

INFORMED CHOICE

RISK PERCEPTION

ANALYSIS	*K ESTIMATES*	*OR*	*95% CI*	*TAU*^{2}	$Q_{W\ (DF)}$	$Q_{B\ (DF)}$	*P-VALUE*
**AVERAGE ESTIMATE (PRINTED FORMAT)**	1	1.07	0.99, 1.15	<0.01	<0.01(0)		N/A

ANALYSIS *K ESTIMATES* *OR* *95% CI* *TAU*^{2} $Q_{W\ (DF)}$ $Q_{B\ (DF)}$ *P-VALUE*

ANALYSIS	*K ESTIMATES*	*OR*	*95% CI*	*TAU*^{2}	$Q_{W\ (DF)}$	$Q_{B\ (DF)}$	*P-VALUE*
**AVERAGE ESTIMATE (ONLINE FORMAT)**	2	0.91	0.54, 1.52	<0.01	0.08(1)		0.77
**AGE**							
UNDER 50	2	0.91	0.54, 1.52	<0.01	0.08(1)		0.77
50+	0	N/A	N/A	N/A	N/A		N/A
						N/A	N/A
**GENDER**							
WOMEN	2	0.91	0.54, 1.52	<0.01	0.08(1)		0.77
MEN	0	N/A	N/A	N/A	N/A		N/A
						N/A	N/A
**CANCER TYPE**							
BREAST	1	[illegible]	[illegible]	<0.01	<0.01(0)		N/A
CERVICAL	1	[illegible]	[illegible]	<0.01	<0.01(0)		N/A
						0.08(1)	0.77
MOST DEPRIVED	1	1.46	0.91, 2.35	<0.01	<0.01(0)		N/A
LEAST DEPRIVED	0	N/A	N/A	N/A	N/A		N/A
						N/A	N/A

DECISIONAL CONFLICT

ANALYSIS	*K ESTIMATES*	*OR*	*95% CI*	*TAU*^{2}	$Q_{W\ (DF)}$	$Q_{B\ (DF)}$	*P-VALUE*
**AVERAGE ESTIMATE**	2	1.68	0.46, 6.13	<0.01	0.39(1)		0.53
**PRINTED**	1	[illegible]	[illegible]	<0.01	<0.01(0)		N/A
**COMBINED (PRINTED AND ONLINE)**	1	[illegible]	[illegible]	<0.01	<0.01(0)		N/A
**AREA DEPRIVATION**							

LETTER

**GEOGRAPHICAL CONTEXT**							
UK	1	0.93	0.67, 1.29	<0.01	<0.01(0)		N/A
REST OF EUROPE	1	0.85	0.51, 1.41	<0.01	<0.01(0)		N/A
						0.08(1)	0.77
**ETHNICITY**	0	N/A	N/A	N/A	N/A		N/A
**AREA DEPRIVATION**	0	N/A	N/A	N/A	N/A		N/A
**RISK OF BIAS**							
UNCLEAR	1	0.93	0.67, 1.29	<0.01	<0.01(0)		N/A
HIGH	1	0.85	0.51, 1.41	<0.01	<0.01(0)		N/A
						0.08(1)	0.77

(INVITATION ONLY)

PERCENTAGE STAGE 0 OR 1 CANCER DIAGNOSIS

PATIENT

ANALYSIS	*K ESTIMATES*	*OR*	*95% CI*	*TAU*^{2}	$Q_{W\,(DF)}$	$Q_{B\,(DF)}$	*P-VALUE*
**AVERAGE ESTIMATE**	1	5.06	4.92, 5.20	<0.01	<0.01(0)		N/A

NAVIGATION

TIME TO FIRST CONSULTATION

ANALYSIS	*K ESTIMATES*	*OR*	*95% CI*	*TAU*^{2}	$Q_{W\,(DF)}$	$Q_{B\,(DF)}$	*P-VALUE*
**AVERAGE ESTIMATE**	1	1.22	0.90, 1.65	<0.01	<0.01(0)		N/A

FOLLOW-UP CONSULTATIONS

ANALYSIS	*K ESTIMATES*	*OR*	*95% CI*	*TAU*^{2}	$Q_{W\,(DF)}$	$Q_{B\,(DF)}$	*P-VALUE*
**AVERAGE ESTIMATE**	1	1.40	1.08, 1.82	<0.01	<0.01(0)		N/A

PHONE FOLLOW-UP (TEXT MESSAGES ONLY)

IMPROVED KNOWLEDGE

ANALYSIS	*K ESTIMATES*	*OR*	*95% CI*	*TAU*^{2}	$Q_{W\,(DF)}$	$Q_{B\,(DF)}$	*P-VALUE*
**AVERAGE ESTIMATE**	1	1.01	0.84, 1.20	<0.01	<0.01(0)		N/A

SELF-EXAMINATION (FOR SYMPTOMS)

PRISMA 2020 Checklist

ANALYSIS	*K ESTIMATES*	*OR*	*95% CI*	*TAU*^{2}	$Q_{W\,(DF)}$	$Q_{B\,(DF)}$	*P-VALUE*
**AVERAGE ESTIMATE**	1	1.27	0.72, 2.25	<0.01	<0.01(0)		N/A

Table abbreviations: OR = odds ratio; CI = confidence intervals; (Cochran's) Q_W = measure of within-group heterogeneity; (Cochran's) Q_B = measure of between-group heterogeneity (with between-group effects of p-value <0.05 indicated with *); DF = degrees of freedom; PI = prediction intervals.

RISK ASSESSMENT

RISK PERCEPTION

ANALYSIS	*K ESTIMATES*	*OR*	*95% CI*	*TAU*^{2}	$Q_{W\,(DF)}$	$Q_{B\,(DF)}$	*P-VALUE*
**AVERAGE ESTIMATE**	3	1.19	0.26, 5.37	0.23	5.73(2)		0.06
**AGE**							
UNDER 50	3	1.19	0.26, 5.37	0.23	5.73(2)		0.06
50+	0	N/A	N/A	N/A	N/A		N/A
						N/A	N/A
**GENDER**							
WOMEN	3	1.19	0.26, 5.37	0.23	5.73(2)		0.06
MEN	0	N/A	N/A	N/A	N/A		N/A
						N/A	N/A
**CANCER TYPE**							
BREAST	3	1.19	0.26, 5.37	0.23	5.73(2)		0.06
OTHER	0	N/A	N/A	N/A	N/A		N/A
						N/A	N/A
**GEOGRAPHICAL CONTEXT**							
UK	3	1.19	0.26, 5.37	0.23	5.73(2)		0.06
OTHER	0	N/A	N/A	N/A	N/A		N/A
						N/A	N/A
**ETHNICITY**	0	N/A	N/A	N/A	N/A		N/A
**AREA DEPRIVATION**	0	N/A	N/A	N/A	N/A		N/A
**RISK OF BIAS**							
UNCLEAR	2	1.42	0.00, 1495.81	0.50	5.64(1)		0.02
HIGH	1	0.87	0.36, 2.09	<0.01	<0.01(0)		N/A
						0.48(1)	0.49

Funnel plots

Outcome: Screening uptake

Choice of methods

SURVEY

SCREENING INTENTION

ANALYSIS	*K ESTIMATES*	*OR*	*95% CI*	*TAU*^{2}	$Q_{W\,(DF)}$	$Q_{B\,(DF)}$	*P-VALUE*
**AVERAGE ESTIMATE**	1	0.90	0.65, 1.24	<0.01	<0.01(0)		N/A

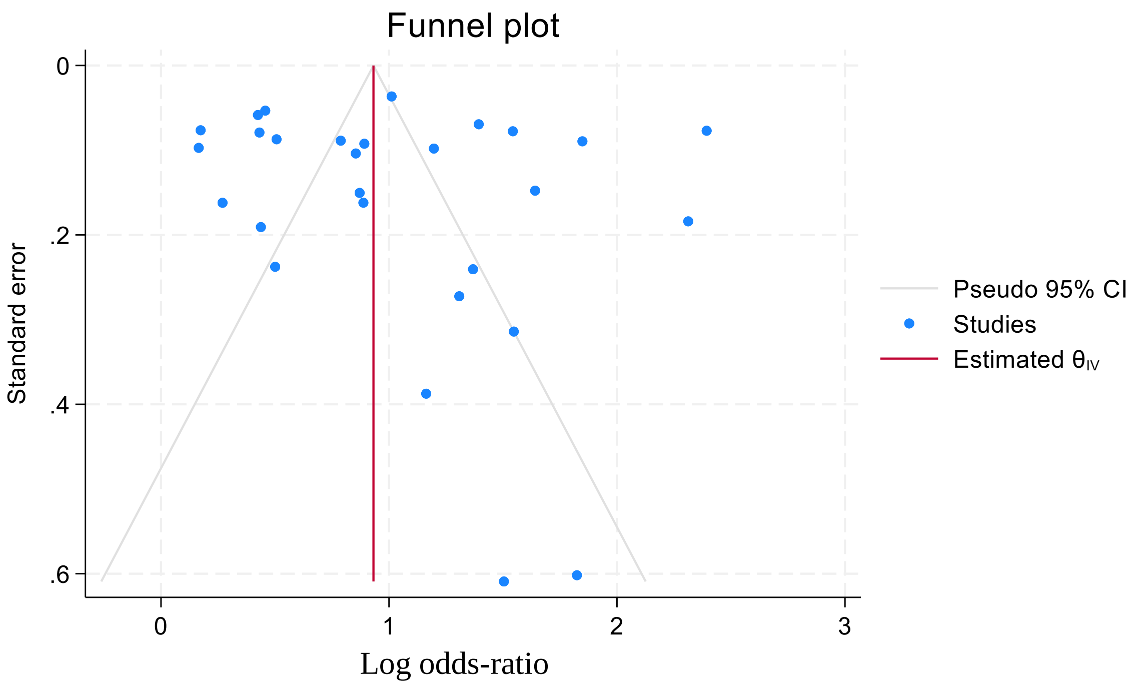
Funnel plot
Standard error
0
.2
.4
.6
0
1
2
3
Log odds-ratio
Pseudo 95% CI
Studies
Estimated θ_{IV}

Decision aids (overall)

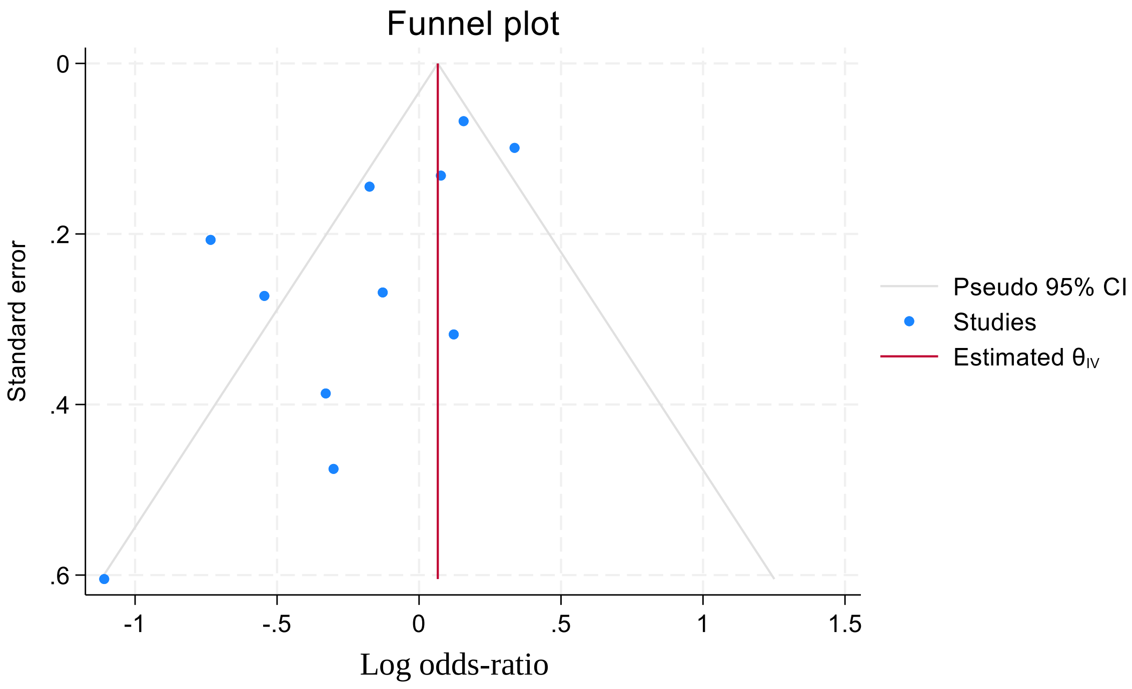

Educational information (overall)

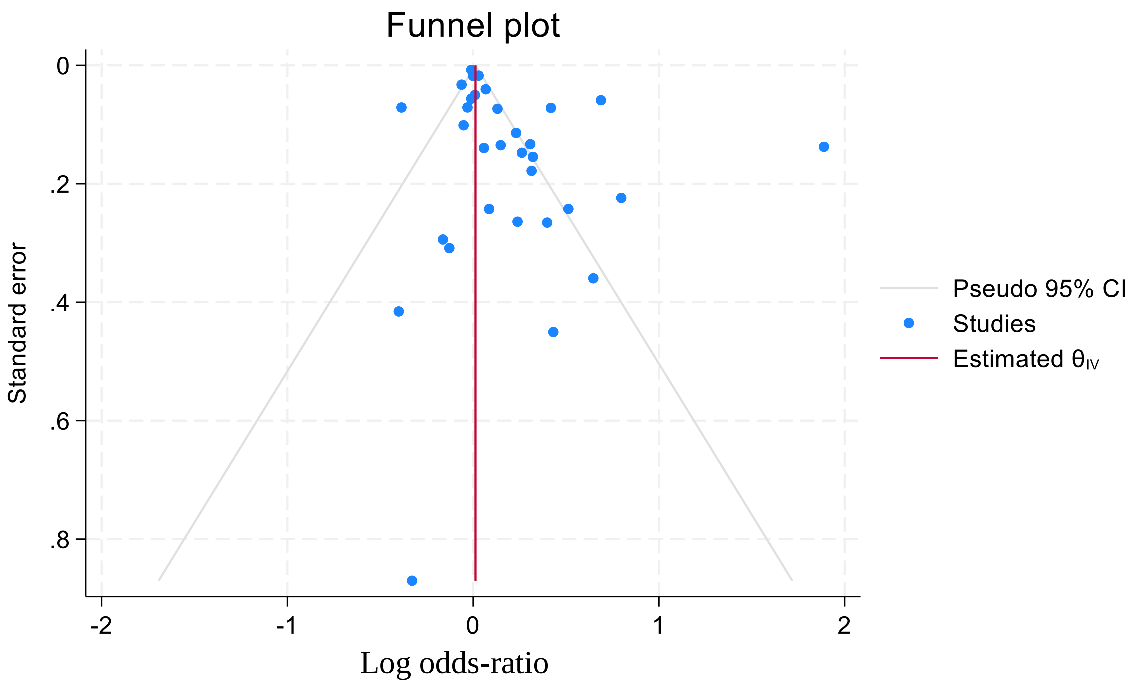

Educational information (printed – unspecified literacy)

Log odds-ratio

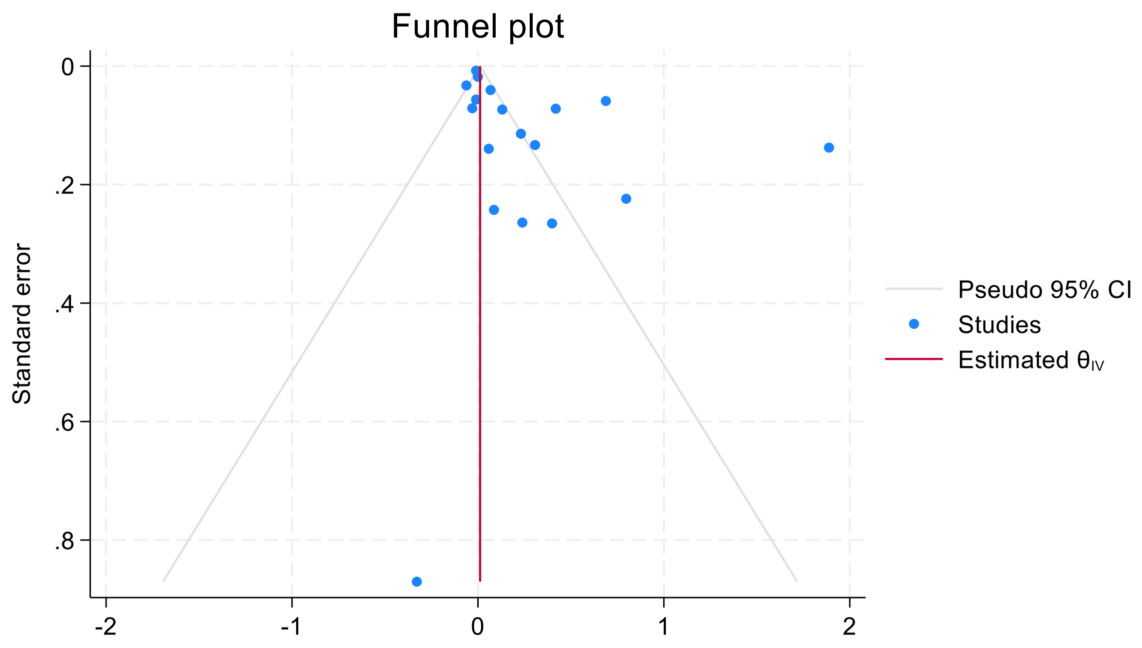

Letters (overall)

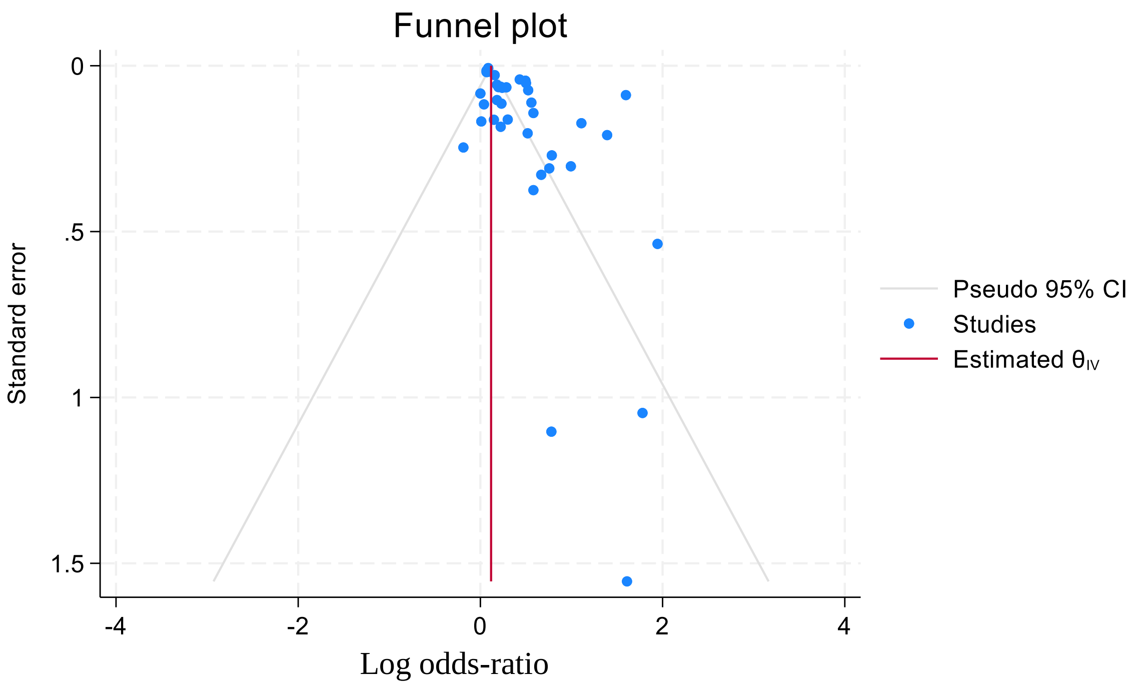

Letters (Signed by GP)

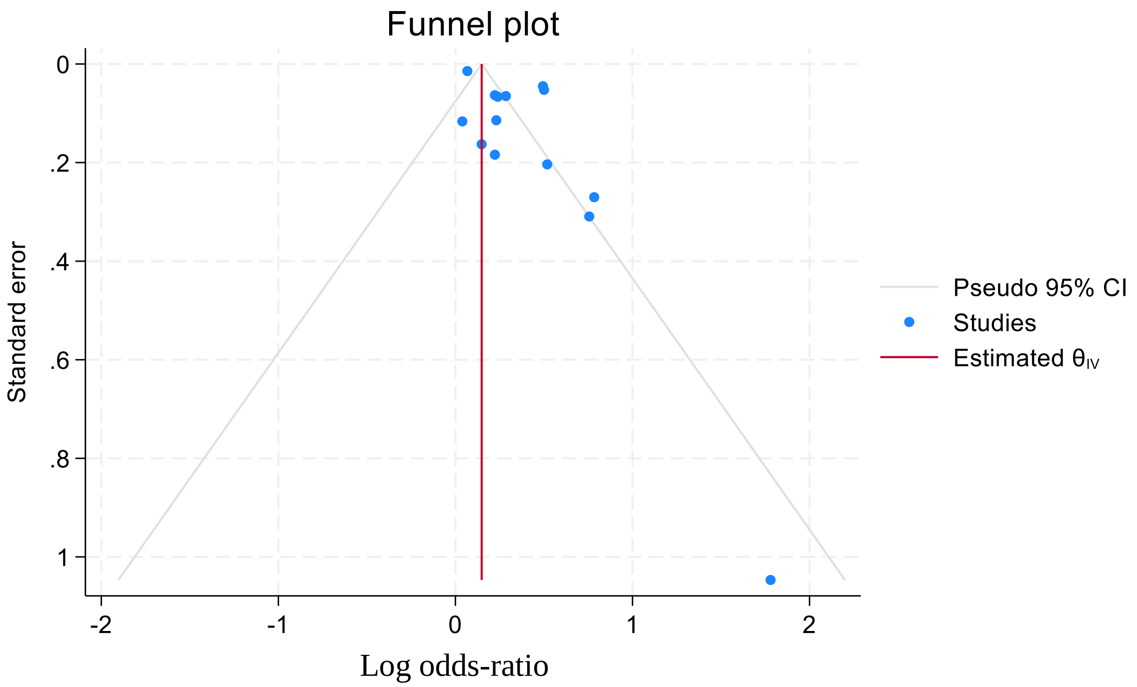

Multi-lingual approaches (overall)

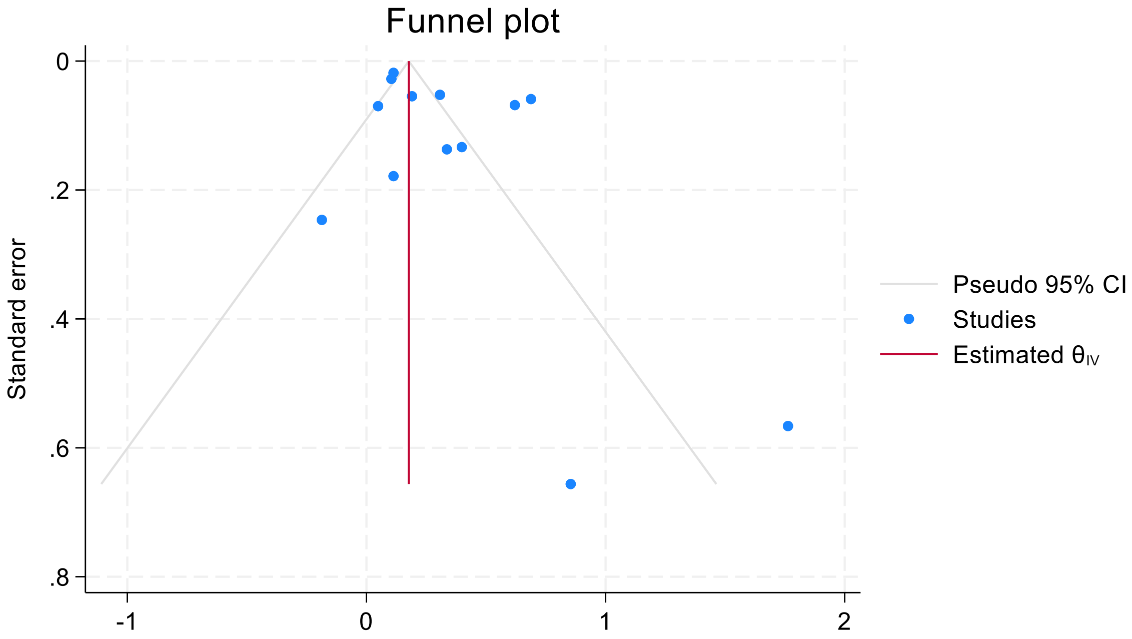

Log odds-ratio

Phone (overall)

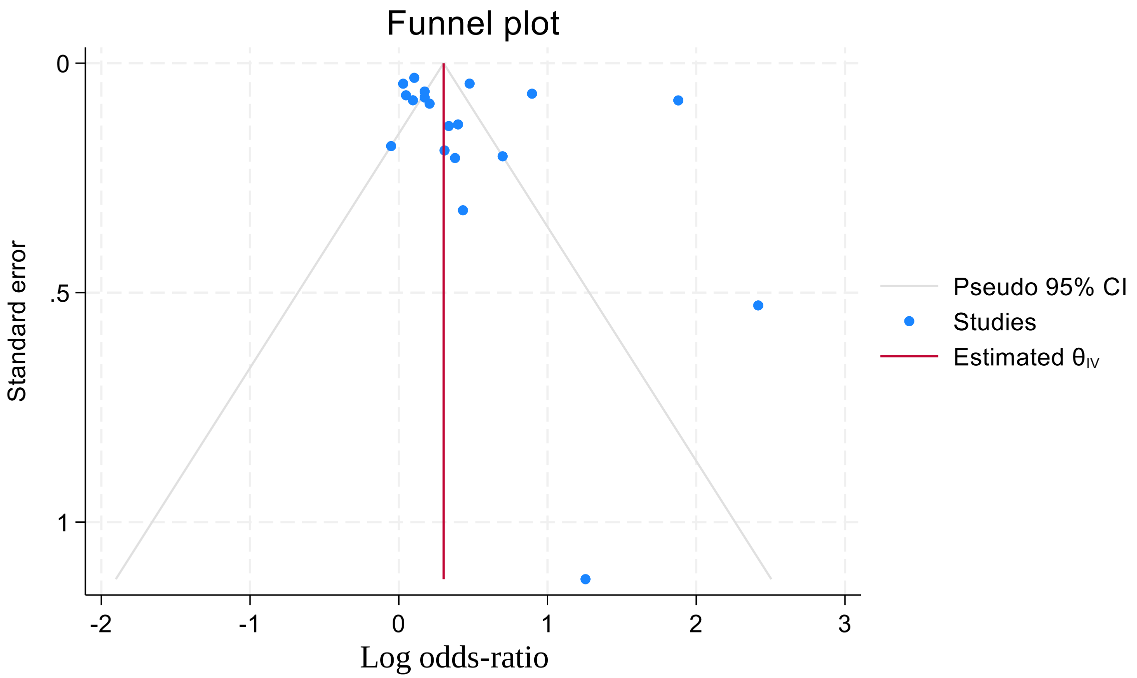

Phone (calls)

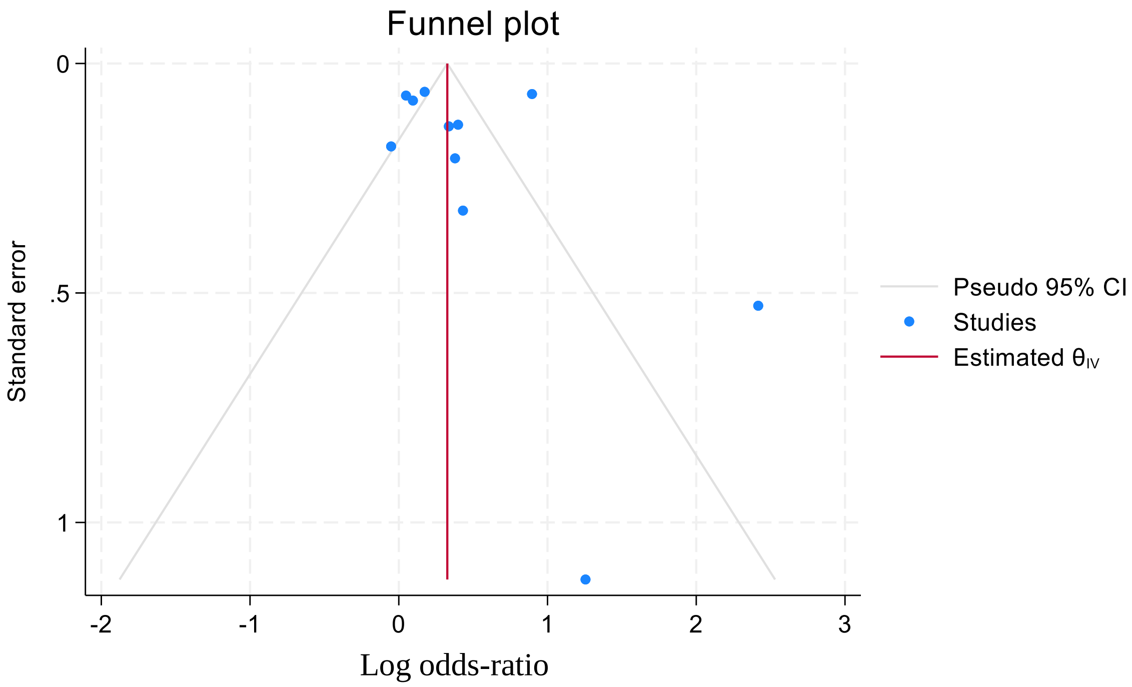
Funnel plot
Standard error
0
.5
1
-2
-1
0
1
2
3
Log odds-ratio
Pseudo 95% CI
Studies
Estimated θIV

PRISMA 2020 Checklist

Simplified methods

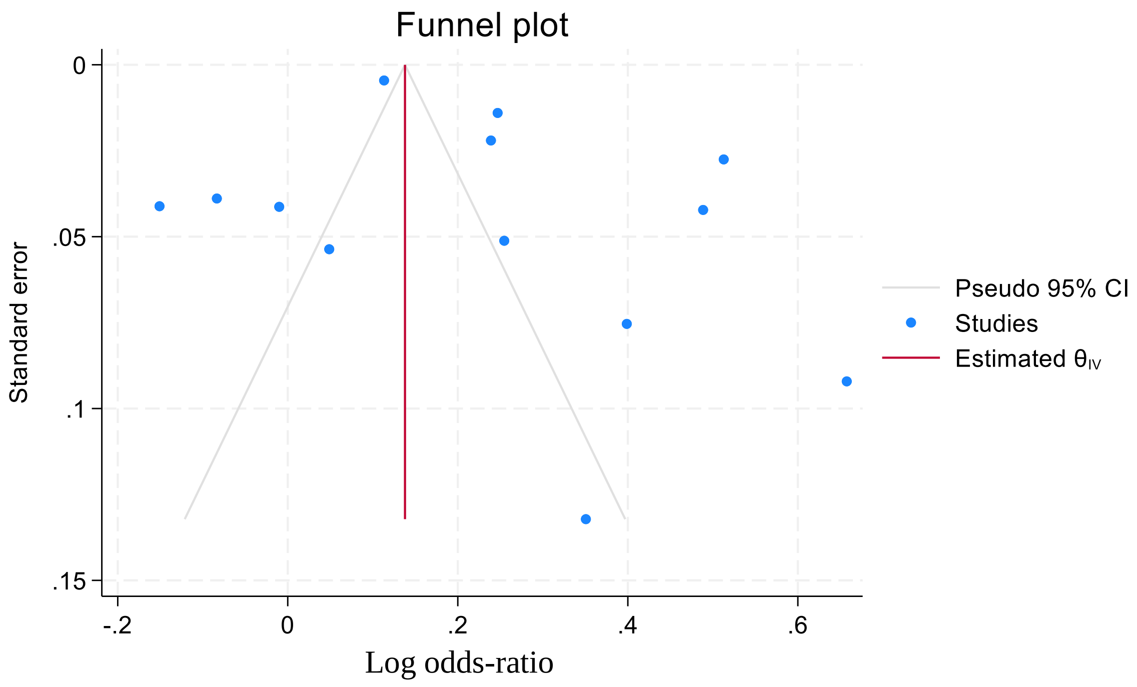

Targeted at GPs (overall)

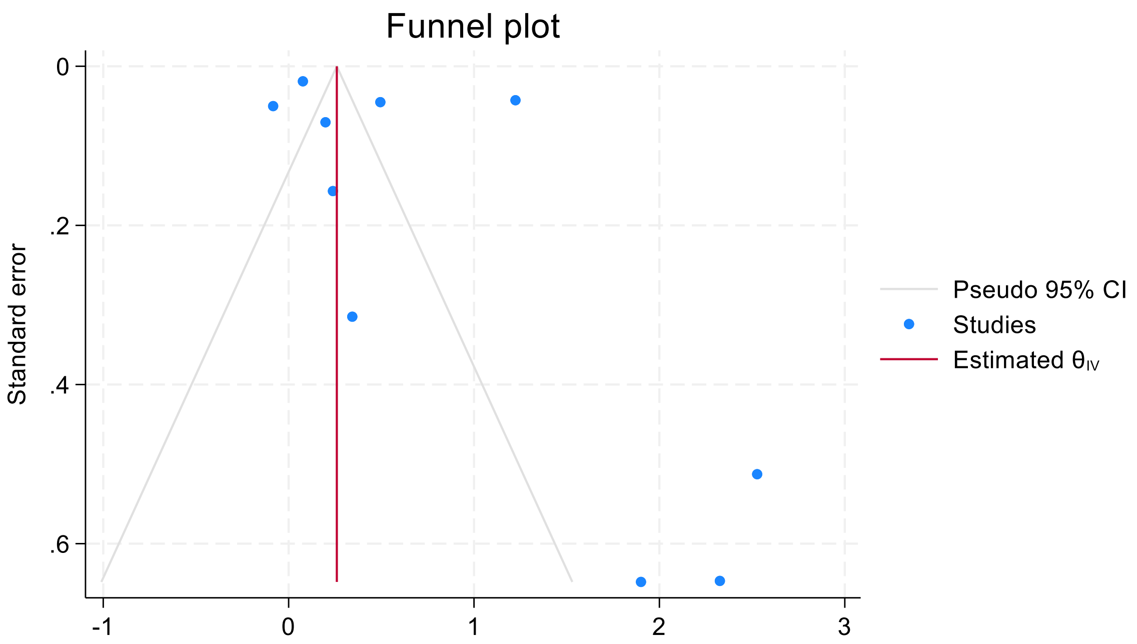

Log odds-ratio

Outcome: Knowledge

PRISMA 2020 Checklist

Decision Aids (overall)

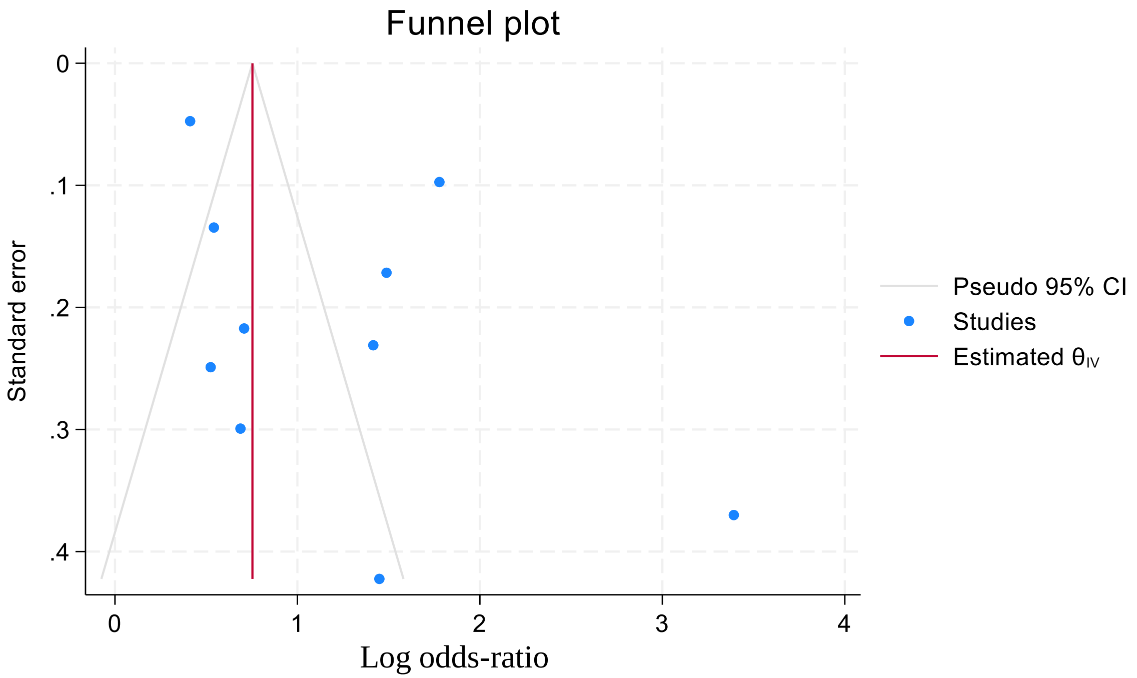

Supplement: Supplemental Material - Improving Breast Cancer Outcomes by Enhanced Activities in Early Detection and Diagnosis: An Umbrella Review and Meta-Analyses of Randomised Controlled Trials in HNotigh-Income Contexts With Universal Healthcare Coverage [file sj-pdf-1-ccx-10.1177_10732748261462921.pdf]
